# C–H arylation of triphenylene, naphthalene and related arenes using Pd/C†
†Electronic supplementary information (ESI) available. CCDC 1013416 and 1013417. For ESI and crystallographic data in CIF or other electronic format see DOI: 10.1039/c4sc03051f



**DOI:** 10.1039/c4sc03051f

**Published:** 2014-12-22

**Authors:** Karl D. Collins, Roman Honeker, Suhelen Vásquez-Céspedes, Dan-Tam D. Tang, Frank Glorius

**Affiliations:** a Westfälische Wilhelms-Universität Münster , Organisch-Chemisches Institut , Corrensstraße 40 , 48149 Münster , Germany . Email: glorius@uni-muenster.de

## Abstract

A highly selective arylation of a number of polyaromatic hydrocarbons (PAHs) with aryliodonium salts and Pd/C as the only reagent is reported. The first C–H functionalization of triphenylene is explored, and proceeds at the most sterically hindered position. This non-chelate assisted C–H functionalization extends the reactivity profile of Pd/C and provides controlled access to π-extended PAHs, an important aspect of work towards the preparation of nanographenes. Mechanistic studies suggest *in situ* formation of catalytically active insoluble nanoparticles, and that the reaction likely proceeds *via* a Pd(0)/Pd(ii) type reaction manifold.

## Abstract 2

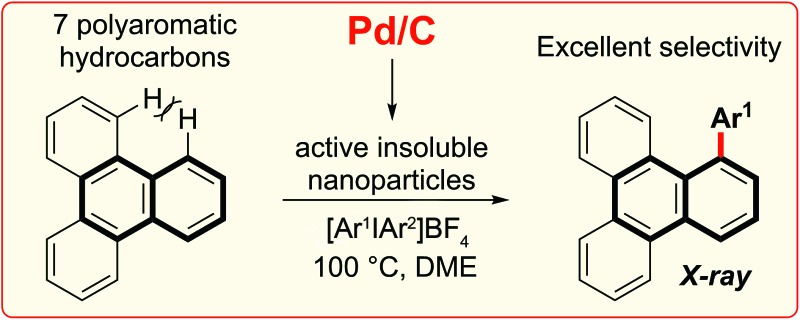
A highly selective arylation of a number of polyaromatic hydrocarbons (PAHs) with aryliodonium salts and Pd/C as the only reagent is reported.

## Introduction

Pd/C has long been established as an efficient catalyst in hydrogenation and cross-coupling reactions.^1^ Although supported catalysts are formally heterogeneous in nature, studies of Pd/C suggest it typically acts as a reservoir of homogeneous active catalytic species, following leaching of palladium from the support into the solution.^1c,1e,2^ Our recent work identified that Pd/C can mediate highly selective direct C–H functionalizations of heteroaromatic systems, representing novel reactivity beyond its traditional applications.^3^ A surprising facet of these works was that studies suggested a heterogeneous active catalytic species despite the high temperature or strongly oxidizing nature of the reaction conditions.^4,5^ Furthermore, Pd/C was clearly superior in terms of regioselectivity and yield to all homogeneous palladium sources screened, and no ligands were employed. Following these unexpected observations, we were encouraged to explore the more challenging selective C–H functionalization of non-heteroatom containing arenes,^6^ with a view to unveiling further surprising selectivity or reactions exploiting heterogeneous catalytic species.

**Scheme 1:**
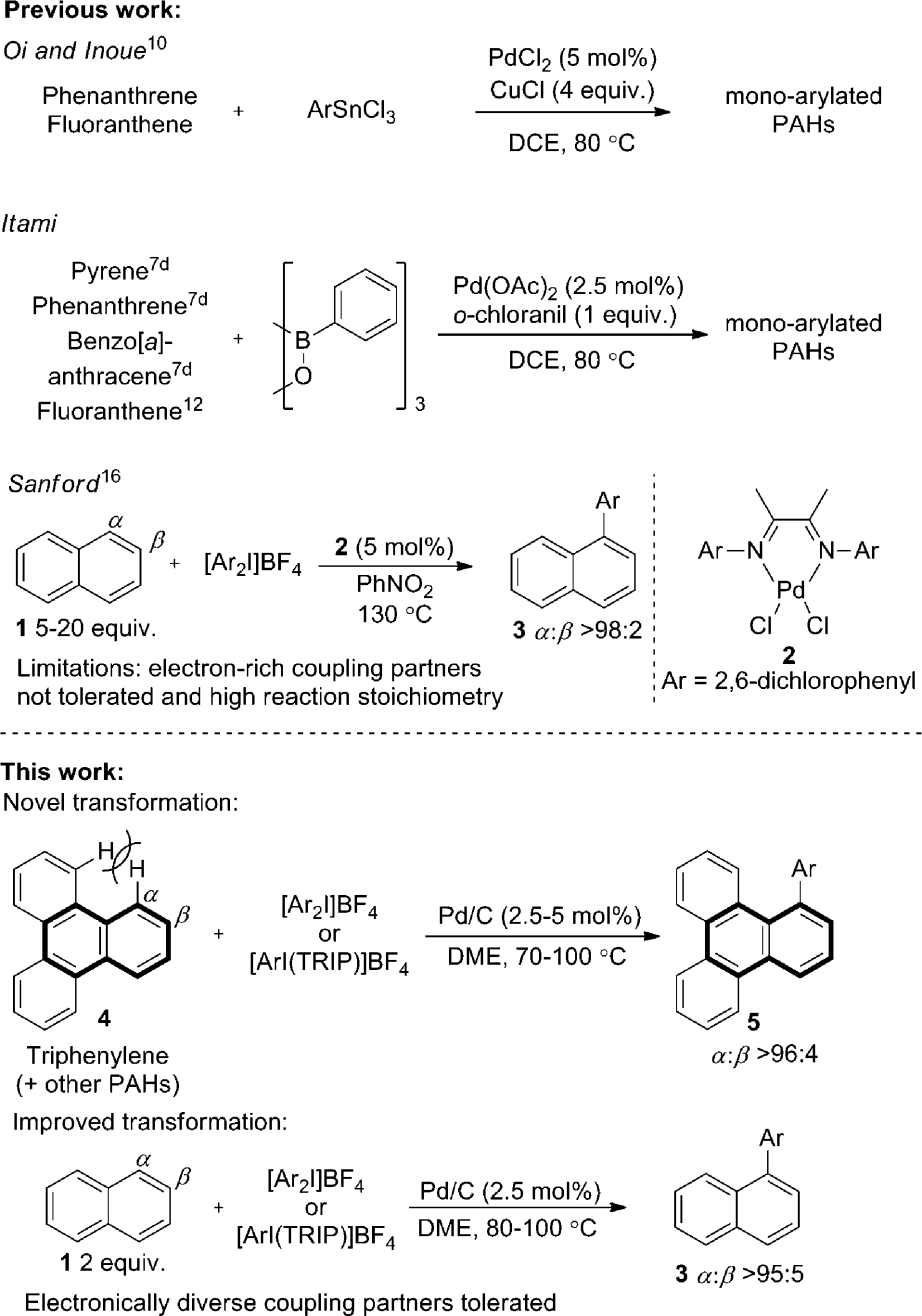
C–H functionalization of naphthalene and polyaromatic hydrocarbons (PAHs). TRIP is 2,4,6-triisopropylphenyl.

The direct arylation of PAHs is an important and unmet challenge for the ‘bottom-up’ preparation of π-extended PAHs.^7^ Controlled access to such defined graphene like two-dimensional sheets of sp^2^-hybridized carbon atoms could have significant implications for the preparation of larger graphene structures,^8^ which are of immense interest to numerous scientific fields.^9^ Despite the potential applications, only limited precedent for the direct arylation of larger PAHs is reported. Seminal work by Oi and Inoue reported an effective arylation of phenanthrene and fluoranthene with aryltin trichlorides.^10^ One example of phenanthrene arylation has also been reported by Shi.^11^ Important studies by the group of Itami demonstrated a more general solution (Scheme 1) using prepared boroxines as coupling partners.^7d^

Using Pd(OAc)_2_ and *o*-chloroanil as oxidant, they have described arylations of pyrene, phenanthrene and benzo[*a*]anthracene at the more reactive olefin like K-region^7*d*^ of these PAHs. Notably, the more challenging C–H arylation of triphenylene (**4**), which does not contain a K-region is not reported. Selective coupling of fluoranthene^12^ and the polyarylation of corannulenes^13^ and perylenes^14^ have also been demonstrated. C–H arylation of naphthalene (**1**), has been explored more extensively, though typically proceeds with moderate yields and poor *α* : *β* selectivity.^15^ A study from the Sanford group employing a bulky Pd(ii) diimine catalyst (**2**) in nitrobenzene at 130 °C represents the *only* highly selective *α*-arylation of naphthalene (**1**), and scope is limited to a small number of electron-deficient or electron-neutral aryliodonium salts as coupling partners (Scheme 1).^16,17^

In this work we first present a Pd/C (Heraeus: 5 wt% Pd/C type K-0219, <3% H_2_O) catalyzed arylation of triphenylene (**4**) with aryliodonium salts.^18^ To the best of our knowledge this represents both the first C–H functionalization of triphenylene, and a rare example of C–H functionalization of non-heteroaromatic systems using a simple heterogeneous catalyst (Scheme 1).^19^ Notably, the reaction occurs with complete selectivity at the most hindered position. The arylation of several other PAHs is also demonstrated, including the hitherto unknown direct C–H functionalization of anthracene at C1–H.^20^ This simple catalytic system also enabled a highly selective arylation (*α* : *β* > 95 : 5) of naphthalene (**1**) with electronically diverse diaryliodonium salts. Empirical data suggests the *in situ* generation of catalytically active insoluble nanoparticles from a Pd/C precatalyst, and further mechanistic studies support a Pd(0)/Pd(ii) reaction manifold.

## Results and discussion

**Scheme 2:**
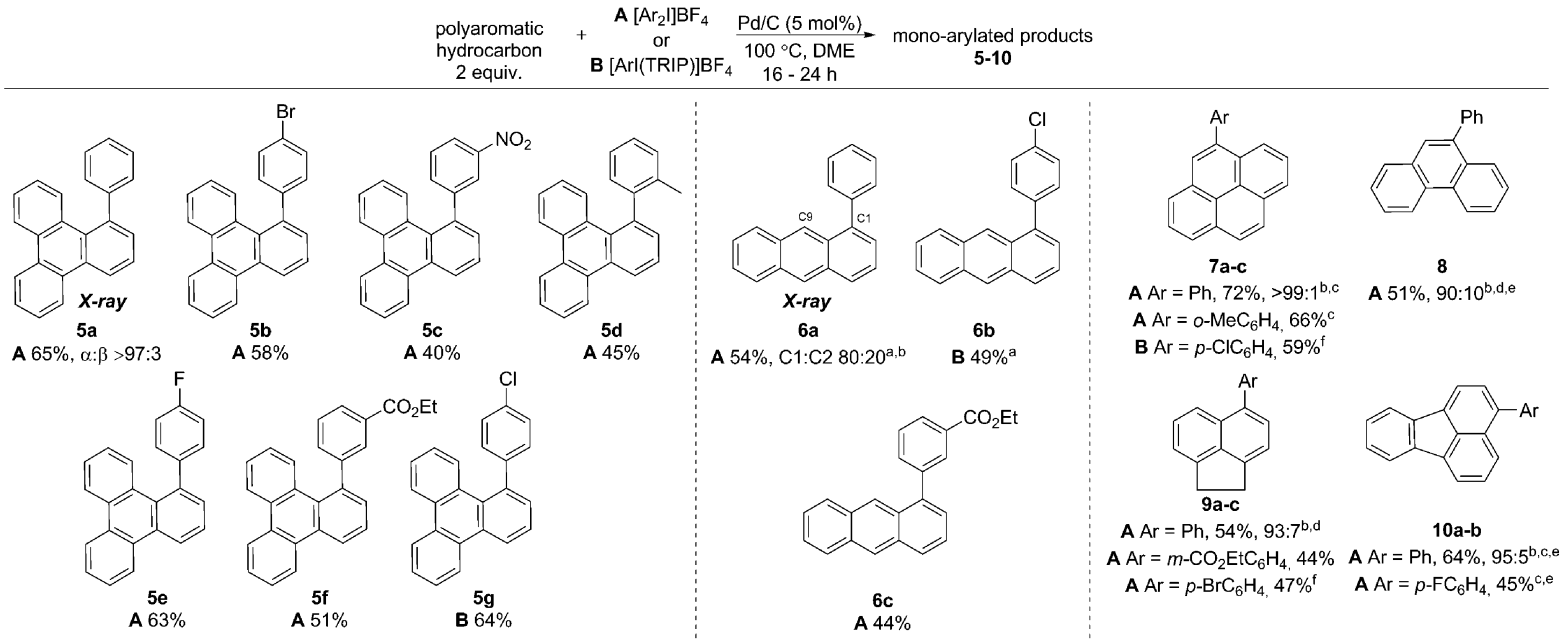
Arylation of larger polyaromatic hydrocarbons. Yields given are isolated yields for the major regioisomer unless noted otherwise. ^a^Conditions: anthracene (5 equiv.), Pd/C (2.5 mol%), **A** at 80 °C and **B** at 100 °C. ^b^Ratio of major regioisomer to any other regioisomers. ^c^Conditions: 80 °C, Pd/C (2.5 mol%). ^d^Conditions: 70 °C, Pd/C (2.5 mol%). ^e^Yield is for major isomer only, though isolated as a mixture. ^f^Conditions: 100 °C, Pd/C (2.5 mol%). TRIP is 2,4,6-triisopropylphenyl.

**Scheme 3:**
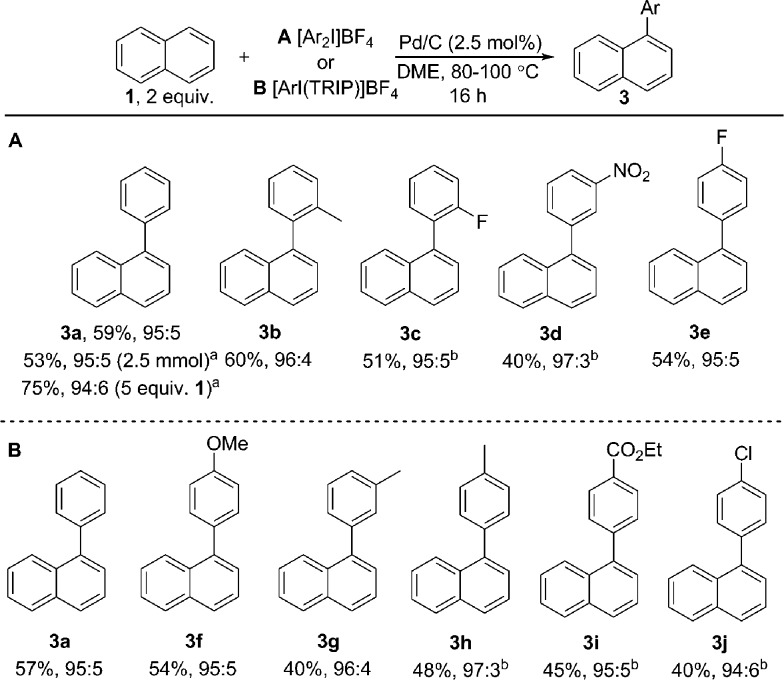
Scope of naphthalene arylation. Yields given are isolated yields for the major regioisomer. All ratios refer to *α* : *β*. ^a^GC yield. ^b^Reaction temperature 100 °C. TRIP is 2,4,6-triisopropylphenyl.

Initially exploring the arylation of simple arenes using Pd/C, we found that subjecting naphthalene (**1**) to our previously reported conditions^3*b*^ identified traces of an arylated product. Following an initial solvent screen, we then explored the reactivity of larger PAHs and were very excited to observe a highly selective arylation of triphenylene (**4**). Optimization established that 5 mol% of Pd/C in DME at 100 °C gave **5a** in 65% yield with excellent *α* : *β* selectivity (>97 : 3), with no efforts made to exclude air or moisture. The suppression of polyarylation in direct C–H functionalization of PAHs is still an unmet challenge, and accounts in part for the remaining material. X-ray studies confirmed that the arylation occurs at the significantly more hindered position. The scope of the reaction was explored and we were pleased to find that this challenging transformation proceeds effectively with a range of functionalized diaryliodonium salts (Scheme 2). The diversity of functionalities tolerated in the reaction provides multiple options for further derivatization of these novel structures towards more complex π-extended systems. The arylation of related π-extended PAHs was also demonstrated: we observed an unprecedented selectivity for the arylation of anthracene at the C1–H position, as confirmed by X-ray crystallographic analysis. The K-region^7*d*^ of pyrene and phenanthrene is preferentially arylated to give **7** and **8** respectively. The high yields observed for the mono-arylation of pyrene when compared to the literature precedent are particularly noteworthy considering the value of these structures.^7*d*^ Selective arylation of the zigzag edge of both acenaphthene and fluoranthene to give **9** and **10** respectively, was also achieved. For the arylation of naphthalene (**1**) a reduced catalyst loading of 2.5 mol% was employed, with no additional reagents or ligands required to achieve consistently excellent *α* : *β* selectivities of >94 : 6 (Scheme 3). We observed synthetically useful yields with only 2 equivalents of naphthalene, providing practical access to arylated products; previous studies^15–17^ have typically employed 5–100 equivalents of naphthalene to achieve acceptable yields, which significantly impedes purification. If required, the yield of the reaction can be further increased by using a greater excess of naphthalene. Notably, this simple catalytic system enabled the reaction of electronically diverse iodonium salts, with *o*-substitution and unsymmetrical iodonium salts containing a cheap, non-transferable arene (2,4,6-triisopropylphenyl) also well tolerated.

We have also investigated the reaction of a large number of substituted naphthalenes, though unfortunately we observed either no reactivity or poor selectivity (see ESI† for all examples). Electronically distinct arylated naphthalenes **3a**, **3f** and **3i** were subjected to the reaction conditions a second time to identify any electronic influence on the selectivity of a second arylation, though all substrates yielded a near identical distribution (7 : 3) of bis-arylated products. Despite significant effort, we were unable to isolate bis-arylated triphenylene or anthracene structures: mono-arylated compounds were subjected to the reaction conditions for a second time resulting in conversion of the starting materials, though gave intractable mixtures of compounds.

### Heterogeneity studies

Our preliminary experiments in this study were indicative of a reaction mechanism distinct to that of our previously reported C–H functionalization of heteroaromatic systems using Pd/C and aryliodonium salts.^3*b*^ Consequently, investigation of the hetero- or homogeneous nature of the active catalytic species and the reaction mechanism were undertaken to gain further insight. Studying the arylation of triphenylene (**4**) and naphthalene (**1**) with [Ph_2_I]BF_4_ we investigated the nature of the active catalytic species. A heterogeneous catalytic species was indicated by several established methods:^1d,4,21^

(1) A standard Hg-poisoning test resulted in inhibition of the reaction, suggesting the active catalytic species is likely to be heterogeneous in nature.

(2) A hot filtration test indicated that all active catalytic species are removed by the filtration process, which is consistent with a catalytically active heterogeneous species.

(3) Several three-phase tests using polymer-bound naphthalene were undertaken, and in no instance was any arylated product detected on the polymer support.^22^ Should Pd/C act simply as a reservoir for homogeneous palladium, reaction of the supported naphthalene would be expected. Control experiments indicated that the support does not inhibit reaction.

While these methods for evaluating the nature of the active catalyst have individual limitations, taken as a whole these results are strongly indicative of a catalytically active heterogeneous component. Considering the high temperature and oxidative nature of our reaction conditions it is probable that palladium does leach from the support into solution,^1c,1e,2^ though does not generate catalytically active *homogeneous* palladium species. These experiments provide no indication of whether the heterogeneous species is supported on carbon or not.

### Preliminary studies of the active catalytic species

**Fig. 1:**
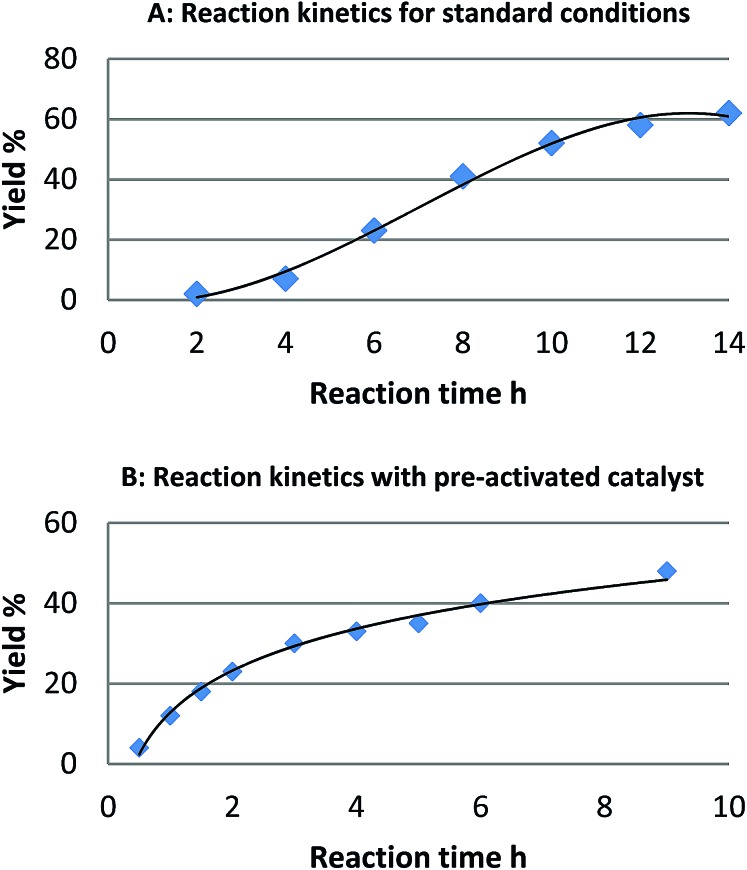
Reaction profile for the reaction of naphthalene (**1**) and [Ph_2_I]BF_4_: (A) standard reaction conditions and (B) after pre-activation of the catalyst with (diacetoxy)iodobenzene.

For the reaction of naphthalene, kinetic studies indicated a significant induction period and a sigmoidal reaction profile,^23^ which is consistent with the formation of the active catalytic species *in situ* (Fig. 1). Leaching of palladium into solution and formation of homogeneous active catalytic species could account for this observation, though is contrary to our studies. Consequently, we investigated the potential origins of this induction period.

We found that pretreatment of the catalyst under the standard reaction conditions for 2 h with diaryliodonium salts *or* (diacetoxy)iodobenzene prior to the introduction of naphthalene, resulted in a significant reduction of the induction period. We hypothesized that pretreatment of the catalyst with these strong oxidants may result in oxidation of Pd(0) to Pd(ii) and initiation of a Pd(ii)/Pd(iv) catalytic cycle as proposed by Sanford.^16^ Alternatively, a physical modification of Pd/C resulting in the formation of the active catalyst may also account for the induction period. To investigate the likelihood of a Pd(ii)/Pd(iv) catalytic cycle we investigated the reactivity of six homogeneous or heterogeneous Pd(ii) sources for the arylation of naphthalene (Table 1).

**Table 1:** Screening of Pd(ii) catalysts for reactivity^*a*^

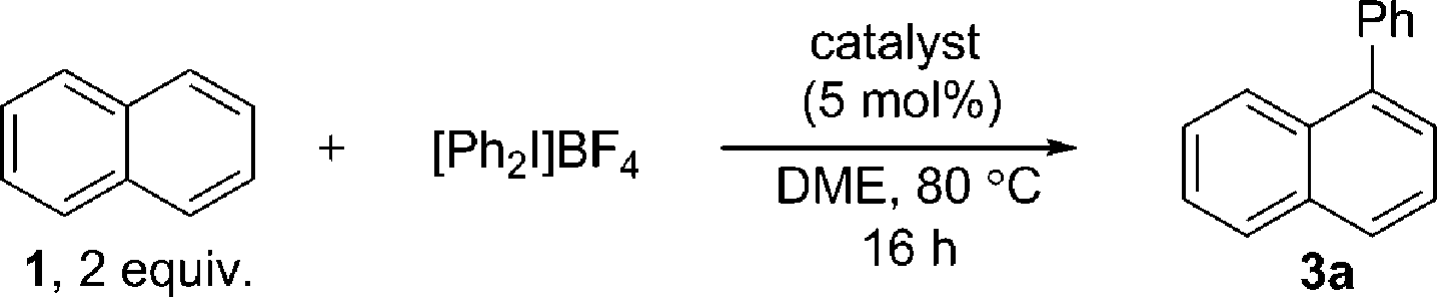		
Catalyst	Yield^*b*^ %	*α* : *β* ^*b*^ selectivity
K_2_PdCl_4_	8	95 : 5
PdCl_2_(dppe)	3	82 : 18
PdCl_2_(PPh_3_)_3_	9	96 : 4
Pd(OH)_2_/C^*c*^	2	nd
Pd(OH)_2_/Fe_3_O_4_ ^*c*^	12	96 : 4
Pd(OAc)_2_	52	93 : 7

^*a*^Standard conditions: naphthalene (0.2 mmol), [Ph_2_I]BF_4_ (0.5 equiv.), catalyst 5.0 mol%, DME (1 mL), 80 °C, 16 h.

^*b*^Determined by GC-FID.

^*c*^2.5 mol% catalyst. nd is not determined.

With the exception of Pd(OAc)_2_ only negligible reactivity was identified, which does not support that the catalytic cycle is initiated by a Pd(ii) species. The reactivity observed with Pd(OAc)_2_ can readily be explained by the well-established thermal reduction of Pd(OAc)_2_ to form catalytically active heterogeneous Pd(0) nanoparticles.^24^ Kinetic studies employing Pd(OAc)_2_ as the catalyst demonstrated an induction period consistent with the generation of nanoparticles, and the formation of heterogeneous material could be observed visually. Further comparative studies (*vide infra*) support this hypothesis. A preliminary analysis of the results presented so far indicates that the activation of the catalyst with (diacetoxy)iodobenzene or an iodonium salt is unlikely to be due to an oxidation of Pd(0) to Pd(ii)/Pd(iv). The formation of a new heterogeneous Pd(0) species following leaching of Pd into solution^25^ possibly *via* an Ostwald ripening mechanism,^26^ or catalyst modification without leaching of the metal could account for the observations reported. The reaction is shown to be first order in palladium, and a reduced induction period with increased catalyst loading is also observed. These results are consistent with the formation of the active catalyst being rate-limiting.

A more extensive catalyst screening was undertaken, importantly highlighting significant differences in the reactivity of several commercial Pd/C sources. Interestingly, we found that in addition to Pd/C and Pd(OAc)_2_, Pd/Al_2_O_3_ proved a proficient catalyst for arylation, and we subsequently undertook a short comparison of all three systems (Table 2).

**Table 2:** Comparative study of catalysts for the arylation of naphthalene^*a*^

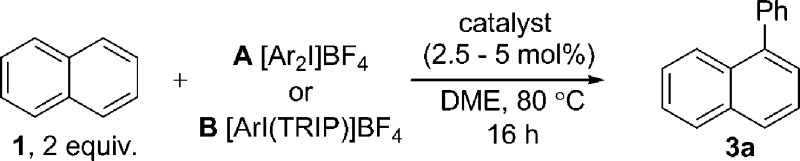			
	Pd/C	Pd(OAc)_2_ ^*c*^	Pd/Al_2_O_3_
Hg poisoning	*het*	*het*	*het*
Hot filtration test	*het*	*het*	*het*
Standard reaction	63%	52%	64%
5 equiv. **1**	75%	50%	79%
Reaction with [*p*-MeOPhI(TRIP)]BF_4_	54%^*b*^	30%	Trace
Reaction with [*o*-MePh_2_I]BF_4_	60%^*b*^	nd	Trace

^*a*^Standard conditions: naphthalene (**1**) (2 equiv.), iodonium salt (1 equiv.), catalyst (2.5 mol%), DME (0.1 M), 80 °C, 16 h. Yields given are GC yields for the major regioisomer unless noted otherwise. *het* indicates that the results suggest a heterogeneous active catalytic species.

^*b*^Isolated yield..

^*c*^5 mol% of catalyst used.

For all catalysts Hg poisoning and hot filtration studies indicate a heterogeneous active catalytic species, supporting the earlier hypothesis that Pd(OAc)_2_ is reduced to Pd(0) nanoparticles under the reaction conditions. When 5 equivalents of naphthalene are used, both supported systems give a higher yield than in the standard reaction, but with Pd(OAc)_2_ this is not the case. This indicates that the presence of a support is not crucial for reactivity but it appears to increase the turnover number of the catalyst. Finally, although Pd/Al_2_O_3_ proved highly proficient for the standard reaction, only trace reactivity was observed with electron-rich or *ortho*-substituted arenes. It would appear that while several palladium sources can mediate the basic transformation, Pd/C is typically superior.

### Reaction mechanism

While postulating traditional ‘homogeneous’ type reaction mechanisms for processes involving nanoparticles is unlikely to be reflective of the true processes, we sought we investigate the feasibility of a number of reaction pathways:

(1) Undirected C–H activation of naphthalene/triphenylene.^6*y*^

(2) Oxidation of Pd(0) to Pd(ii) and initiation of Pd(ii)/Pd(iv) reaction manifold.^11^

(3) Radical reaction pathways.^16^

(4) Insertion of an ArPd(ii)X species.^15*a*^

(5) Electrophilic attack of naphthalene/triphenylene (S_E_Ar/electrophilic palladation).^7d,27^

**Scheme 4:**
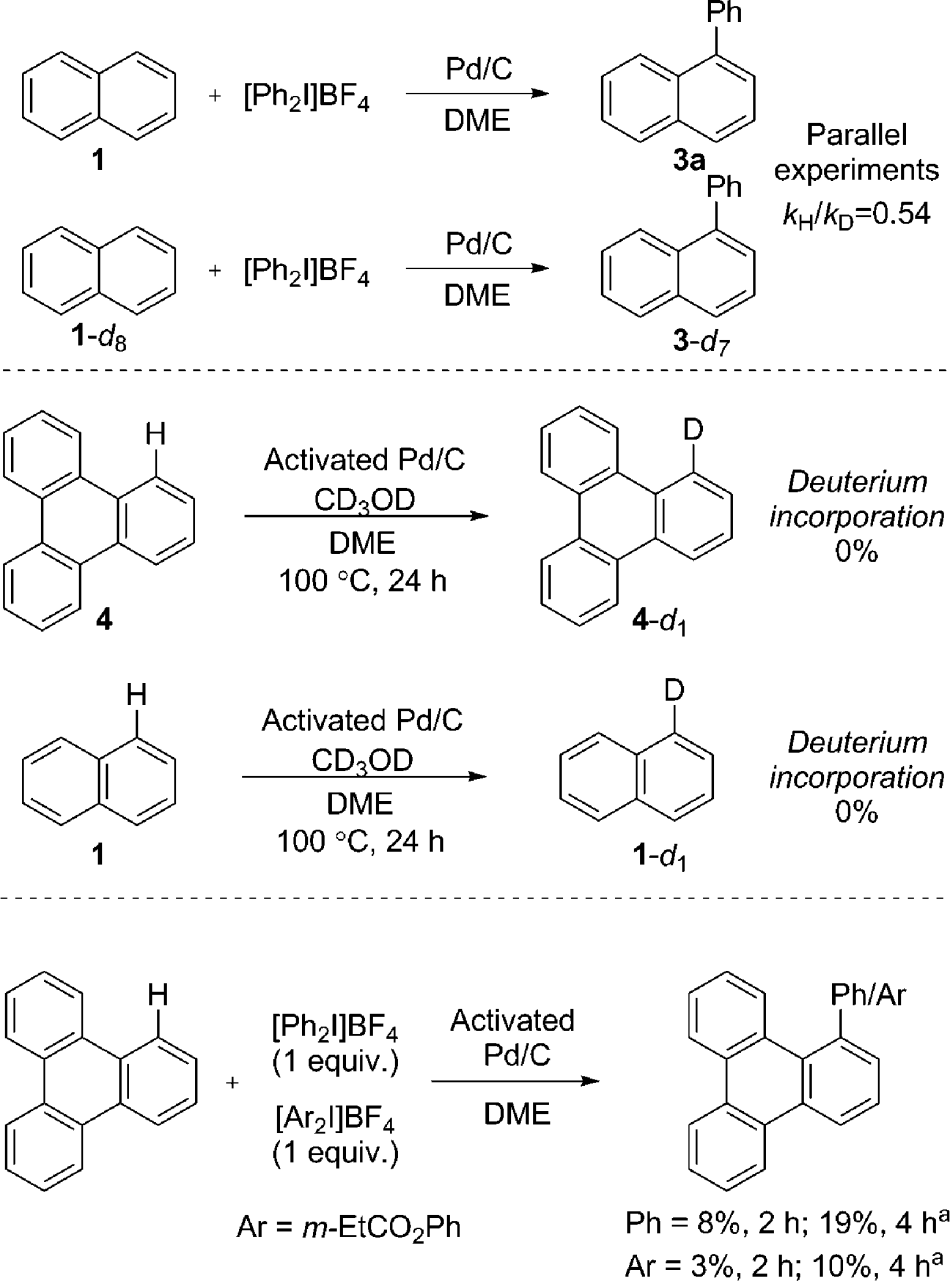
Determination of KIE, deuteration and competition experiments. ^*a*^ GC yield.

We immediately discounted a mechanism that proceeds initially *via* an undirected C–H activation. Firstly, an unusual inverse kinetic isotope effect (KIE) of 0.54 was determined by parallel initial rate studies of naphthalene and *d*_8_-naphthalene, ruling out a rate-limiting C–H activation (Scheme 4).^28^ Secondly, the reaction is shown to be zero-order in naphthalene precluding its involvement in the rate-determining step. Finally, deuteration experiments employing pre-activated catalyst indicated no deuterium incorporation into naphthalene or triphenylene, suggesting that a Pd(0) catalyzed C–H activation is unlikely.

**Fig. 2:**
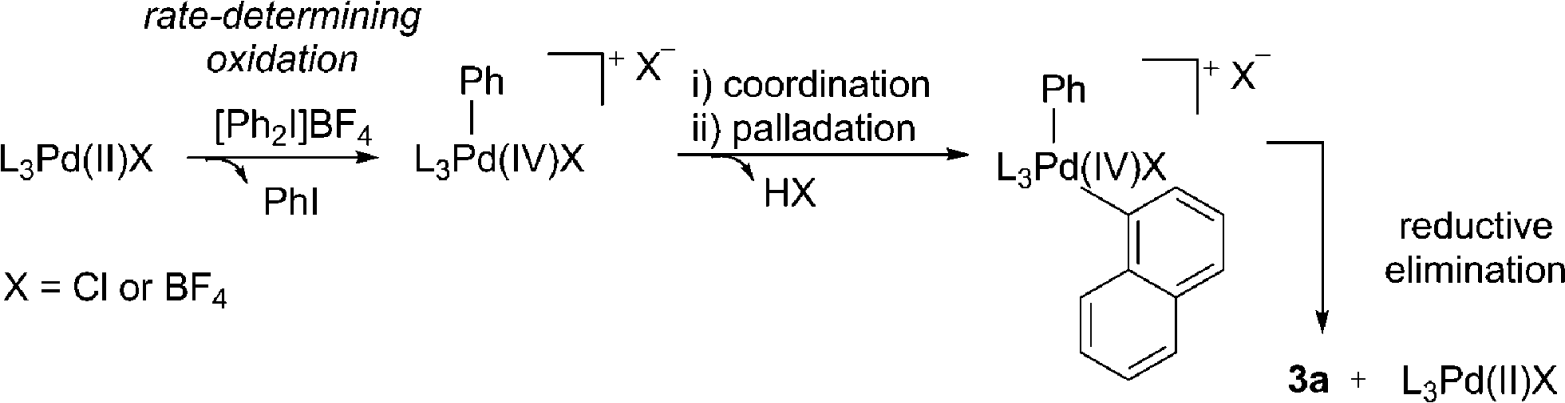
Pd(ii)/Pd(iv) reaction manifold proposed by Sanford.

We next considered a Pd(ii)/Pd(iv) manifold for the arylation of naphthalene (**1**) with diaryliodonium salts as proposed by Sanford^16^ (Fig. 2). Sanford proposed a rate-determining oxidation of Pd(ii) to Pd(iv) accounts for the failed reaction of electron-rich iodonium salts. In contrast to her report, our studies show facile reaction of electron-rich iodonium salts, with electron-deficient coupling partners often requiring higher temperatures. A competition experiment also demonstrated more electron-rich iodonium salts react more rapidly, indicating a rate-limiting oxidation of palladium is unlikely (Scheme 4). Furthermore, our described catalyst activation studies suggested entry into a Pd(ii)/Pd(iv) catalytic cycle was unlikely. An alternative rate-limiting reductive elimination form Pd(iv) is also improbable.

**Fig. 3:**
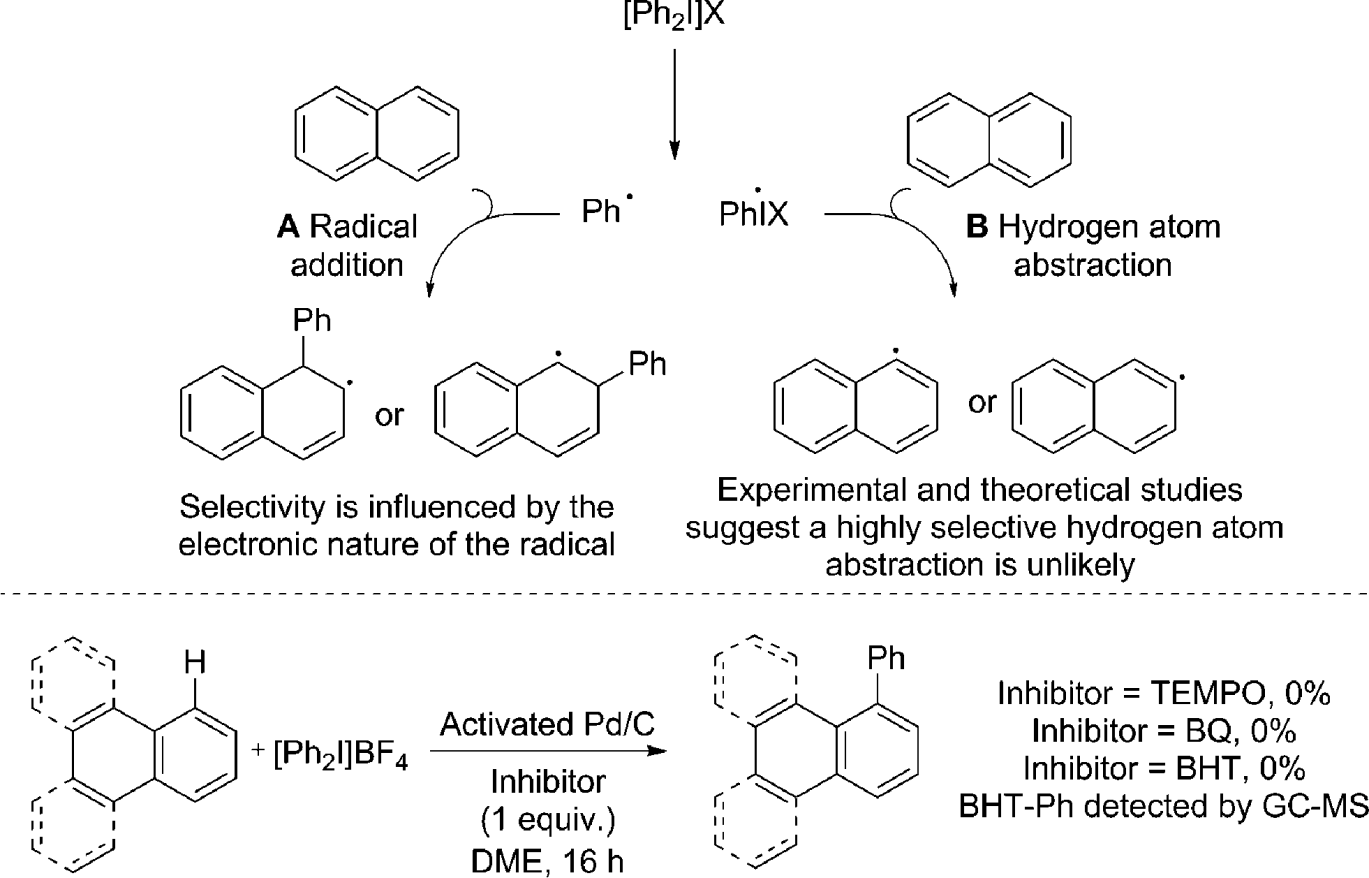
Previously proposed radical pathways for the arylation of naphthalene; radical inhibitor studies.

Radical coupling pathways have been proposed by Rodríguez for related reactions (Fig. 3).^15*a*^ In our studies we have found that the reaction is inhibited by TEMPO, BHT and BQ which is consistent with a radical component within the reaction mechanism (Fig. 3). Identification of a Ph–BHT adduct further supports this proposition, and suggests that an aryl radical is derived from the iodonium salt. We feel that direct addition of aryl radicals to naphthalene (Fig. 3, **A**) is unlikely in this instance, as this addition is well precedented to occur with regioselectivities dependent on the electronic nature of the aryl radical, something we do not observe.^15a,29^ The formation of radical species *via* hydrogen atom abstraction (Fig. 3, **B**) is also unlikely when bond dissociation energies (BDE) and/or energetics of the radical formation are considered.^30^ Firstly, the C–H BDE of benzene is highly comparable to that of naphthalene and therefore would be likely to react under these reaction conditions.^30a,b^ Under our conditions several monoarenes gave only trace reactivity. Secondly, as selectivity of the hydrogen atom abstraction will be governed by the BDE/stability of the formed radical, arylation of anthracene would be expected at the bis-benzylic C9–H in contrast to the C1–H selectivity observed in our study (Scheme 2).^30a,c,d^ The relative BDE for the *α* and *β* C–H bonds in naphthalene are still debated, though are very similar and selectivity on this basis seems unlikely.^30^ Consequently we propose should a radical process be in operation, palladium is nevertheless involved in the bond forming step.

**Fig. 4:**
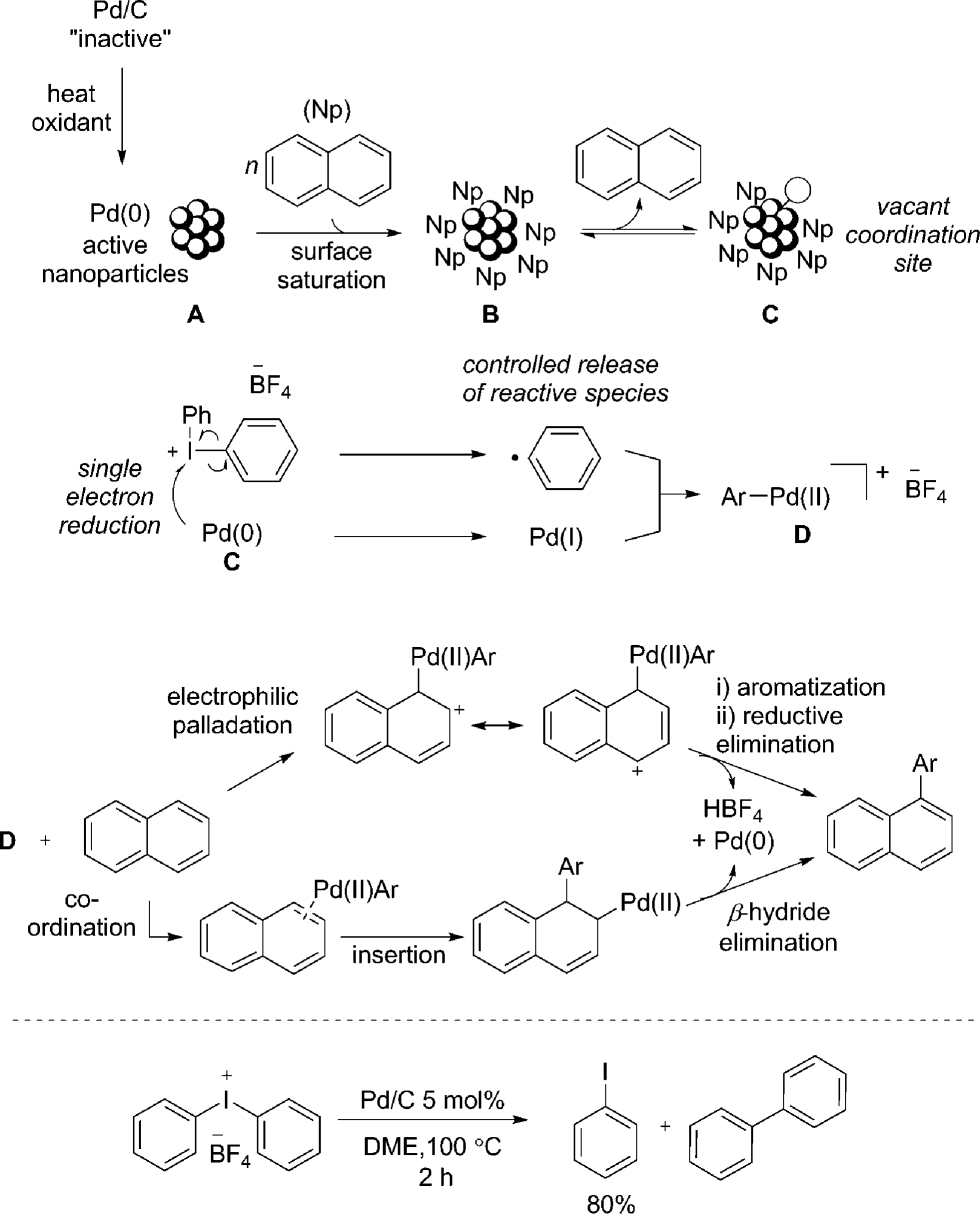
Proposed reaction mechanisms for the arylation of PAHs. Naphthalene is used as a representative substrate. Counterions omitted for clarity when required; treatment of [Ph_2_I]BF_4_ with Pd/C in the absence of a coupling partner.

Based on additional experiments and the data presented so far, a speculative mechanism using naphthalene as a representative substrate is proposed (Fig. 4). As previously noted, it is important to recognize the limitations in attributing homogeneous type reaction mechanisms to heterogeneous processes. We hypothesize that Pd/C acts as a metal reservoir, and following leaching of Pd from the support, catalytically proficient insoluble nanoparticles **A** are formed. While we cannot rule out modification of the catalyst without leaching of palladium, the product formation when employing Pd(OAc)_2_ indicates that the support is not essential for reactivity. Favorable interactions between extended π-systems and nanoparticles suggest saturation of the surface to give **B** is likely, particularly in the presence of a large excess of naphthalene (**1**). Experimental evidence supports this hypothesis: under the standard reaction conditions PhI is generated throughout the duration of the reaction in proportion to product formation. In contrast, when [Ph_2_I]BF_4_ is treated with catalytic Pd/C in the absence of the arene, 80% yield of PhI is identified after only 2 h (Fig. 4). This result suggests that naphthalene inhibits the direct reaction of the diaryliodonium salt and Pd. That the reaction is shown to be zero-order in naphthalene is also consistent with saturation type kinetics associated with heterogeneous catalysis in the presence of a large excess of reagent (pseudo first-order conditions).^31^

The creation of a vacant coordination *via* reversible dissociation of naphthalene to give species **C**, would then enable reaction with the iodonium salt. Single electron reduction of the iodonium salt results in a controlled release of a phenyl radical, and recombination with Pd(i) would generate a formal Pd(ii) cationic species **D**. Single electron processes involving hypervalent iodine(iii) reagents are well established^32^ and the ability of Pd to partake in single electron processes is widely discussed.^33^ Furthermore, our mechanistic studies are consistent with the formation of an aryl radical, though no evidence for a free radical in solution has been established. A 2-electron oxidative addition process to give **D** directly from Pd(0) and the iodonium salt cannot be ruled out. As discussed, the presence of polyaromatic species appears to inhibit the reaction of diaryliodonium salt and Pd; an important consequence of this appears to be the controlled release of a reactive phenyl species, helping to minimize the formation of biphenyl. Unlike in the reaction of only [Ph_2_I]BF_4_ and Pd/C (Fig. 4), trace amounts of biphenyl are formed under the standard reaction conditions, suggesting a low effective concentration of the reactive phenyl component. Following formation of an Ar–Pd(ii) species **D**, a facile electrophilic palladation followed by re-aromatization and reductive elimination, or an insertion and subsequent β-hydride elimination would provide access to the product. Similar mechanisms have been proposed by Itami for the arylation of pyrene.^7*d*^

The selective transfer of the least sterically hindered arene when employing unsymmetrical diaryliodonium salts is consistent with an electrophilic arylation using transition metal catalysis,^27^ though does not rule out an insertion pathway. Alternatively, the reactivity of naphthalene in contrast to simple mono-arenes could be accounted for when considering an insertion pathway. It is reported that naphthalene has a greater propensity than simple benzenes to form η^2^-complexes with metals, a prerequisite for activation of the arene towards insertion.^34^ Finally, as the reaction is shown to be zero-order in naphthalene, neither palladation process would be rate-limiting, though the unusual inverse KIE is yet to be accounted for. Although the fundamental origin is unclear, Perrin has demonstrated decreasing acidity of benzoic acids and phenols for deuterated species relative to the corresponding proteated compounds.^35^ This would suggest an effect equivalent to increasing the electron-density at the oxygen atom. Consequently, a slightly faster reductive elimination or β-hydride elimination when employing **1**-*d*_8_ due to the cumulative effect of several deuterium atoms increasing the electron-density at palladium could account for the unusual KIE, should this be the rate-limiting step of the catalytic cycle. Inverse KIEs for reductive eliminations are reported.^36^

## Conclusions

We have reported the first general C–H arylation of triphenylene. This reaction proved applicable to several other PAHs and naphthalene, and without recourse to ligands or other additives provides a practical and highly selective arylation using electronically and sterically diverse aryliodonium salts. Mechanistic studies are consistent with the formation of insoluble nanoparticles following leaching of Pd into solution from Pd/C. The reaction has been demonstrated as likely to proceed *via* a Pd(0)/Pd(ii) manifold rather than the commonly proposed Pd(ii)/Pd(iv) often cited for similar transformations.
