# Supplementary material for: C–H arylation of triphenylene, naphthalene and related arenes using Pd/C
Source: Chem Sci. 2014 Dec 22;6(3):1816–24. doi: 10.1039/c4sc03051f (PMC5649329; doi:10.1039/c4sc03051f)
Supplement: Supplementary file 1 [file SC-006-C4SC03051F-s001.pdf]

Electronic Supplementary Material (ESI) for Chemical Science.
This journal is © The Royal Society of Chemistry 2015

C–H Arylation of Triphenylene, Naphthalene and Other Arenes using Pd/C

Karl D. Collins, Roman Honeker,[‡] Suhelen Vásquez-Céspedes,[‡] Dan-Tam D. Tang, and Frank Glorius*

Westfälische Wilhelms-Universität Münster, Organisch-Chemisches Institut, Corrensstraße 40, 48149, Münster, Germany

1.0 GENERAL EXPERIMENTAL 3

2.0 SUBSTRATE SYNTHESIS 5

2.1 Preparation of Iodonium Salts 5

3.0 EXPERIMENTAL AND CHARACTERIZATION OF REACTION PRODUCTS 12

3.1 Arylation of Naphthalene: General procedure A 12

3.2 Arylation of triphenylene: General procedure B 17

3.3 Arylation of other PAHs 22

4.0 REACTION OPTIMIZATION DATA 30

4.1 Solvent Screen 30

4.2 Naphthalene Optimization 31

4.3 Triphenylene Optimization 31

5.0 ADDITIVE SCREEN 32

6.0 SCREENING OF CATALYSTS 25

6.1 Naphthalene 25

6.2 Triphenylene 26

6.3 Discussion 26

7.0 MECHANISTIC INVESTIGATION 28

7.1 Control reactions 28

7.2 Heterogeneity Tests **28**
7.2.1 Naphthalene 28
Hg(0) poisoning test 28
Hot filtration test 29
3-Phase tests 30
7.2.2 Triphenylene 33

7.3 Kinetic experiments **33**
7.3.1 Kinetic Profile 33
7.3.2 Kinetic profile with preactivation of catalyst 34
7.3.3 $Pd(OAc)_2$ induction period 35
7.3.4 Determination of KIE 36
7.3.5 Competition experiment 37

7.4 Reaction Orders **37**
7.4.1 Order in palladium 37
7.4.2 Order in naphthalene 40

7.5 Reaction of PhI_2BF_4 in the absence of PAH **41**

7.6 Deuteration experiments **41**

8.0 FAILED OR UNSELECTIVE REACTIONS **42**

9.0 NMR SPECTRA **43**

10. 0 REFERENCES **95**

11.0 X-RAY DATA **96**

11.1 1-Phenylanthracene (6a) **96**

11.2 1-Phenyltriphenylene (5a) **97**

1.0 General Experimental

Unless otherwise noted, all reactions were carried out in oven-dried glassware under an atmosphere of air. Catalysis reactions proceed equally well in air dried glassware. Reaction temperatures are reported as the temperature of the oil bath surrounding the vessel.

Dry solvents (<50 ppm H_2O) were purchased from Acros Organics, Sigma-Aldrich or Carl Roth and stored over molecular sieves under argon atmosphere. Commercially available chemicals were obtained from Acros Organics, Sigma-Aldrich, Alfa Aesar, ABCR, TCI Europe, Combi-Blocks, Johnson-Matthey and Heraeus and used as received unless otherwise stated. The Pd/C catalysts used were of following types: Acros Organics: 10% Pd on activated carbon, unreduced; Sigma-Aldrich: 5% Pd on activated charcoal; Johnson-Matthey: 5% Pd on activated carbon paste type 39, 58.5% H_2O; 5% Pd on activated carbon paste type 58, 48.0% H_2O; 5% Pd on activated carbon paste type 452, 59.1% H_2O; 5% Pd on activated carbon paste type 478, 52.1% H_2O; **Heraeus: 5 wt% Pd/C type K-0219, <3% H_2O (standard catalyst for this study)**. Except for Pd/C from Heraeus, all catalysts were dried under vacuum over P_2O_5 (replace by fresh P_2O_5 after 4 h) for ~16 h. Dried catalysts were then stored in a glove box, for convenience rather than necessity. **NOTE:** For the catalysis reactions reported herein the catalysts could be stored and used under air.

Analytical thin layer chromatography (TLC) was performed on silica gel 60 F_{254} aluminum plates (Merck). TLC plates were visualized by exposure to short wave ultraviolet light (254 nm, 366 nm). Flash column chromatography was performed on Merck silica gel (40-63 mesh). ^{1}H-, ^{13}C- and ^{19}F-NMR spectra were recorded at room temperature on a Bruker AV 300 or AV 400 and Agilent 600 (DD2). Chemical shifts (δ) are given in ppm. The residual solvent signals were used as references and the chemical shifts converted to the TMS scale ($CDCl_3$: δ_H = 7.26 ppm, δ_C = 77.16 ppm; DMSO-d_6: δ_H = 2.50 ppm, δ_C = 39.52 ppm). ^{19}F-NMR spectra are not calibrated and δ (ppm) is given relative to CCl_3F. Coupling constants (J) are quoted in Hz. GC-MS spectra were recorded on an Agilent Technologies 7890A GC-system with an Agilent 5975C VL MSD or an Agilent 5975 inert Mass Selective Detector (EI) and a HP-5MS column (0.25 mm × 30 m, film: 0.25 µm). The major signals are quoted in *m*/*z* with the relative intensity in parentheses. The methods used start with the injection temperature T_0. After holding this temperature for 3 min, the column is heated to temperature T_1 (ramp) and this temperature is held for an additional time t (method 50_40:

T_0 = 50 °C, T_1 = 290 °C, ramp = 40 °C/min, t = 10 min). Exact ESI mass spectra were recorded on a Bruker Daltonics MicroTof. High resolution ESI mass spectra were recorded on a Thermo-Fisher Scientific Orbitrap LTQ XL. Exact EI mass spectra were recorded on a Waters-Micromass GC-Tof. Major signals are quoted in *m*/*z*. Infrared spectra were recorded neat on a Shimadzu FTIR-8400S. The wave numbers (υ) of recorded IR-signals are quoted in cm^{-1}.

2.0 Substrate Synthesis

2.1 Preparation of Iodonium Salts

Diphenyliodonium tetrafluoroborate

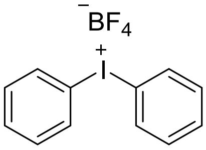

Diphenyliodonium tetrafluoroborate was synthesised according to the procedure of Bielawski *et al.*[1] Reaction of iodobenzene (5.56 g, 3.00 mL, 27.3 mmol, 1 eq) and phenylboronic acid (3.66 g, 30.0 mmol, 1.1 eq) gave the target compound (8.84 g, 24.0 mmol, 88%) as a white solid.

^{1}H-NMR (300 MHz, DMSO-d_6): δ 7.53 (t, J = 7.7 Hz, 4H, 4 × ArH), 7.70-7.63 (m, 2H, 2 × ArH), 8.25 (dd, J = 8.4, 1.2 Hz, 4H, 4 × ArH); ^{13}C-NMR (75 MHz, DMSO-d_6): δ 116.5, 131.8, 132.1, 135.2; ^{19}F-NMR (282 MHz, DMSO-d_6): δ -148.23 (m), -148.17 (bs).
Data is in accordance with the literature.[1]

Bis(2-fluorophenyl)iodonium tetrafluoroborate

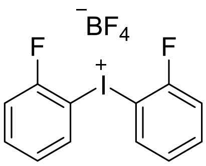

Bis(2-fluorophenyl)iodonium tetrafluoroborate was synthesised according to the procedure of Bielawski *et al.*[1] Reaction of 2-fluoro-1-iodobenzene (761 mg, 0.40 mL, 3.43 mmol, 1 eq) and 2-fluorophenylboronic acid (531 mg, 3.80 mmol, 1.1 eq) gave the target compound (1.15 g, 2.85 mmol, 83%) as a white solid.

^{1}H-NMR (300 MHz, DMSO-d_6): δ 7.38 (td, J = 7.7, 1.4 Hz, 2H, 2 × ArH), 7.53-7.65 (m, 2H, 2 × ArH), 7.67-7.79 (m, 2H, 2 × ArH), 8.36-8.48 (m, 2H, 2 × ArH); ^{13}C-NMR (75 MHz, DMSO-d_6): 104.1 (d, J = 25.2 Hz), 117.0 (d, J = 22.2 Hz), 127.8, 135.9 (d, J = 7.9 Hz), 137.1, 159.1 (d, J = 249.9 Hz); ^{19}F{^{1}H}-NMR (282 MHz, DMSO-d_6): -148.2 (m), -148.1 (bs), -97.9 (s).
Data is in accordance with the literature.[1]

Bis(4-fluorophenyl)iodonium tetrafluoroborate

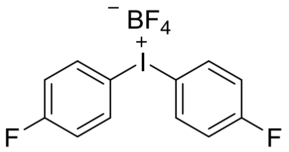

Bis(4-fluorophenyl)iodonium tetrafluoroborate was synthesised according to the procedure of Bielawski *et al.*[1] Reaction of 4-fluoro-1-iodobenzene (1.28 g, 0.67 mL, 5.75 mmol, 1 eq) and 4-fluorophenylboronic acid (885 mg, 6.32 mmol, 1.1 eq) gave the target compound (1.14 g, 2.82 mmol, 49%) as a white solid.

^{1}H-NMR (300 MHz, DMSO-d_6) δ 7.42 (d, J = 9.0 Hz, 4H, 4 × ArH), 8.32 (d, J = 9.0 Hz, 4H, 4 × ArH); ^{13}C NMR (75 MHz, DMSO-d_6) δ 111.1, 119.2 (d, J = 22.8 Hz), 137.9 (d, J = 9.1 Hz), 163.9 (d, J = 251.6 Hz); ^{19}F NMR (282 MHz, DMSO-d_6) δ -148.23 (d, J = 16.2 Hz), -106.61 (tt, J = 8.9, 5.1 Hz).

Data is in accordance with the literature.[2]

Bis(3-nitrophenyl)iodonium tetrafluoroborate

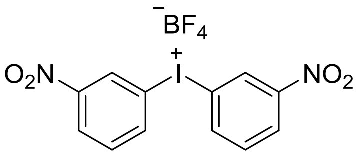

Bis(3-nitrophenyl)iodonium tetrafluoroborate was synthesised according to the procedure of Bielawski *et al.*[1] Reaction of 1-iodo-3-nitrobenzene (1.99 g, 8.00 mmol, 1 eq) and 3-nitrophenylboronic acid (1.47 g, 8.81 mmol, 1.1 eq) gave the target compound (932 mg, 2.04 mmol, 26%) as a brown solid. **Note**: 10 mL of acetonitrile were added as co-solvent after the oxidation step.

^{1}H-NMR (400 MHz, DMSO-d$_6$): δ (ppm): 7.84 (t, J = 8.2 Hz, 2H, 2 × ArH), 8.47 (d, J = 8.2 Hz, 2H, 2 × ArH), 8.74 (d, J = 8.2 Hz, 2 H, 2 × ArH), 9.29 (s, 2 H, 2 × ArH); ^{13}C-NMR (101 MHz, DMSO-d$_6$): δ (ppm): 116.9, 127.1, 130.2, 132.9, 141.4, 148.4; ^{19}F-NMR (282 MHz, DMSO-d$_6$): δ -148.24 (bs), -148.19 (bs).

Data is in accordance with the literature.[3]

Bis(2-methylphenyl)iodonium tetrafluoroborate

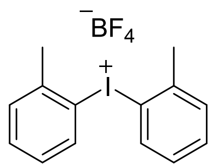

Bis(2-methylphenyl)iodonium tetrafluoroborate was synthesised according to the procedure of Bielawski *et al.*[1] Reaction of 2-iodotoluene (589 mg, 0.35 mL, 2.70 mmol, 1 eq) and 2-toluylboronic acid (407 mg, 3.00 mmol, 1.1 eq) gave the target compound (803 mg, 2.03 mmol, 75%) as a white solid.

^{1}H-NMR (300 MHz, DMSO-d_6) δ 2.61 (s, 6H, 2 × CH_3), 7.35 – 7.25 (m, 2H, 2 × ArH), 7.62 – 7.52 (m, 4H, 4 × ArH), 8.32 (d, J = 7.8 Hz, 2H, 2 × ArH). ^{13}C NMR (75 MHz, DMSO-d_6) δ 25.0, 120.6, 129.3, 131.6, 132.8, 137.2, 140.6.
Data is in accordance with the literature.[1]

Phenyl(2,4,6-triisopropylphenyl)iodonium tetrafluoroborate

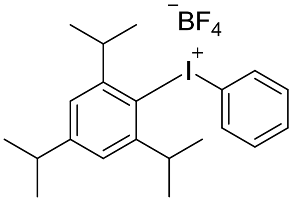

Phenyl(2,4,6-triisopropylphenyl)iodonium tetrafluoroborate was synthesised according to a modified procedure from Reisman *et al.*[4] Reaction of iodobenzene (1.12 mL, 10.00 mmol, 1 eq) and 1,3,5-triisopropylbenzene (2.67 mL, 11.00 mmol, 1.10 eq) gave the target compound (1.78 g, 3.60 mmol, 36%) as a white solid.

^{1}H NMR (300 MHz, $CDCl_3$) δ 1.26 (d, 6.8 Hz, 18H, 6 × CH_3), 2.97 (sept, *J* = 7.0 Hz, 1H, CH), 3.25 (sept, *J* = 6.7 Hz, 2H, 2 × CH), 7.19 (s, 2H, 2 × ArH), 7.44 (dd, *J* = 8.4, 6.9 Hz, 2H, 2 × ArH), 7.51 – 7.59 (m, 1H, ArH), 7.64 – 7.71 (m, 2H, 2 × ArH). ^{13}C NMR (75 MHz, $CDCl_3$) δ 23.8, 24.4, 34.3, 39.8, 112.0, 119.5, 125.6, 132.2, 132.7, 132.7, 152.7, 156.1. ^{19}F NMR (282 MHz, $CDCl_3$) δ -147.53 (bs), -147.59 (m). υ_{max} (neat)/ cm^{-1}: 741, 1011, 1041, 1084, 2966 m/z (ESI): Found $[M-BF_4]^+$, 407.1230. $C_{21}H_{28}I^+$ requires *M*, 407.1230.

(4-Methylphenyl)(2,4,6-triisopropylphenyl)iodonium tetrafluoroborate

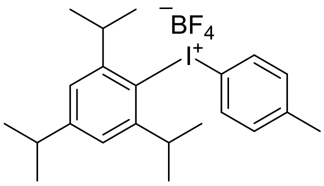

(4-Methylphenyl)(2,4,6-triisopropylphenyl)iodonium tetrafluoroborate was synthesised according to a modified procedure from Reisman *et al.*[4] Reaction of 4-iodotoluene (1,09 g, 5.00 mmol, 1 eq) and 1,3,5-triisopropylbenzene (1.34 mL, 5.50 mmol, 1.10 eq) gave the target compound (580 mg, 1.14 mmol, 24%) as a white solid.

^{1}H-NMR (300 MHz, DMSO-d_6) δ 1.21 (m, 18H, 6 × CH_3), 2.32 (s, 3H, CH_3), 2.95 (sept., J = 6.9 Hz, 1H, CH), 3.41 (sept., J = 6.7 Hz, 2H, 2 × CH), 7.30 (s, 2H, 2 × ArH), 7.34 (d, J = 8.4 Hz, 2H, 2 × ArH), 7.83 (d, J = 8.4 Hz, 2H, 2 × ArH); ^{13}C NMR (75 MHz, DMSO) δ 20.8, 23.6, 24.1, 33.4, 38.6, 111.4, 123.3, 124.6, 132.5, 134.1, 142.2, 151.1, 154.2.
^{19}F-NMR (282 MHz, DMSO-d_6): δ -148.24 (bs), -148.21 (bs); υ_{max} (neat)/ cm^{-1}: 794, 1026, 1053, 2966. m/z (ESI): Found [M-BF_4]$^+$, 421,1385. $C_{22}H_{30}I^+$ requires *M*, 421.1387.

(3-Methylphenyl)(2,4,6-triisopropylphenyl)iodonium tetrafluoroborate

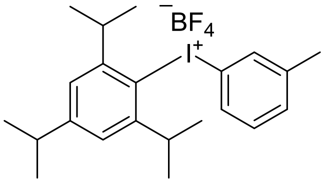

(3-Methylphenyl)(2,4,6-triisopropylphenyl)iodonium tetrafluoroborate was synthesised according to a modified procedure from Reisman *et al.*[4] Reaction of 3-iodotoluene (0.64 mL, 5.00 mmol, 1 eq) and 1,3,5-triisopropylbenzene (1.34 mL, 5.50 mmol, 1.10 eq) gave the target compound (802 mg, 1.58 mmol, 32%) as a white solid.

^{1}H NMR (300 MHz, $CDCl_3$) δ 1.36 – 1.22 (m, 18H, 6 × CH_3), 2.38 (s, 3H, CH_3), 2.98 (sept., *J* = 6.8 Hz, 1H, 1 × CH), 3.26 (sept., *J* = 6.7 Hz, 2H, 2 × CH), 7.20 (s, 2H, 2 × ArH), 7.28 – 7.39 (m, 3H, 3 × ArH), 7.60 (s, 1H, ArH),; ^{13}C NMR (75 MHz, CDCl3) δ 21.6, 23.8, 24.5, 34.3, 39.8, 111.9, 119.3, 125.6, 129.3, 132.3 133.1, 133.3, 143.7, 152.8, 156.1.
; ^{19}F-NMR (282 MHz, $CDCl_3$): δ -147.96 (bs), -147.91 (bs); υ_{max} (neat)/ cm^{-1}: 771, 1014, 1045, 2966. m/z (ESI): Found (M-BF_4)$^+$, 421.1381. $C_{22}H_{30}I^+$ requires *M*, 421.1387.

(4-Methoxyphenyl)(2,4,6-triisopropylphenyl)iodonium tetrafluoroborate

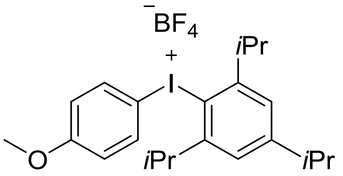

(4-Methoxyphenyl)(2,4,6-triisopropylphenyl)iodonium tetrafluoroborate was synthesised according to the procedure of Phipps *et al.*[5] Reaction of 4-methoxyphenylboronic acid (760 mg, 5.00 mmol, 1 eq) and 2-iodo-1,3,5-triisopropylbenzene (2.36 g, 5.24 mmol, 1.05 eq) gave the target compound (1.73 g, 3.30 mmol, 66%) as a white solid.

^{1}H-NMR (300 MHz, $CDCl_3$): δ 1.26 (d, J = 6.8 Hz, 6H, 2 × CH_3), 1.26 (d, J = 6.8 Hz, 12H, 4 × CH_3), 2.95 (hept, J = 6.8 Hz, 1H, CH), 3.31 (hept, J = 6.8 Hz, 2H, 2 × CH), 3.81 (s, 3H, OCH_3), 6.95 (d, J = 9.2 Hz, 2H, 2 × ArH), 7.16 (s, 2H, 2 × ArH), 7.66 (d, J = 9.2 Hz, 2H, 2 × ArH); ^{13}C-NMR (75 MHz, $CDCl_3$): δ 23.8, 24.5, 34.3, 39.7, 55.9, 99.9, 118.4, 120.5, 125.5, 135.3, 152.5, 155.8, 162.8; ^{19}F-NMR (282 MHz, $CDCl_3$): δ -148,12 (dd, J = 3.2, 1.5, Hz), -148.07 (bs).

Data is in accordance with the literature.[5]

(4-Chlorophenyl)(2,4,6-triisopropylphenyl)iodonium tetrafluoroborate

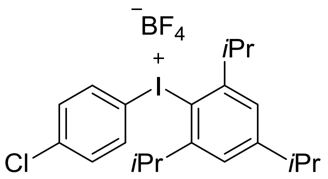

(4-Chlorophenyl)(2,4,6-triisopropylphenyl)iodonium tetrafluoroborate was synthesised according to the procedure of Phipps *et al.*[5] Reaction of 4-chloro-1-iodobenzene (2.38 g, 9.98 mmol, 1 eq) and 1,3,5-triisopropylbenzene (2.66 ml, 2.25 g, 11.0 mmol, 1.1 eq) gave the target compound (2.75 g, 5.20 mmol, 52%) as a white solid.

^{1}H-NMR (300 MHz, $CDCl_3$): δ 1.26 (d, J = 6.8 Hz, 6H, 2 × CH_3), 1.26 (d, J = 6.8 Hz, 12H, 4 × CH_3), 2.97 (hept, J = 6.8 Hz, 1H, CH), 3.26 (hept, J = 6.8 Hz, 2H, 2 × CH), 7.19 (s, 2H, 2 × ArH), 7.40 (d, J = 8.7 Hz, 2H, 2 × ArH), 7.64 (d, J = 8.7 Hz, 2H, 2 × ArH); ^{13}C-NMR (75 MHz, $CDCl_3$): δ 23.8, 24.5, 34.3, 39.8, 108.8, 120.1, 125.6, 132.7, 134.1, 139.1, 152.7, 156.1; ^{19}F-NMR (282 MHz, $CDCl_3$): δ -146.6 (dd, J = 3.4, 1.6 Hz), -146.5 (bs).

Data is in accordance with the literature.[5]

(4-(Ethoxycarbonyl)phenyl)(2,4,6-triisopropylphenyl)iodonium tetrafluoroborate

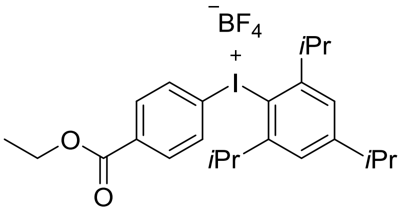

(4-(Ethoxycarbonyl)phenyl)(2,4,6-triisopropylphenyl)iodonium tetrafluoroborate was synthesised according to the procedure of Phipps *et al.*[5] Reaction of ethyl-4-iodobenzoate (2.76 g, 10 mmol, 1 eq) and 1,3,5-triisopropylbenzene (2.66 ml, 2.25 g, 11.0 mmol, 1.1 eq) gave the target compound (1.58 g, 2.79 mmol, 28%) as a white solid.

^{1}H-NMR (300 MHz, $CDCl_3$): δ 1.25 (d, J = 6.7 Hz, 12H, 4 × CH_3), 1.29 (d, J = 7.0 Hz, 6H, 2 × CH_3), 1.36 (t, J = 7.1 Hz, 3H, CH_2CH_3), 2.98 (hept, J = 7.0 Hz, 1H, CH), 3.25 (hept, J = 6.7 Hz, 2H, 2 × CH), 4.36 (q, 7.1 Hz, 2H, CH_2), 7.21 (s, 2H, 2 × ArH), 7.74 (d, J = 8.6 Hz, 2H, 2 × ArH), 8.06 (d, J = 8.6 Hz, 2H, 2 × ArH); ^{13}C-NMR (75 MHz, $CDCl_3$): δ 14.3, 23.8, 24.4, 34.4, 39.9, 62.0, 116.4, 119.8, 125.7, 132.4, 133.1, 133.9, 152.9, 156.2, 164.8; ^{19}F-NMR (282 MHz, $CDCl_3$): δ -146.54 (d, J = 3.2, 1.4 Hz), -145.48 (bs) ; υ_{max} (neat)/ cm^{-1}: 679, 725, 756, 849, 883, 976, 999, 1049, 1095, 1180, 1277, 1370, 1393, 1462, 1582, 1717, 2874, 2936, 2967; m/z (ESI): Found $[M-BF_4]^+$, 479.1428. $C_{24}H_{32}IO_2$ requires M, 479.1441.

2-(Methylphenyl)(2,4,6-triisopropylphenyl)iodonium tetrafluoroborate

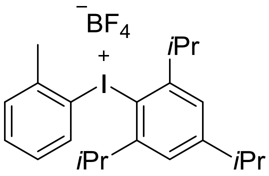

o-Tolyl(2,4,6-triisopropylphenyl)iodonium tetrafluoroborate was synthesised according to the procedure of Phipps *et al.*[5] Reaction of 2-iodo-toluene (2.18 g, 10.0 mmol, 1 eq) and 1,3,5-triisopropylbenzene (2.66 ml, 2.25 g, 11.0 mmol, 1.1 eq) gave the target compound (2.22 g, 4.37 mmol, 44%) as a white solid.

^{1}H-NMR (300 MHz, $CDCl_3$): δ 1.29 (d, J = 6.7 Hz, 12H, 4 × CH_3), 1.34 (d, J = 6.9 Hz, 6H, 2 × CH_3), 2.72 (s, 3H, CH_3), 3.04 (hept, J = 6.9 Hz, 1H, CH), 3.23 (hept, J = 6.7 Hz, 2H, 2 × CH), 7.20-7.30 (m, 3H, 3 × ArH), 7.34 (d, J = 8.3 Hz, 1H, ArH), 7.48-7.58 (m, 2H, 2 × ArH); ^{13}C-NMR (75 MHz, $CDCl_3$): δ 23.8, 24.4, 25.0, 34.3, 39.9, 116.4, 119.0, 125.8,

130.3, 132.6, 132.7, 133.0, 140.2, 152.8, 156.1.; ^{19}F-NMR (282 MHz, $CDCl_3$): δ -148.64 (d, *J* = 3.2, 1.5 Hz), -148.58 (bs) ; υ_{max} (neat)/ cm^{-1}: 644, 729, 752, 880, 980, 999, 1034, 1069, 1269, 1370, 1427, 1462, 1586, 2870, 2932, 2959; m/z (ESI): Found $[M-BF_4]^+$, 421.1369. $C_{22}H_{30}I$ requires *M*, 421.1387.

3.0 Experimental and characterization of reaction products

3.1 Arylation of Naphthalene: General procedure A

Note: Efficient stirring is crucial to obtaining reproducible yields and therefore we strongly recommend the use of cross-shaped stirring bars. Alternatively, we found that using 3 small 'standard' linear bars rather than 1 larger bar produced reproducible results. Stirring plates were set at >1000 rpm.

Unless otherwise noted, to the iodonium salt (0.500 mmol, 1.0 eq), naphthalene (2.0 eq) and Pd/C (5 wt% Pd/C, <3% H_2O, Heraeus type K-0219, 27 mg, 12.5 µmol, 2.5 mol%) was added 1,2-dimethoxyethane (5.00 mL), and the reaction stirred at the reported temperature for 16 h. The reaction mixture was allowed to cool to room temperature and then filtered through a pad of silica, washing with EtOAc (60 mL). The filtrate was concentrated in vacuo and the crude material purified by flash column chromatography.

1-Phenylnapthalene (3a)

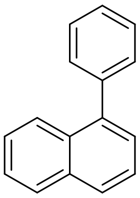

According to general procedure A: Reaction of naphthalene (**2**) (77 mg, 0.600 mmol, 2 eq) and diphenyliodonium tetrafluoroborate (110 mg, 0.300 mmol, 1 eq) in DME (3.0 mL) at 80 °C following purification using flash column chromatography eluting with 3% toluene in pentane gave **3a** (36 mg, 0.176 mmol, 59%) as a colourless oil.

R_f (3% toluene in pentane): 0.35; ^{1}H-NMR (400 MHz, $CDCl_3$): δ 7.41-7.57 (m, 9H, 9 × ArH), 7.85-7.94 (m, 3H, 3 × ArH); ^{13}C-NMR (101 MHz, $CDCl_3$): δ 125.5, 125.9, 126.15, 126.16, 127.1, 127.4, 127.8, 128.4, 130.2, 131.7, 133.9, 140.4, 140.9.
Data is in accordance with the literature.[6]

1-(2-Methylphenyl)naphthalene (3b)

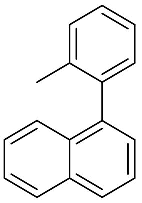

According to general procedure A: Reaction of naphthalene (**2**) (128 mg, 1.00 mmol, 2 eq) and bis(2-methylphenyl)iodonium tetrafluoroborate (198 mg, 0.500 mmol, 1 eq) at 80 °C following purification using flash column chromatography eluting with 3% toluene in pentane gave **3b** (65 mg, 0.298 mmol, 60%) as a white solid.

R_f (3% toluene in pentane): 0.48. ^{1}H NMR (300 MHz, $CDCl_3$) δ 7.85 – 7.75 (m, 2H, 2 × ArH), 7.47 – 7.35 (m, 3H, 3 × ArH), 7.33 - 7.14 (m, 6H, 6 × ArH), 1.94 (s, 3H, CH_3); ^{13}C NMR (75 MHz, $CDCl_3$) δ 140.3, 139.9, 136.9, 133.6, 132.1, 130.5, 130.0, 128.3, 127.7, 127.5, 126.7, 126.2, 126.1, 125.8, 125.7, 125.5, 20.2.
Data is in accordance with the literature.[7]

1-(2-Fluorophenyl)naphthalene (3c)

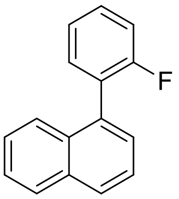

According to general procedure A: Reaction of naphthalene (**2**) (128 mg, 1.00 mmol, 2 eq) and bis(2-fluorophenyl)iodonium tetrafluoroborate (202 mg, 0.500 mmol, 1 eq) at 100 °C following purification using flash column chromatography eluting with pentane gave **3c** (57 mg, 0.256 mmol, 51%) as a colourless oil.

R_f (pentane): 0.35; ^{1}H-NMR (400 MHz, $CDCl_3$): δ 7.20-7.34 (m, 2H, 2 × ArH), 7.39-7.51 (m, 4H, 4 × ArH), 7.50-7.60 (m, 2H, 2 × ArH), 7.66-7.73 (m, 1H, ArH), 7.88-7.97 (m, 2H, 2 × ArH); ^{13}C-NMR (101 MHz, $CDCl_3$): δ 115.8 (d, J = 22.3 Hz), 124.2 (d, J = 3.7 Hz), 125.4, 125.95 (d, J = 1.4 Hz), 126.02, 126.3, 127.8, 128.1 (d, J = 16.2 Hz), 128.4, 128.5, 129.6 (d, J = 7.9 Hz), 131.9, 132.5 (d, J = 3.6 Hz), 133.7, 134.0, 160.2 (d, J = 246.7 Hz).
Data is in accordance with the literature.[8]

1-(3-Nitrophenyl)naphthalene (3d)

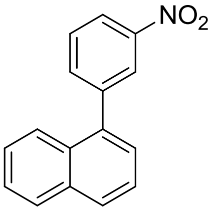

According to general procedure A: Reaction of naphthalene (**2**) (128 mg, 1.00 mmol, 2 eq) and bis(3-nitrophenyl)iodonium tetrafluoroborate (229 mg, 0.500 mmol, 1 eq) at 100 °C following purification using flash column chromatography eluting with 2% Et_2O in pentane gave **3d** (50 mg, 0.201 mmol, 40%) as a pale yellow solid.

R_f (2% Et_2O in pentane): 0.20; ^{1}H-NMR (300 MHz, $CDCl_3$): δ 7.40-7.60 (m, 4H, 4 × ArH), 7.67 (t, J = 7.9 Hz, 1H, ArH), 7.77 (dd, J = 8.2, 1.0 Hz, 1H, ArH), 7.84 (dt, J = 7.9, 1.5 Hz, 1H, ArH), 7.91-7.97 (m, 2H, 2 × ArH), 8.31 (ddd, J = 8.2, 2.3, 1.0 Hz, 1H, ArH), 8.39 (t, J = 2.0 Hz, 1H, ArH); ^{13}C-NMR (75 MHz, $CDCl_3$): δ 122.4, 125.0, 125.2, 125.5, 126.3, 126.9, 127.4, 128.7, 128.9, 129.4, 131.2, 133.9, 136.3, 137.6, 142.6, 148.5.
Data is in accordance with the literature.[9]

1-(4-Fluorophenyl)naphthalene (3e)

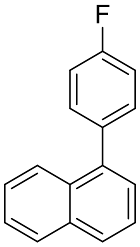

According to general procedure A: Reaction of naphthalene (**2**) (128 mg, 1.00 mmol, 2 eq) and bis(4-fluorophenyl)iodonium tetrafluoroborate (202 mg, 0.500 mmol, 1 eq) at 80 °C following purification using flash column chromatography eluting with pentane gave **3e** (59 mg, 0.266 mmol, 54%) as a white solid.
R_f (pentane): 0.38. ^{1}H-NMR (300 MHz, $CDCl_3$) δ 7.19 (t, J = 8.8 Hz, 2H, 2 × ArH), 7.38 – 7.58 (m, 6H, 6 × ArH), 7.86 (t, J = 7.5 Hz, 2H, 2 × ArH), 7.92 (dd, J = 8.2, 1.5 Hz, 1H, ArH); ^{13}C-NMR (75 MHz, $CDCl_3$) δ 115.3 (d, J = 21.3 Hz), 125.5, 125.9, 126.0, 126.3, 127.1, 127.9, 128.5, 131.5 (d, J = 7.9 Hz), 131.7, 133.9, 136.8 (d, J = 3.3 Hz), 139.3, 162.4 (d, J = 246.1 Hz); ^{19}F-NMR (282 MHz, $CDCl_3$) δ -115.5.
Data is in accordance with the literature.[6]

1-(4-Methoxyphenyl)naphthalene (3f)

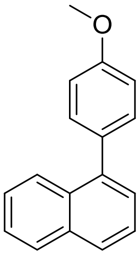

According to general procedure A: Reaction of naphthalene (**2**) (128 mg, 1.00 mmol, 2 eq) and (4-methoxyphenyl)(2,4,6-triisopropylphenyl)iodonium tetrafluoroborate (262 mg, 0.500 mmol, 1 eq) at 80 °C following purification using flash column chromatography eluting with 2% EtOAc and 2% toluene in pentane gave **3f** (64 mg, 0.273 mmol, 54%) as a white solid.

R_f (3% EtOAc in pentane): 0.33; ^{1}H-NMR (300 MHz, $CDCl_3$): δ 3.91 (s, 3H, OCH_3), 7.06 (d, J = 8.6 Hz, 2H, 2 × ArH), 7.38-7.59 (m, 6 H, 6 × ArH), 7.86 (d, J = 8.2 Hz, 1H, ArH), 7.88-8.01 (m, 2H, 2 × ArH); ^{13}C-NMR (75 MHz, $CDCl_3$): δ 55.5, 113.8, 125.5, 125.8, 126.1, 126.2, 127.0, 127.5, 128.4, 131.2, 131.9, 133.2, 134.0, 140.0, 159.1.
Data is in accordance with the literature.[10]

1-(3-Methylphenyl)naphthalene (3g)

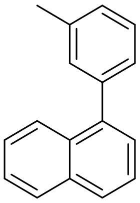

According to general procedure A: Reaction of naphthalene (2) (128 mg, 1.00 mmol, 2 eq) and (3-Methylphenyl)(2,4,6-triisopropylphenyl)iodonium tetrafluoroborate (254 mg, 0.500 mmol, 1 eq) at 80 °C following purification using flash column chromatography eluting with pentane:toluene (97:3) gave **3g** (44 mg, 0.201 mmol, 40%) as a colorless oil.

R_f (3% toluene in pentane): 0.50. ^{1}H-NMR (300 MHz, $CDCl_3$) δ 2.39 (s, 3H, CH_3), 7.24 – 7.17 (m, 1H, ArH), 7.29 – 7.50 (m, 7H, 7 × ArH), 7.80 (d, J = 8.2 Hz, 1H, ArH), 7.85 (ddd, J = 7.8, 3.8, 1.4 Hz, 2H, 2 × ArH). ^{13}C-NMR (75 MHz, CDCl3) δ 21.7, 125.5, 125.8, 126.1, 126.2, 127.0, 127.3, 127.6, 128.1, 128.2, 128.4, 130.9, 131.8, 133.9, 138.0, 140.5, 140.8.
Data is in accordance with the literature.[7]

1-(4-Methylphenyl)naphthalene (3h)

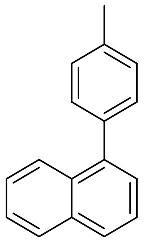

According to general procedure A: Reaction of naphthalene (**2**) (51,2 mg, 0,40 mmol, 2 eq) and (4-methylphenyl)(2,4,6-triisopropylphenyl)iodonium tetrafluoroborate (102 mg, 0.200 mmol, 1 eq) at 100 °C following purification using flash column chromatography eluting with pentane gave **3h** (20,6 mg, 0.094 mmol, 48%) as a colorless oil.

R_f (pentane): 0.39. ^{1}H NMR (400 MHz, $CDCl_3$) δ 2.49 (s, 3H, CH_3), 7.33 (d, *J* = 7.8 Hz, 2H, 2 × ArH), 7.40 – 7.48 (m, 4H, 4 × ArH), 7.48 – 7.58 (m, 2H, 2 × ArH), 7.87 (dt, *J* = 8.2, 1.1 Hz, 1H, ArH), 7.94 (ddd, *J* = 12.2, 8.3, 1.3 Hz, 2H, 2 × ArH). ^{13}C NMR (101 MHz, $CDCl_3$) δ 21.4, 125.5, 125.8, 126.0, 126.2, 126.9, 127.0, 127.6, 128.4, 129.1, 129.6, 130.1, 131.8, 133.9, 137.0, 137.9, 140.4.
Data is in accordance with the literature.[7]

Methyl 4-(naphthalene-1-yl)benzoate (3i)

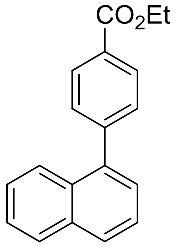

According to general procedure A: Reaction of naphthalene (**2**) (128 mg, 1.00 mmol, 2 eq) and (4-(ethoxycarbonyl)phenyl)(2,4,6-triisopropylphenyl)iodonium tetrafluoroborate (283 mg, 0.500 mmol, 1 eq) at 100 °C following purification using flash column chromatography eluting with 2% EtOAc in pentane gave **3i** (62 mg, 0.224 mmol, 45%) as a white solid.
R_f (2% EtOAc in pentane): 0.19; ^{1}H-NMR (400 MHz, $CDCl_3$): *δ* 1.45 (t, *J* = 7.1 Hz, 3H, CH_3), 4.45 (q, *J* = 7.1 Hz, 2H, CH_2), 7.41-7.61 (m, 6H, 6 × ArH), 7.82-7.96 (m, 3 H, 3 × ArH), 8.19 (d, *J* = 8.4 Hz, 2H, 2 × ArH); ^{13}C-NMR (101 MHz, $CDCl_3$): 14.5, 61.2, 125.5, 125.8, 126.1, 126.5, 127.1, 128.4, 128.5, 129.5, 129.7, 130.2, 131.4, 133.9, 139.3, 145.6, 166.7.

Data is in accordance with the literature.[11]

1-(4-Chlorophenyl)naphthalene (3j)

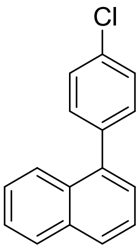

According to general procedure A: Reaction of naphthalene (**2**) (128 mg, 1.00 mmol, 2 eq) and (4-chlorophenyl)(2,4,6-triisopropylphenyl)iodonium tetrafluoroborate (264 mg, 0.500 mmol, 1 eq) at 100 °C following purification using flash column chromatography eluting with pentane gave **3j** (48 mg, 0.201 mmol, 40%) as a white solid.

R_f (pentane): 0.50; ^{1}H-NMR (400 MHz, $CDCl_3$): δ 7.38-7.58 (m, 8H, 8 × ArH), 7.88 (d, J = 8.4 Hz, 1H, ArH), 7.90 (d, J = 8.2 Hz, 1H, ArH), 7.94 (d, J = 8.0 Hz, 1H, ArH); ^{13}C-NMR (101 MHz, $CDCl_3$): 125.5, 125.8, 126.0, 126.4, 127.1, 128.1, 128.5, 128.6, 131.47, 131.54, 133.4, 133.9, 139.0, 139.3.
Data is in accordance with the literature.[6]

3.2 Arylation of triphenylene: General procedure B

To the iodonium salt (0.400 mmol, 1.0 eq), triphenylene (182 mg, 0.800 mmol, 2.0 eq) and Pd/C (5 wt% Pd/C, <3% H_2O, Heraeus type K-0219, 42 mg, 5 mol%) was added 1,2-dimethoxyethane (2.00 mL), and the reaction stirred at 100 °C for 24 h. The reaction mixture was allowed to cool to room temperature and then filtered through a pad of silica, washing with EtOAc (60 mL). The filtrate was concentrated in vacuo and the crude material purified by flash column chromatography.

1-Phenyltriphenylene (5a)

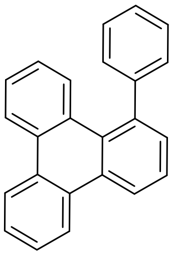

Reaction of triphenylene (182 mg, 0.800 mmol, 2 eq) and diphenyliodonium tetrafluoroborate (147 mg, 0.400 mmol, 1 eq) following purification using flash column chromatography eluting with 3% toluene in pentane gave **5a** (79 mg, 0.26 mmol, 65%) as a white solid.

R_f (pentane): 0.14; ^{1}H-NMR (300 MHz, $CDCl_3$): δ 7.06 (ddd, J = 8.4 Hz, J = 7.0 Hz, 1.4 Hz, 1H, ArH), 7.36-7.49 (m, 6H, 6 × ArH), 7.53 (dd, J = 7.3 Hz, J = 1.4 Hz, 1 H, ArH), 7.62-7.70 (m, 3H, 3 × ArH), 7.73 (dd, J = 8.5 Hz, J = 1.2 Hz, 1H, Ar-H), 8.55 (d, J = 8.0 Hz, 1H, ArH), 8.58-8.68 (m, 3H, 3 × ArH); ^{13}C-NMR (101 MHz, $CDCl_3$): 122.4, 123.2, 123.3, 123.8, 125.1, 126.5, 126.7, 127.0, 127.45, 127.47, 128.8, 129.1, 129.3, 129.8, 130.1, 130.2, 130.4, 131.1, 131.5, 131.7, 140.8, 145.5; υ_{max} (neat)/ cm^{-1}: 748, 768, 810, 1400, 1431, 1493, 1955, 3024, 3051, 3075; GC-MS (EI): t_R (50_40): 14.7 min; m/z (%): 305 (21), 304 (99), 303 (100), 302 (65), 300 (25), 289 (20); m/z (APCI): Found (M), 304,1242. $C_{24}H_{16}$ requires M, 304,1252.

1-(4-Bromophenyl)triphenylene (5b)

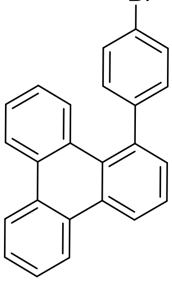

Reaction of triphenylene (182 mg, 0.800 mmol, 2 eq) and bis(4-bromophenyl)iodonium tetrafluoroborate (211 mg, 0.400 mmol, 1 eq) following purification using flash column chromatography eluting with 2% toluene in pentane gave **5b** (89 mg, 0.232 mmol, 58%) as a white solid.

R_f (pentane): 0.26; ^{1}H NMR (300 MHz, $CDCl_3$) δ 7.13 (ddd, *J* = 8.4, 7.0, 1.4 Hz, 1H, ArH), 7.26 – 7.34 (m, 2H, 2 × ArH), 7.43 – 7.52 (m, 2H, 2 × ArH), 7.52 – 7.60 (m, 2H, 2 × ArH), 7.59 – 7.77 (m, 4H, 4 × ArH), 8.46 – 8.70 (m, 4H, 4 × ArH); ^{13}C NMR (75 MHz, $CDCl_3$) δ 121.2, 122.7, 123.3, 123.4, 123.8, 125.3, 126.6, 126.9, 127.5, 127.6, 128.6, 129.4, 129.9, 130.1, 130.3, 130.9, 131.2, 131.4, 131.8, 132.3, 139.4, 144.4; υ_{max} (neat)/ cm^{-1}:725, 748, 806, 1003, 1261, 1431, 1489; GC-MS was uninformative; m/z (APCI): Found (M), 382,0350. $C_{24}H_{15}Br$ requires *M*, 382,0357.

1-(3-Nitrophenyl)triphenylene (5c)

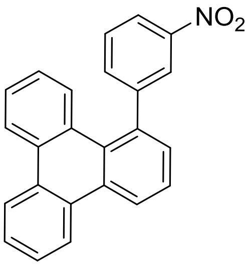

Reaction of triphenylene (137 mg, 0.600 mmol, 2 eq) and bis(3-nitrophenyl)iodonium tetrafluoroborate (137 mg, 0.300 mmol, 1 eq) with Pd/C (32 mg, 5 mol%) following purification using flash column chromatography eluting with 2% EtOAc in pentane gave **5c** (42 mg, 0.120 mmol, 40%) as a yellow solid.

R*f* (5% EtOAc in pentane): 0.4; ^{1}H NMR (400 MHz, $CDCl_3$) δ 8.71 – 8.60 (m, 3H, 3 × ArH), 8.57 (dd, *J* = 8.3, 1.3 Hz, 1H, ArH), 8.37 (t, *J* = 2.0, 2.0 Hz, 1H, ArH), 8.25 (ddd, *J* = 8.3, 2.3, 1.1 Hz, 1H, ArH), 7.76 – 7.63 (m, 4H, 4 × ArH), 7.61 – 7.42 (m, 4H, 4 × ArH), 7.06 (ddd, *J* = 8.4, 7.0, 1.4 Hz, 1H, ArH). ^{13}C NMR (101 MHz, $CDCl_3$) δ 149.0, 147.1, 138.0, 135.8, 132.1, 131.5, 131.4, 130.4, 130.1, 129.9, 129.8, 128.9, 128.6, 127.8, 127.7, 127.2, 126.7, 125.3, 123.9, 123.8, 123.7, 123.4, 123.3, 122.0. υ_{max} (neat)/ cm^{-1} 725, 748, 810, 1003, 1068, 1264, 1338, 1431, 1489, 1523; m/z (ESI): Found (M + Na), 372.0995. $C_{24}H_{15}NNaO_2$ requires *M*, 372.1000.

1-(*o*-Tolyl)triphenylene (5d)

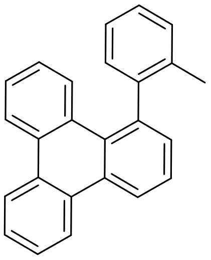

Reaction of triphenylene (137 mg, 0.600 mmol, 2 eq) and bis(2-methylphenyl)iodonium tetrafluoroborate (119 mg, 0.300 mmol, 1 eq) with Pd/C (32 mg, 5 mol%) following purification using flash column chromatography eluting with 2% EtOAc in pentane gave **5d** (43 mg, 0.135 mmol, 45%) as a colourless amorphous solid.

R*f* (2% toluene in pentane): 0.2; ^{1}H NMR (300 MHz, $CDCl_3$) δ 8.79 – 8.30 (m, 4H, 4 × ArH), 7.65 (dd, *J* = 8.5, 1.3 Hz, 1H, ArH), 7.57 (ddd, *J* = 7.1, 5.4, 3.0 Hz, 3H, 3 × ArH), 7.39 (ddd, *J* = 8.2, 7.0, 1.3 Hz, 1H, ArH), 7.33 (dd, *J* = 7.3, 1.4 Hz, 1H, ArH), 7.29 – 7.07 (m, 4H, 4 × ArH), 6.98 (ddd, *J* = 8.4, 7.0, 1.4 Hz, 1H, ArH), 1.79 (s, 3H, CH_3);^{13}C NMR (75 MHz, $CDCl_3$) δ 145.3, 140.0, 135.5, 131.5, 131.2, 130.7, 130.4, 130.3, 130.3, 129.6, 129.2, 129.1, 128.4, 127.8, 127.5, 127.4, 127.4, 126.8, 126.5, 125.9, 123.8, 123.3, 123.1, 122.6, 20.2. $\upsilon_{\max}$ (neat)/ cm-1: 729, 748, 837, 1219, 1431, 1508, 1600, 3047, 3067. m/z (APCI): Found (M + H), 319,1476. $C_{25}H_{19}$ requires *M*, 319,1487.

1-(4-Fluorophenyl)triphenylene (5e)

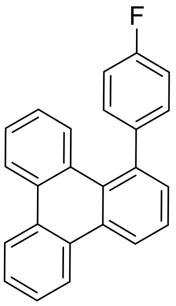

Reaction of triphenylene (182 mg, 0.800 mmol, 2 eq) and bis(4-fluorophenyl)iodonium tetrafluoroborate (162 mg, 0.400 mmol, 1 eq) at 100 °C following purification using flash column chromatography eluting with a gradient of 0-5% toluene in pentane gave **5e** (81 mg, 0.251 mmol, 63%) as a white solid.

R_f (Pentane): 0.12. ^{1}H NMR (300 MHz, $CDCl_3$) δ = 7.07 – 7.18 (m, 3H), 7.34 – 7.42 (m, 2H), 7.5 (ddd, *J*=8.2, 5.8, 1.4 Hz, 2H), 7.60 – 7.74 (m, 4H), 8.6 (dd, *J*=8.2, 1.3 Hz, 1H), 8.6 (ddd,

J=8.8, 5.3, 2.4 Hz, 3H); ^{13}C NMR (75 MHz, $CDCl_3$) δ 163.86 , 160.60 , 141.40 (d, J = 3.5 Hz), 139.67 , 131.50 (t, J = 22.4 Hz), 130.79 (d, J = 7.8 Hz), 130.55 – 129.45 (m), 128.76 , 127.54 (d, J = 3.2 Hz), 126.83 , 126.51 , 125.18 , 123.77 , 123.31 (d, J = 6.1 Hz), 122.54 , 116.09 (d, J = 21.3 Hz). ^{19}F NMR (282 MHz, $CDCl_3$) δ -115.75. . υ_{max} (neat)/ cm-1: 729, 748, 837, 1219, 1431, 1508, 1600, 3047, 3067. m/z (APCI): Found (M + H), 321,1071. $C_{24}H_{14}F$ requires M, 321,1074.

Ethyl 3-(triphenylen-1-yl)benzoate (5f)

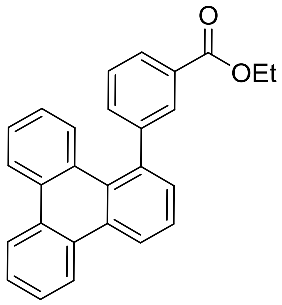

Reaction of triphenylene (182 mg, 0.800 mmol, 2 eq) and bis(3-(ethoxycarbonyl)phenyl)iodonium tetrafluoroborate (205 mg, 0.400 mmol, 1 eq) following purification using flash column chromatography eluting with 2% EtOAc in pentane gave **5f** (77 mg, 0.204 mmol, 51%) as a colourless amorphous solid.

Rf (2% EtOAc in pentane): 0.25; ^{1}H NMR (300 MHz, $CDCl_3$) δ 8.69 – 8.59 (m, 3H, 3 × ArH), 8.58 – 8.51 (m, 1H, ArH), 8.24 (td, J = 1.8, 1.7, 0.6 Hz, 1H, ArH), 8.10 (dt, J = 7.4, 1.6, 1.6 Hz, 1H, ArH), 7.74 – 7.58 (m, 4H, 4 × ArH), 7.58 – 7.35 (m, 4H, 4 × ArH), 7.06 (ddd, J = 8.3, 7.0, 1.3 Hz, 1H, ArH), 4.41 (q, J = 7.1, 7.1, 7.1 Hz, 2H, 2 × ArH), 1.41 (t, J = 7.1, 7.1 Hz, 3H, 3 × ArH). ^{13}C NMR (75 MHz, $CDCl_3$) δ 166.7, 145.7, 139.7, 134.1, 131.8, 131.6, 131.6, 131.2, 130.3, 130.1, 130.0, 129.9, 129.5, 129.0, 128.7, 128.2, 127.6, 127.5, 126.9, 126.5, 125.2, 123.8, 123.4, 123.3, 122.8, 61.2, 14.5. υ_{max} (neat)/ cm^{-1}: 725, 748, 810, 1003, 1091, 1238, 1431, 1489, 1713; m/z (APCI): Found (M + Na), 399,1355. $C_{27}H_{20}NaO_2$ requires M, 399,1361.

1-(4-Chlorophenyl)triphenylene (5g)

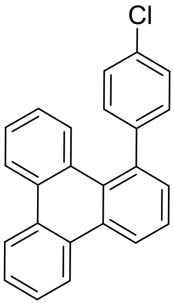

Reaction of triphenylene (182 mg, 0.800 mmol, 2 eq) and (4-chlorophenyl)(2,4,6-triisopropylphenyl)iodonium tetrafluoroborate (211 mg, 0.400 mmol, 1 eq) following purification using flash column chromatography eluting with 2% EtOAc in pentane gave **5g** (87 mg, 0.256 mmol, 64%) as a white solid.

R*f* (6% toluene in pentane): 0.38; ^{1}H NMR (300 MHz, $CDCl_3$) δ 8.75 – 8.47 (m, 4H, 4 × ArH), 7.83 – 7.57 (m, 4H, 4 × ArH), 7.53 – 7.45 (m, 2H, 2 × ArH), 7.44 – 7.32 (m, 4H, 4 × ArH), 7.12 (ddd, *J* = 8.4, 7.0, 1.4 Hz, 1H, ArH). ^{13}C NMR (75 MHz, $CDCl_3$) δ 143.9, 139.4, 133.1, 131.8, 131.4, 131.2, 130.6, 130.3, 130.1, 130.0, 129.5, 129.3, 128.7, 127.6, 127.5, 126.9, 126.5, 125.3, 123.8, 123.4, 123.3, 122.7. υ_{max} (neat)/cm^{-1} 725, 748, 772, 810, 1066, 1084, 1435, 1489; m/z (APCI): Found (M), 338,0855. $C_{24}H_{14}Cl$ requires *M*, 338,0862.

3.3 Arylation of other PAHs

1-Phenylanthracene (6a)

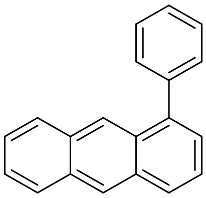

According to general procedure A: Reaction of anthracene (445 mg, 2.50 mmol, 5 eq) and diphenyliodonium tetrafluoroborate (184 mg, 0.500 mmol, 1 eq) at 80 °C following purification using flash column chromatography eluting with 3% toluene in pentane gave **6a** (69 mg, 0.271 mmol, 54%) as a pale yellow solid.

R_f (3% toluene in pentane): 0.30; ^{1}H-NMR (400 MHz, $CDCl_3$): δ 7.40-7.65 (m, 9H, 9 × ArH), 7.91 (d, J = 8.4 Hz, 1H, ArH), 8.04 (dd, J = 8.6, 4.4 Hz, 2H, 2 × ArH), 8.51 (d, J = 3.8 Hz, 2H, 2 × ArH); ^{13}C-NMR (101 MHz, $CDCl_3$): 125.1, 125.1, 125.4, 125.7, 126.3, 126.7, 127.5, 128.0, 128.1, 128.5, 128.7, 130.3, 130.4, 131.6, 131.9, 132.2, 140.4, 141.1; υ_{max} (neat)/ cm^{-1}: 702, 729, 799, 849, 883, 956, 1026, 1072, 1091, 1165, 1261, 1447, 1489, 1539, 2851, 2924, 2963, 3055; GC-MS (EI): t_R (50_40): 10.8 min; m/z (%): 255 (20), 254 (100), 253 (74), 252 (50); m/z (ESI): Found (M + Ag), 361.0140. $C_{20}H_{14}Ag$ requires M, 361.0141.

1-(4-Chlorophenyl)anthracene (6b)

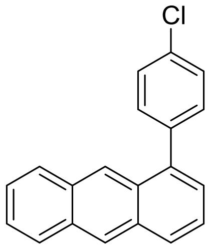

According to general procedure A: Reaction of anthracene (445 mg, 2.50 mmol, 5 eq) and (4-chlorophenyl)(2,4,6-triisopropylphenyl)iodonium tetrafluoroborate (264 mg, 0.500 mmol, 1 eq) at 100 °C following purification by flash column chromatography (× 2) eluting with pentane gave **6b** (71 mg, 0.246 mmol, 49%) as a pale yellow solid.

R_f (pentane): 0.35. ^{1}H NMR (400 MHz, CDCl3) δ 7.38 (dd, J = 6.8, 1.2 Hz, 1H, ArH), 7.42 – 7.60 (m, 7H, 7 × ArH), 7.90 (d, J = 8.2 Hz, 1H, ArH), 7.98 – 8.09 (m, 2H, 2 × ArH), 8.42 (d, J = 13.1 Hz, 1H, ArH), 8.50 (s, 1H, ArH). ^{13}C-NMR (75 MHz, $CDCl_3$) δ 139.4, 139.0, 133.6, 132.1, 131.9, 131.6, 131.5, 130.2, 128.8, 128.7, 128.4, 128.0, 126.8, 126.4, 125.9, 125.6, 124.9, 124.8. υ_{max} (neat)/ cm^{-1}: 736, 887, 1485, 3051. m/z (ESI): Found (M), 288.0700. $C_{20}H_{13}Cl$ requires M, 288.0700.

Ethyl 3-(anthracen-1-yl)benzoate (6c)

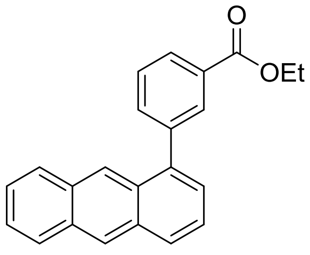

According to general procedure B: Reaction of anthracene (148 mg, 0.800 mmol, 2 eq) and bis(3-(ethoxycarbonyl)phenyl)iodonium tetrafluoroborate (205 mg, 0.400 mmol, 1 eq) at

100 °C following purification by flash column chromatography eluting with 2% EtOAc in pentane gave **6c** (57 mg, 0.176 mmol, 44%) as a yellow oil.

R_f (2% EtOAc:pentane): 0.33. ^{1}H NMR (400 MHz, CDCl3) δ ^{1}H NMR (300 MHz, $CDCl_3$) δ 1.42 (t, *J* = 7.1 Hz, 3H, CH_3), 4.44 (q, *J* = 7.1 Hz, 2H, CH_2), 7.40 – 7.59 (m, 4H, 4 × ArH), 7.64 (t, *J* = 7.6 Hz, 1H, ArH), 7.79 (dt, *J* = 7.6, 1.4 Hz, 1H, ArH), 7.90 (d, *J* = 8.2 Hz, 1H, ArH), 8.05 (t, *J* = 9.0 Hz, 2H, ArH), 8.21 (dt, *J* = 7.8, 1.4 Hz, 1H, ArH), 8.30 (t, *J* = 1.5 Hz, 1H, ArH), 8.37 (s, 1H, ArH), 8.52 (s, 1H, ArH). ^{13}C NMR (75 MHz, $CDCl_3$) δ 14.5 , 61.2 , 124.8 , 124.9 , 125.6 , 125.8 , 126.5 , 126.8 , 128.0 , 128.5 , 128.5 , 128.7 , 128.7 , 130.2 , 131.0 , 131.2 , 131.6 , 131.9 , 132.1 , 134.6 , 139.3 , 141.3 , 166.7 . υ_{max} (neat)/ cm^{-1}: 648, 879, 1107, 1211, 1249, 1292, 1712. m/z (ESI): Found (M), 327.1380. $C_{23}H_{19}O_2$ requires *M*, 327,1385.

4-Phenylpyrene (7a)

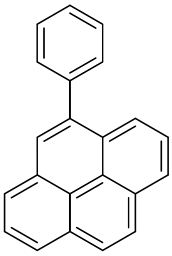

According to general procedure A: Reaction of pyrene (202 mg, 1.00 mmol, 2 eq) and bis(3-(ethoxycarbonyl)phenyl)iodonium tetrafluoroborate (205 mg, 0.400 mmol, 1 eq) at 100 °C following purification using flash column chromatography eluting with pentane gave **7a** (101 mg, 0.363 mmol, 72%) as a white solid.

R_f (pentane): 0.24; ^{1}H-NMR (300 MHz, $CDCl_3$): δ 7.49-7.65 (m, 3H, 3 × ArH), 7.71 (dd, *J* = 8.1, 1.5 Hz, 2H, 2 × ArH), 7.98 (t, *J* = 7.8 Hz, 1H, ArH), 8.05 (t, *J* = 7.7 Hz, 1H, ArH), 8.06 (s, 1H, ArH), 8.13 (s, 2H, 2 × ArH), 8.19-8.27 (m, 4H, 4 × ArH); ^{13}C-NMR (75 MHz, $CDCl_3$): 124.0, 124.3, 125.0, 125.1, 125.2, 125.3, 125.9, 126.2, 127.4, 127.7, 127.8, 127.9, 128.6, 130.2, 130.6, 130.9, 131.2, 131.5, 139.7, 141.0.
Data is in accordance with the literature.[12]

4-(2-Methylphenyl)pyrene (7b)

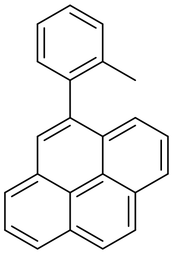

According to general procedure A: Reaction of pyrene (202 mg, 1.00 mmol, 2 eq) and bis(2-methylphenyl)iodonium tetrafluoroborate (198 mg, 0.500 mmol, 1 eq) at 80 °C following purification using flash column chromatography eluting with pentane gave **7b** (97 mg, 0.332 mmol, 66%) as a white solid.

R_f (pentane): 0.25; ^{1}H-NMR (300 MHz, $CDCl_3$): □ 2.16 (s, 3H, CH_3), 7.37-7.51 (m, 4H, 4 × ArH), 7.81 (dd, J = 7.1, 1.1 Hz, 1H, ArH), 7.94 (t, J = 7.7 Hz, 1H, ArH), 7.99 (s, 1H, ArH), 8.06 (t, J = 7.6 Hz, 1H, ArH), 8.14 (s, 2H, 2 × ArH), 8.19-8.27 (m, 3H, 3 × ArH); ^{13}C-NMR(75 MHz,$CDCl_3$): δ 20.3, 124.0, 124.4, 124.9, 125.0, 125.2, 125.2 125.9, 126.0, 126.2, 127.4, 127.6, 127.7, 128.0, 130.2, 130.8, 131.0, 131.2, 131.5, 137.2, 139.3, 140.4. Data is in accordance with the literature.[12]

4-(4-Chlorophenyl)pyrene (7c)

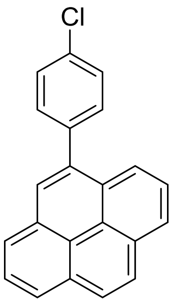

According to general procedure A: Reaction of pyrene (202 mg, 1.00 mmol, 2 eq) and (4-chlorophenyl)(2,4,6-triisopropylphenyl)iodonium tetrafluoroborate (264 mg, 0.500 mmol, 1 eq) at 100 °C following purification using flash column chromatography eluting with pentane gave **7c** (92 mg, 0.294 mmol, 59%) as a white solid.

R_f (pentane): 0.26; ^{1}H-NMR (400 MHz, $CDCl_3$): 7.55 (d, J = 8.4 Hz, 2H, 2 × ArH), 7.61 (d, J = 8.4 Hz, 2H, 2 × ArH), 7.96 (t, J = 7.8 Hz, 1H, ArH), 7.89 (s, 1H, ArH), 8.04 (t, J = 7.6 Hz, 1H, ArH), 8.25-8.10 (m, 6H, 6 × ArH); ^{13}C-NMR(100 MHz, $CDCl_3$): δ 123.7,

124.4, 125.1, 125.2, 125.3, 125.5, 125.9, 126.3, 127.5, 127.8, 128.0, 128.8, 130.3, 130.7, 131.2, 131.5, 133.7, 138.2, 139.4.
Data is in accordance with the literature.[12]

9-Phenylphenanthrene (8)

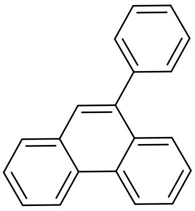

According to general procedure A: Reaction of phenanthrene (178 mg, 1.00 mmol, 2 eq) and diphenyliodonium tetrafluoroborate (184 mg, 0.500 mmol, 1 eq) at 70 °C following purification using flash column chromatography eluting with pentane gave a mixture (90:6:4, as determined using GC-FID) of three regioisomers (73 mg, 0.287 mmol, 57% total yield) as a white solid. 9-phenylphenanthrene **8** is the major isomer (65 mg, 0.256 mmol, 51% yield).

R_f (pentane): 0.28; ^{1}H-NMR (300 MHz, $CDCl_3$): 7.44-7.71 (m, 9H, 9 × ArH), 7.72 (s, 1H, ArH), 7.90-7.95 (d, *J* = 8.4 Hz, 1H, ArH), 7.96 (dd, *J* = 8.3 Hz, *J* = 1.2 Hz, 1H, ArH), 8.75 (d, *J* = 8.4 Hz, 1H, ArH), 8.81 (d, *J* = 8.3 Hz, 1H, ArH); ^{13}C-NMR (75 MHz, $CDCl_3$): 122.7, 123.0, 126.58, 126.63, 126.7, 127.0, 127.1, 127.5, 127.6, 128.4, 128.8, 130.1, 130.2, 130.8, 131.3, 131.7, 138.9, 140.9.
Data is in accordance with the literature.[13]

5-Phenylacenapthene (9a)

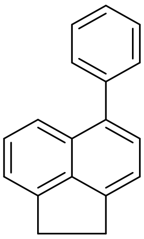

According to general procedure A: Reaction of acenaphthene (154 mg, 1.00 mmol, 2 eq) and diphenyliodonium tetrafluoroborate (184 mg, 0.500 mmol, 1 eq) at 70 °C following purification using flash column chromatography eluting with pentane gave **9a** (62 mg, 0.269 mmol, 54%) as a white solid.

R_f (pentane): 0.30; ^{1}H-NMR (300 MHz, $CDCl_3$): δ 3.47 (s, 4H, 2 × CH_2), 7.34 (d, *J* = 6.8 Hz, 1H, ArH), 7.37 (d, *J* = 7.1 Hz, 1H, ArH), 7.40-7.56 (m, 5H, 5 × ArH), 7.60 (dd, *J* = 8.2 Hz, 1.4 Hz, 2H, 2 × ArH), 7.74 (d, *J* = 8.3 Hz, 1H, ArH); ^{13}C-NMR (75 MHz, $CDCl_3$): 30.2, 30.7, 119.2, 119.5, 121.0, 127.1, 128.1, 128.5, 128.7, 129.9, 135.8, 139.7, 140.6, 145.7, 146.3.

Data is in accordance with the literature.[14]

Ethyl 3-(1,2-dihydroacenaphthylen-5-yl)benzoate (9b)

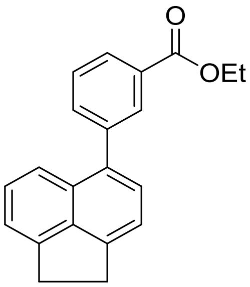

According to general procedure B: Reaction of acenaphthene (123 mg, 0.800 mmol, 2 eq) and bis(3-(ethoxycarbonyl)phenyl)iodonium tetrafluoroborate (205 mg, 0.400 mmol, 1 eq) at 100 °C following purification using flash column chromatography eluting with 2% EtOAc in pentane gave **9b** (53 mg, 0.176 mmol, 44%) as a yellow oil.

R_f (2% EtOAc:pentane): 0.23. ^{1}H NMR (300 MHz, $CDCl_3$) δ 1.41 (t, *J* = 7.1 Hz, 3H, CH_3), 3.46 (s, 4H, 2 × CH_2), 4.41 (q, *J* = 7.1 Hz, 2H, CH2), 7.30 – 7.40 (m, 2H, 2 × ArH), 7.40 – 7.50 (m, 2H, 2 × ArH), 7.53 – 7.69 (m, 2H, 2 × ArH), 7.76 (dt, *J* = 7.6, 1.5 Hz, 1H, ArH), 8.10 (dt, *J* = 7.8, 1.5 Hz, 1H, ArH), 8.26 (t, *J* = 1.8 Hz, 1H, ArH). ^{13}C NMR (75 MHz, $CDCl_3$) δ 14.5 , 30.2 , 30.7 , 61.2 , 119.2 , 119.6 , 120.6 , 128.3 , 128.4 , 128.5 , 128.9 , 129.7 , 130.9 , 130.9 , 134.3 , 134.7 , 139.7 , 140.8 , 146.2 , 146.4 , 166.8 . υ_{max} (neat)/ cm^{-1}: 644, 1030, 1084, 1107, 1320, 1249, 1300, 1716; m/z (ESI): Found [M+H], 303,1385. $C_{21}H_{19}O_2$ requires *M*, 303,1385.

5-(4-Bromophenyl)acenapthene (9c)

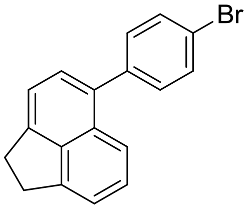

According to general procedure A: Reaction of acenaphthene (154 mg, 1.00 mmol, 2 eq) and bis(4-bromophenyl)iodonium tetrafluoroborate (263 mg, 0.500 mmol, 1 eq) at 100 °C following purification using flash column chromatography eluting with pentane gave **9c** (71 mg, 0.231 mmol, 47%) as a white solid.

R_f (pentane): 0.26; ^{1}H NMR (400 MHz, $CDCl_3$) δ 3.39 – 3.52 (bm, 4H, 2 x CH_2), 7.34 (ddd, *J* = 6.6, 3.9, 1.3 Hz, 2H, 2 x ArH), 7.39 (d, *J* = 7.1 Hz, 1H, 1 x ArH), 7.42 – 7.48 (m, 3H, 3 x ArH), 7.59 – 7.69 (m, 3H, ArH); ^{13}C NMR (101 MHz, $CDCl_3$) δ 30.18, 30.67, 119.24, 119.66, 120.59, 121.24, 128.39, 128.62, 129.59, 131.51, 131.53, 131.62, 134.40, 139.45, 139.67, 146.19, 146.43; m/z (APCI): Found (M) 308.0194, $C_{18}H_{13}Br$ requires *M*, 308.0195. υ_{max} (neat)/ cm^{-1}: 3063, 2940, 1605, 1485, 1069, 1007, 818, 779.

3-Phenylfluoranthene (10a)

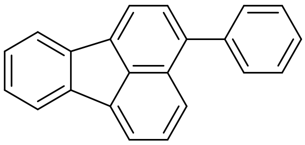

According to general procedure A: Reaction of fluoranthene (202 mg, 1.00 mmol, 2 eq) and diphenyliodonium tetrafluoroborate (184 mg, 0.500 mmol, 1 eq) at 80 °C following purification using flash column chromatography eluting with pentane gave a mixture (95:5, as determined using GC-FID) of two regioisomers (94 mg, 0.338 mmol, 68% total yield) as a yellow solid. 3-Phenylfluoranthene **10** is the major isomer (89 mg, 0.320 mmol, 64% yield).

R_f (pentane): 0.20; ^{1}H-NMR (400 MHz, $CDCl_3$): 7.39-7.44 (m, 2H, 2 × ArH), 7.48 (td, *J* = 7.3 Hz, 1.4 Hz, 1H, ArH), 7.55 (t, *J* = 7.4 Hz, 2H, 2 × ArH), 7.60-7.67 (m, 4H, 4 × ArH), 7.93-8.02 (m, 5H, 5 × ArH); ^{13}C-NMR (101 MHz, $CDCl_3$): 120.2, 121.56, 121.63, 125.8, 127.5, 127.6, 127.7, 128.2, 128.5, 128.8, 130.4, 132.8, 136.4, 137.2, 139.3, 139.7, 139.9, 140.4.

Data is in accordance with the literature.[15]

3-(4-Fluorophenyl)fluoranthene (10b)

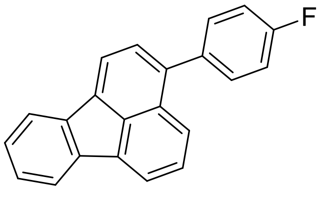

According to general procedure A: Reaction of fluoranthene (202 mg, 1.00 mmol, 2 eq) and bis(4-fluorophenyl)iodonium tetrafluoroborate (202 mg, 0.500 mmol, 1 eq) at 80 °C following purification using flash column chromatography eluting with pentane gave a mixture (97:3, as determined using GC-FID) of two regioisomers (70 mg, 0.236 mmol, 47% total yield) as a yellow solid. 3-(4-Fluorophenyl)fluoranthene **10** is the major isomer (68 mg, 0.223 mmol, 45 % yield).

4.0 Reaction Optimization Data

4.1 Solvent Screen

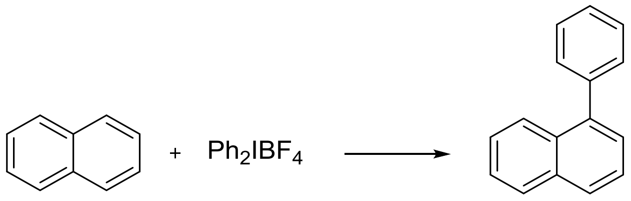

Reactions were performed exactly as described for the substrate scope.

**Solvent**	**Yield %**[a]	α:β[a]
EtOH	Trace	nd
DME	46	93:7
THF	16	88:12
$CHCl_3$	23	80:20
MeCN	2	nd
Heptane	4	nd
EtOAc	10	89:11
Trifluoroethanol	14	nd
$PhNO_2$	3	nd
DME:Dioxane 1:1	45	nd
DME:Dioxane 9:1	46	nd
DME:Dioxane 1:9	20	nd
Triglyme	40	nd
DME:Triglyme 1:1	43	nd
NMP	0	nd
DMF	0	nd
DMSO	0	nd

Conditions: Naphthalene (0.1 mmol), Ph_2IBF_4 (1.4 eq), Pd/C 5 mol%, solvent (0.5 mL) 70 °C, 22 h. [a]Determined by GC-FID. nd is not determined.

4.2 Naphthalene Optimization

Deviation from standard conditions	Yield %[a]	α:β[a]
Equivalents of Ph_2IBF_4		
0.5	56[b]	92:8[c]
1.0	43	95:5[c]
1.2	46	94:6[c]
1.4	46	93:7[c]
Other		
2.5 mol% Pd/C, 22 h	62	93:7
2.5 mol% Pd/C, 16 h	62	92:7
2.5 mol% Pd/C, 16 h, 1mL DME	63	95:5

Standard conditions: Naphthalene (0.1 mmol), Ph_2IBF_4 (0.5 eq), Pd/C 5 mol%, DME (0.5 mL), 80 °C, 22 h. [a]Determined by GC-FID. [b]Based on Ph_2IBF_4 nd is not determined. [c] 70 °C.

4.3 Triphenylene Optimization

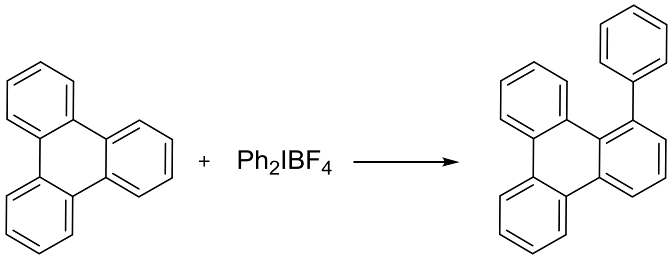

Reactions were performed exactly as described for the substrate scope.

Deviation from standard conditions	Yield %[a]
None	40[b]
Temperature	
100 °C	49
Equivalents of PhI_2BF_4	
1	23
2	43
Catalyst loading	
5 mol% Pd/C	60
10 mol% Pd/C	61
Concentration	
[0.2]	67
[0.05]	63
Reaction Time	
24 h	65
30 h	68
5 mol% Pd/C, [0.2], 24 h	65[b]

Standard conditions: Triphenylene (0.1 mmol), Ph_2IBF_4 (2 eq), Pd/C 2.5 mol%, DME (1.0 mL), 80 °C, 16 h. [a]Determined by GC-FID. [b]Isolated yield

5.0 Additive Screen

We extensively screened additives in an attempt to increase both the yield and selectivity of reactions. In each case we observed either no effect or a detrimental effect on the reaction.

Additives screened:

Organic Additives
TMEDA
DMEDA
18-Crown-6
Ethylene Glycol
Ethyldiamine
DMS
PhSSPh
Tetramethylthiourea

Acetates
LiOAc
NaOAc
KaOAc
CsOAc
AgOAc

Salts
LiCl
NaCl
KCl
LiBr
NaBr
KBr
CuCl
$CuCl_2$
$ZnCl_2$
$MgCl_2$
CuBr
$MgBr_2$
CuCl

Oxidants
$Cu(OAc)_2$
$Cu(OAc)_2$. H_2O
KS_2O_8
Benzoquinone
AgOAc
Chloroanil
DDQ
PIDA
Oxone
tBuOOH
Ditbutylperoxide

Acids
Acetic acid
TFA
TsOH
iPrCOOH
PivOH
Proline
Acetic acid
TFA
TsOH
iPrCOOH
PivOH
Proline

Amine Ligands
Pyridine
2,6-Dimethylpyrazine
Bipyridine
Proline
Phenanthroline
Pyridine
Pyrazine
Bipyridine
Proline
Phenanthroline

Lewis-acids
TMSOTf
BF_3-OEt_2
TMSCl

Ammonium Salts
Bu_4NCl
Bu_4NBr
Bu_4NI

Bases
Cs_2CO_3
CsOPiv
F_3CCO_2Ag
Li_2CO_3
K_2CO_3
Cs_2CO_3
CsOPiv
Li_2CO_3
DBU
DIPEA
Et_3N
2,6-Tertbuylpyridine

Phosphines
PPh_3
$P(Cy)_3$
$P(OEt)_3$
dppp
dppe
PPh_3

6.0 Screening of Catalysts

6.1 Naphthalene

**Catalyst**	**Yield %**[a]	**α:β**[a]
Pd/C (Heraeus)	63	95:5
**'Homogeneous' Catalysts**[c]		
$Pd(dba)_3$	23	94:6
K_2PdCl_4	8	95:5
$PdCl_2$(dppe)	3	82:18
$PdCl_2(PPh_3)_3$	9	96:4
$Pd(PPh_3)_4$	43	95:5
$Pd(OAc)_2$	52	93:7
**Different Pd/C sources**		
Sigma-Aldrich	49	93:7
Acros Organics	7	96:4
Johnson-Matthey Type 39	15	95:5
Johnson-Matthey Type 58	46	94:6
Johnson-Matthey Type 452	57	94:6
Johnson-Matthey Type 478	33	96:4
**Supported Pd catalysts**		
$Pd(OH)_2$/C	2	nd
Pd/Fe_3O_4	42	88:12
Pd/Rh/C (4:1)	1	nd
Pd/Pt/C (4:1)	5	nd
$Pd(OH)_2/Fe_3O_4$	12	96:4
Pd/Al_2O_3	64	96:4
**Supported metals**		
Pt/C	0	nd
Pt/Fe_3O_4	1	nd
$Ir/CaCO_3$	2	nd
Ir/C	2	nd
Rh/C	1	nd
Ru/C	0	nd
Ru/Fe_3O_4	1	nd
$Ru(OH)_3/Fe_3O_4$	0	nd
$Ni(OH)_2/Fe_3O_4$	2	nd
$Cu(OH)_2/Fe_3O_4$	0	nd
$Co(OH)_2/Fe_3O_4$	1	nd

Standard conditions: Naphthalene (0.2 mmol), Ph_2IBF_4 (0.5 eq), Catalyst 2.5 mol%, DME (1 mL), 80 °C, 16 h. [a]Determined by GC-FID [b]Based on Ph_2IBF_4. [c]5 mol% catalyst. nd is not determined.

6.2 Triphenylene

Catalyst	Yield %[a]
Pd/C (Heraeus)	65[b]
**'Homogeneous' Catalysts**	
$Pd(dba)_3$	23
$PdCl_2$	16
$Pd(PPh_3)_4$	37
$Pd(OAc)_2$	43
**Different Pd/C sources**	
Acros Organics	57
Johnson-Matthey Type 39	49
Johnson-Matthey Type 58	67
**Supported Pd catalysts**	
$Pd(OH)_2/C$	19
Pd/Fe_3O_4	60
Pd/Al_2O_3	72

Standard conditions: Triphenylene (0.30 mmol), Ph_2IBF_4 (0.5 eq), Catalyst 5 mol%, DME (0.5 mL), 100 °C, 24 h. [a]Determined by GC-FID [b]Isolated yield.

6.3 Discussion

The catalyst screen demonstrated that Pd/C (Hereaus) and Pd/Al_2O_3 are equally proficient catalysts. As in our previous studies, it is also very clear that the source of Pd/C has an impact on yield. While homogeneous Pd(0) catalysts also show reactivity, Pd(II) catalysts (homogeneous or heterogeneous) are ineffective. Comparative studies of Pd/C, Pd/Al_2O_3 and $Pd(OAc)_2$ provided some insight to the different reactivity of these systems.

Catalyst	Hg poisoning	Hot filtration test	Standard reaction	5 equiv. **1**	Reaction with [*p*MeOPhI(TRIP)]BF_4	Reaction with [*o*-$MePh_2I$]BF_4	+Radical inhibitor[b]
Pd/C (Heraeus)	het	het	63%	75%	54%[a]	60%[a]	0%
Pd/Al_2O_3	het	het	64%	79%	trace	trace	0%
$Pd(OAc)_2$[c]	het	het	52%	50%	30%	nd	0%

Standard conditions: Naphthalene (**1**) (2 equiv.), iodonium salt (1 equiv.), catalyst (2.5 mol%), DME (0.1 M), 80 °C, 16 h. Yields given are GC-yields for the major regioisomer unless noted otherwise. *het* indicates that the results suggest a heterogeneous active catalytic species. [a]isolated yield. [b]TEMPO (1 equiv.), 1,4-benzoquinone (1 equiv.) were evaluated. [c]5 mol% of catalyst used.

Experiments were indicative of a heterogeneous catalytic species for each Pd source. A radical component to the reaction mechanism in each case was also apparent. While Pd/Al_2O_3 showed excellent reactivity in the standard reaction, no product formation was observed with

electron rich iodonium salts. Interestingly, this is consistent with a Pd(II)/(IV) manifold as proposed by Sanford. ‘Poisoning’ reactions of electron-rich iodonium salts using Pd/C as catalyst by the addition of Pd/Al_2O_3 did not occur, suggesting that the composition of the support alone does not account for the inhibition of the reaction. $Pd(OAc)_2$ generally proved less efficient that Pd/C, which may be accounted for by the reduced stability of the nanoparticles in the absence of a support.

7.0 Mechanistic Investigation

Studies were undertaken on both naphthalene and triphenylene in a number of instances, to confirm the same mechanism is in operation. If not considered essential, mechanistic studies were only undertaken on one substrate. More extensive studies were typically undertaken employing naphthalene for economic considerations.

7.1 Control reactions

**Deviation from standard conditions**	**Yield %**[a,b]	**α:β**[a]
None	63	95:5
No catalyst	0	nd
Degassed solvent, Ar atmosphere	63	95:5

Standard conditions: Naphthalene (0.2 mmol), Ph_2IBF_4 (0.5 eq), Catalyst 2.5 mol%, DME (1 mL), 80 °C, 16 h. [a]Determined by GC-FID. [b]Based on Ph_2IBF_4.

Experiments demonstrate catalyst is essential for reactivity and rule out air as an oxidant.

7.2 Heterogeneity Tests

7.2.1 Naphthalene

Hg(0) poisoning test

Pd/C (2.5 mol %), Pd/Al_2O_3 (2.5 mol %) and $Pd(OAc)_2$ (5 mol %) were all investigated.

As per general procedure A, two reactions of naphthalene (0.20 mmol, 2 eq.) (**1**) and $[Ph_2I]BF_4$ (0.10 mmol) with the addition of mesitylene (20 µL) as an internal standard were prepared, one as a control. Following reaction for 4.5 h at 80 °C the yields of both reactions were then determined by GC-FID. Elemental mercury (160 mg) was introduced to one reaction at 80 °C. Both reaction mixtures were continued with stirring at 80 °C for 22 h. Subsequent analysis of the reaction yields show the reaction is inhibited by the introduction of Hg(0).

Catalyst	Pd/C		Pd/Al_2O_3		$Pd(OAc)_2$	
	Yield (4,5 h)[a]	Yield (22 h)	Yield (4,5 h)	Yield (22 h)	Yield (4,5 h)	Yield (22 h)
Control reaction	19 %	51 %	4 %	67 %	35 %	51 %
Hg(0) poisoning test	17 %	17 %	3 %	3 %	34 %	36 %

[a]Yields determined by GC of crude reaction mixture, with mesitylene as internal standard.

Hot filtration test

Pd/C (2.5 mol %), Pd/Al_2O_3 (2.5 mol %) and $Pd(OAc)_2$ (5 mol %) were all investigated.

As per general procedure A, reaction of naphthalene (0.20 mmol, 2 eq.) (**1**) and $[Ph_2I]BF_4$ (0.10 mmol) for 4 h at 80 °C was undertaken. The reaction was then directly filtered through a preheated (80 °C) pad of Celite into a fresh vessel containing $[Ph_2I]BF_4$ (0.10 mmol, 1 eq). The reaction mixture was then analysed using GC prior to heating for an additional 22 h at 80 °C. Subsequent analysis showed no reaction progress following filtration.

Catalyst	Pd/C		Pd/Al_2O_3		$Pd(OAc)_2$	
	Yield (4,5 h)[a]	Yield (22 h)	Yield (4,5 h)	Yield (22 h)	Yield (4,5 h)	Yield (22 h)
Control reaction	15 %	58 %	4 %	67 %	35 %	51 %
Hot filtration test	20 %	20 %	4 %	4 %	26 %	27 %

[a]Yields determined by GC of crude reaction mixture, with mesitylene as internal standard.

The hot filtration test is a widely applied method to investigate the nature of the active catalytic species. Following filtration of the reaction mixture to remove any heterogeneous catalyst, should reaction continue this would be indicative of leached (homogeneous) active catalytic species.

3-Phase tests

Preparation of compounds

2-(Naphthalen-2-yl)ethanol

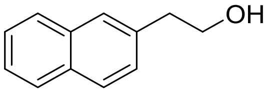

To a solution of 2-vinylnaphthalene (1.00 g, 6.50 mmol, 1 eq) in THF (5 mL) at 0 °C, was added BH_3-THF (1 M, 3.25 mL, 3.25 mmol, 0.50 eq) slowly over 5 min. The solution was stirred at room temperature for 45 min. After this time, the reaction was cooled to 0 °C and NaOH (3 M, 2 mL) was added, followed by the slowly addition of H_2O_2 (30 % solution, 2 mL). The resulting mixture was heated at reflux for 1 h. The mixture was poured into cold water and extracted with ethyl acetate (3 × 15 mL). The organic phase was washed with HCl 1 M (30 mL), dried with $MgSO_4$ and concentrated *in vacuo*. Purification with SiO_2 gel gave the target compound (516 mg, 3.00 mmol, 46%) as a white solid.

R_f (40 % Ethyl acetate in pentane): 0.30. ^{1}H NMR (400 MHz, $CDCl_3$) δ 1.59 (br. s., 1H, OH), 3.04 (t, *J* = 6.5 Hz, 2H, CH_2), 3.95 (t, *J* = 6.5 Hz, 2H, CH_2OH), 7.37 (dd, *J* = 8.4, 1.8 Hz, 1H, 1 × ArH), 7.42 – 7.51 (m, 2H, 2 × ArH), 7.69 (s, 1H, ArH), 7.82 (ddd, *J* = 10.3, 6.2, 2.8 Hz, 3H, 3 × ArH); ^{13}C-NMR (101 MHz, $CDCl_3$) δ 136.1, 133.7, 132.4, 128.4, 127.8, 127.6, 127.6, 127.5, 126.2, 125.6, 63.7, 39.5. Data is in accordance with the literature.[16]

2-(2-Naphthyl)ethyl tosylate

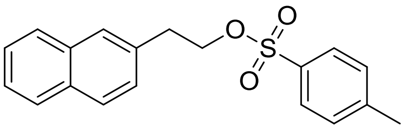

2-(2-Naphthyl)ethyl tosylate was synthesized according to the procedure of Glorius *et al*.[17] Reaction of 2-(naphthalen-2-yl)ethanol (510 mg, 2.96 mmol, 1 eq), tosyl chloride (677 mg, 3.55 mmol, 1.2 eq) and triethylamine (0.66 mL, 4.74 mmol, 1.6 eq) gave the target compound (725 mg, 2.22 mmol, 76%) as a colorless oil.

R_f (40 % Ethyl acetate in pentane): 0.65. ^{1}H NMR (400 MHz, $CDCl_3$) δ 2.34 (s, 3H, CH_3), 3.11 (t, *J* = 6.8 Hz, 2H, CH_2), 4.31 (t, *J* = 6.8 Hz, 2H, CH_2), 7.11 (d, *J* = 8.0 Hz, 2H, 2 × ArH), 7.21 (dd, *J* = 8.5, 1.7 Hz, 1H, ArH), 7.50 – 7.43 (m, 2H, 2 × ArH), 7.52 (d, *J* = 1.6 Hz, 1H, ArH), 7.60 (d, *J* = 8.0 Hz, 2H, 2 × ArH), 7.71 (d, *J* = 8.5 Hz, 2H, 2 × ArH), 7.84 – 7.77 (m,

1H, ArH); ^{13}C NMR (101 MHz, $CDCl_3$) δ 21.7, 35.6, 70.8, 125.8, 126.3, 127.2, 127.6, 127.6, 127.7, 127.9, 128.4, 129.8, 132.5, 132.9, 133.6, 133.9, 144.7. GC-MS (EI): t_R (50_40): 12.5 min; *m*/*z* (%): 326, 154, 141, 115, 91.

Inmobilization of 2-(2-naphthyl)ethyl tosylate on Wang Resin

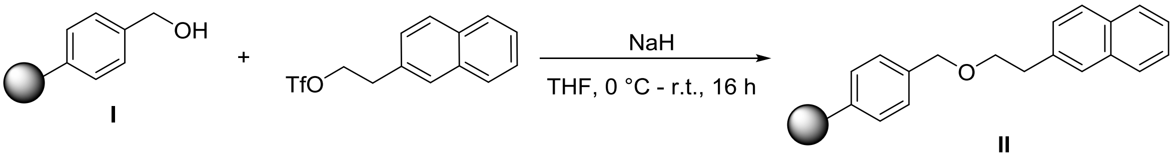

Commercially available Wang-resin (100-200 mesh, 1.0-1.5 mmol/g OH loading, 1% crosslinked with divinylbenzene) was washed with CH_2Cl_2 and MeOH and dried *in vacuo* overnight prior to use. To a suspension of Wang-resin I (500 mg, 0.750 mmol, 1.0 eq) in THF (2 mL) at 0 °C was added NaH (57 wt% in mineral oil, 93 mg, 2.2 mmol, 2.9 eq) and the mixture stirred for 2 h. Then, 2-(2-naphthyl)ethyl tosylate (621 mg, 2.20 mmol, 2.9 eq) dissolved in THF (2 mL), was added dropwise and the reaction mixture was stirred at room temperature for 16 h. The reaction mixture was quenched with H_2O (5 mL), filtered and the residue consecutively washed with CH_2Cl_2, MeOH, H_2O, MeOH and CH_2Cl_2 (30 mL each). The resin was dried in vacuo to afford Wang-resin II.

To determine the loading, 50 mg of Wang-resin II was stirred in TFA/CH_2Cl_2 3:1 (8 mL) at room temperature for 2 h. The reaction mixture was filtered, the residue washed with CH_2Cl_2 (20 mL) and the filtrate washed with saturated aqueous $NaHCO_3$ (2x 30 mL), dried over $MgSO_4$ and the solvent removed *in vacuo*. The mass of the isolated material allowed for determination of the loading of the Wang resin. The material was analysed by GCMS and gave a loading of 0.45 mmol/g.

3-Phase tests

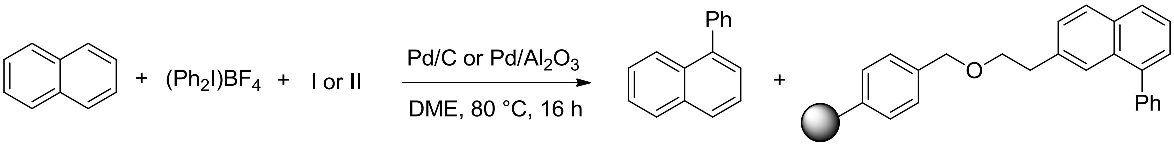

Reactions were undertaken as per general procedure A, substituting naphthalene (**1**) for Wang-resin (**II**). To determine whether the immobilized substrate had reacted under the given

reaction conditions, the reaction residue was washed, then treated with TFA/ CH_2Cl_2 (3:1) as reported (vide supra). Products were analysed by GCMS.

To address different scenarios of possible catalyst leaching, several variants of 3-phase tests have been under taken:

a) *Pd/C (2,5 % mol) as catalyst*

1. The standard reaction was undertaken in the presence of unmodified Wang-resin (I, 200 mg) to evaluate possible reaction inhibition.
Result: The reaction proceeded normally.

2. Supported naphthalene (**II**) was used in place of naphthalene and the reaction evaluated as normal.
Result: No cleavage of naphthalene was detected. No product detected in solution or in the solid support.
Experiment 2 strongly suggests that no active homogenous Pd species are generated under these reaction conditions. It also demonstrates that the supported naphthalene is stable to the reaction conditions.
In order to confirm the reactivity of alkyl naphthalene derivatives, 2-ethylnaphthalene was employed under the standard reaction conditions and the expected arylated product was obtained.

3. The standard reaction of **1** and $[Ph_2I]BF_4$ was undertaken in the presence of polymer supported naphthalene (**II**). **Result:** Reaction product was observed in solution but not on solid support.
This experiment demonstrates that the active catalyst species is present in solution as reaction is observed. The absence of product formation on the support strongly suggests that the active species is heterogeneous in nature. This experiment is performed to rule out a synergistic effect of naphthalene and the aryliodonium salt being required to generate an active homogeneous catalytic species.

b) *Pd/Al_2O_3 (2,5 mol %) as catalyst*

Reactions identical to those described for Pd/C were undertaken, and were again strongly indicative of the active species being heterogeneous in nature.

7.2.2 Triphenylene

Hg(0) poisoning test

As per general procedure B, triphenylene (**4**) (0.150 mmol) and $[Ph_2I]BF_4$ with the addition of mesitylene (14 μL) as an internal standard was prepared. Following reaction for 1 h at 100 °C the yield was determined by GC-FID. Elemental mercury (80 mg) was introduced to the reaction at ~100 °C and the reaction then stirred at 100 °C for an additional 22 h. Subsequent analysis of the reaction yield showed the reaction is inhibited by the introduction of Hg(0).

Hot filtration test

As per general procedure B, triphenylene (**4**) (0.150 mmol) and $[Ph_2I]BF_4$ with the addition of mesitylene (14 μL) as an internal standard was prepared. Following reaction for 1 h at 100 °C the reaction was then directly filtered through a preheated (80 °C) pad of Celite into a fresh vessel containing $[Ph_2I]BF_4$ (1 eq). The reaction mixture was then analysed using GC prior to heating for an additional 22 h at 100 °C. Subsequent analysis showed no reaction progress following filtration.

	**Yield %[a] 1h**	**Yield %[a] 23h**
Hg poisoning test	24	24
Hot filtration test	22	21

[a]Yields determined by GC of crude reaction mixture, with mesitylene as internal standard.

7.3 Kinetic experiments

7.3.1 Kinetic Profile

Reactions performed as per general procedure A. Each time point is a separate experiment and results are the average of two experiments.

Reaction time h	Yield %
2	2
4	7
6	23
8	41
10	52
12	58
14	62
20	62

[a]Yields determined by GC of crude reaction mixture, with mesitylene as internal standard.

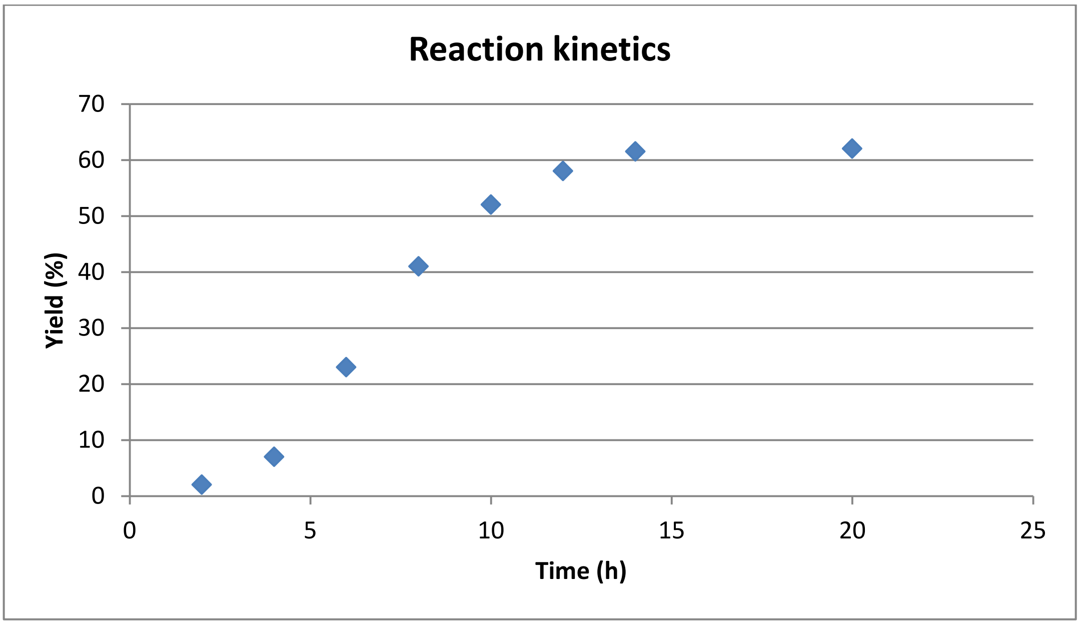

7.3.2 Kinetic profile with preactivation of catalyst

As per general procedure A: PhI_2BF_4 (0.100 mmol, 1 equiv) and Pd/C (2.5 % mol) in DME (1.0 mL) were heated at 80 °C for 2h. Naphthalene (2 equiv) was then added to the reaction mixture and stirring at 80 °C continued. Aliquots were taken to determine the yield of reaction. Results are the average of 3 different experiments.

Reaction time h	Yield %
0.5	4
1	12
1.5	18
2	23
3	30
4	33
5	35
6	40
9	18
22	52

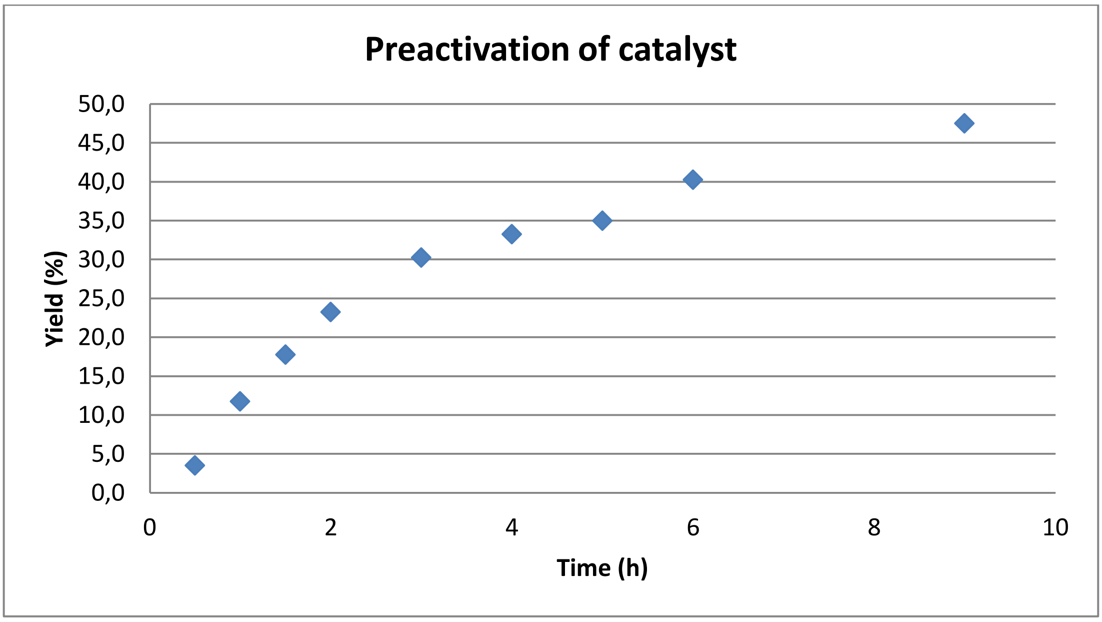

Preheating the catalyst in the absence of an oxidant for 2 h prior to the introduction of the reagents did not negate the induction period. After the addition of the reagents, the yield of the reaction was determined to be 4% after 4 h. This is consistent with the kinetic profile of the standard reaction.

7.3.3 $Pd(OAc)_2$ induction period

According to general procedure A: Reaction of naphthalene (**2**) (26 mg, 0.200 mmol, 2 eq) and diphenyliodonium tetrafluoroborate (37 mg, 0.100 mmol, 1 eq) with $Pd(OAc)_2$ (1 mg, 5 mol%) at 80 °C was undertaken. Aliquots were taken hourly and yields determined by GC-FID.

Reaction time h	Yield %
1	0
2	5
3	6
4	8
5	13
6	17

[a]Yields determined by GC of crude reaction mixture, with mesitylene as internal standard.

7.3.4 Determination of KIE

Via initial rate constants:

As per general procedure A, reaction of naphthalene (**1**) (0.200 mmol, 2.0 eq) or naphthalene-d_8 (d-**1**) (0.200 mmol, 2.0 equiv.), with $[Ph_2I]BF_4$ (37 mg, 0.100 mmol, 1.0 equiv.) was undertaken.

The KIE was determined for the reaction time between 2 h and 4 h. The yields were determined by GC using mesitylene as internal standard. The initial rate constants were calculated:

k_H = 6.40 mg·mL·h^{-1}

k_D = 11.80 mg·mL·h^{-1}

k_H/k_D = 0.54

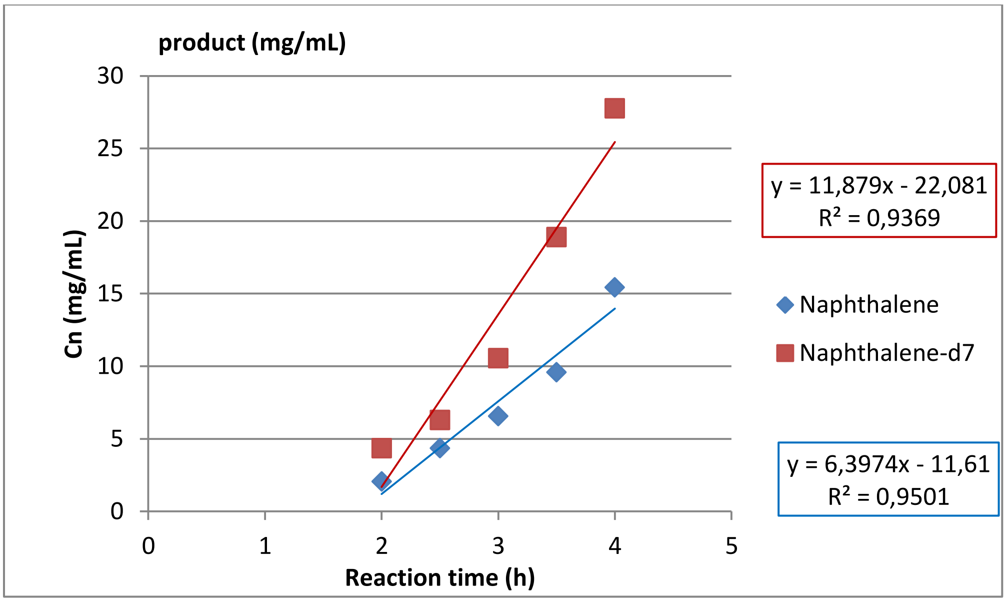

Initial rates of standard reaction with naphthalene (blue) and naphthalene-d_8 (red)

7.3.5 Competition experiment

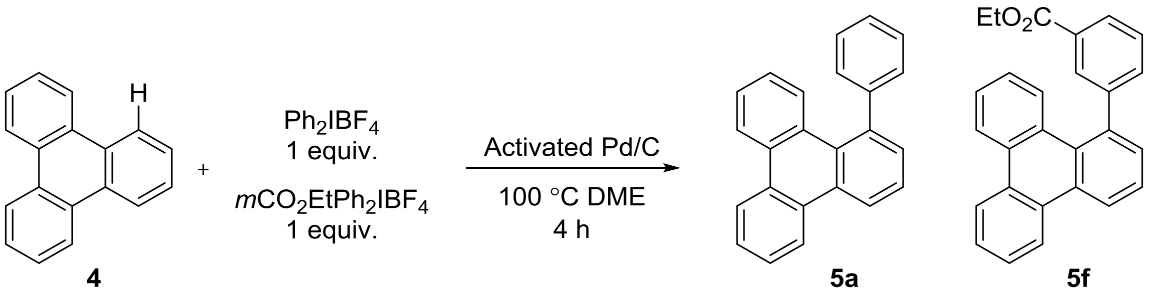

To ensure formation of the active catalyst did not consume any reagent and affect the results, Pd/C (21 mg, 5 mol%) was pre-treated with PIDA (13 mg, 20 mol%) at 100 °C in DME (0.5 ml) for 2 h to form the active catalytic species. To the active catalyst solution was then added triphenylene (**4**) (46 mg, 0.200 mmol, 1 eq) PhI_2BF_4 (74 mg, 0.200 mmol, 1 eq) and *m*-$CO_2EtPh_2IBF_4$ (102 mg, 0.200 mmol, 1 eq) and the reaction stirred for 4 h. Yields were determined at 2 h and 4h using GC-FID and indicated reaction of PhI_2BF_4 is more rapid than *m*-$CO_2EtPh_2IBF_4$.

Product	Yield %[a] 2h	Yield %[a] 3h
**5a**	8	19
**5d**	3	10

[a]Yields determined by GC of crude reaction mixture, mesitylene as internal standard.

7.4 Reaction Orders

7.4.1 Order in palladium

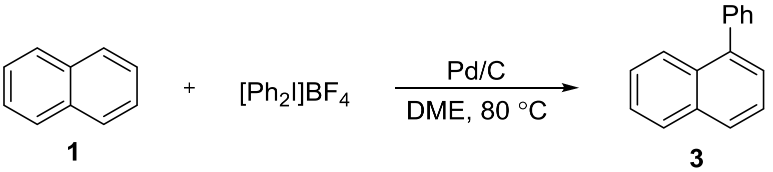

Reactions were undertaken as per general procedure A. Catalyst loadings of 2.5 mg, 5 mg, 10 mg and 20 mg were undertaken. The induction period was established to be dependent on catalyst concentration, hence preliminary experiments were undertaken to establish the duration of the induction period and hence establish when measurement should begin. Initial reaction rates were determined by measuring the rate of product formation between ~3 and ~12 % yield, with a minimum of 5 data points. Plotting the initial rates (gradient) vs. catalyst

amount showed a linear correlation indicative of 1st order reaction kinetics. Surprisingly, no product formation was observed after 16 h with 2.5 mg of catalyst.

	**Yield (3a) %[a]**		
**Pd/C loading**	**5 mg**	**10 mg**	**20 mg**
Time min			
90			3.4
105			5.1
120	3.3	4.0	7.0
135		5.5	12.5
150	5.9	6.9	14.5
165		9.1	
180	7.7	11.1	
210	9.8		
240	12.0		

[a]Yields determined by GC of crude reaction mixture, with mesitylene as internal standard.

	**mg/mL (3a)**		
**Pd/C loading**	**5 mg**	**10 mg**	**20 mg**
Time min			
90			0.8
105			1.2
120	0.8	1.0	1.7
135		1.3	3.0
150	1.4	1.7	3.5
165		2.2	
180	1.9	2.7	
210	2.4		
240	2.9		

Plot of Concentration (2a) vs Time

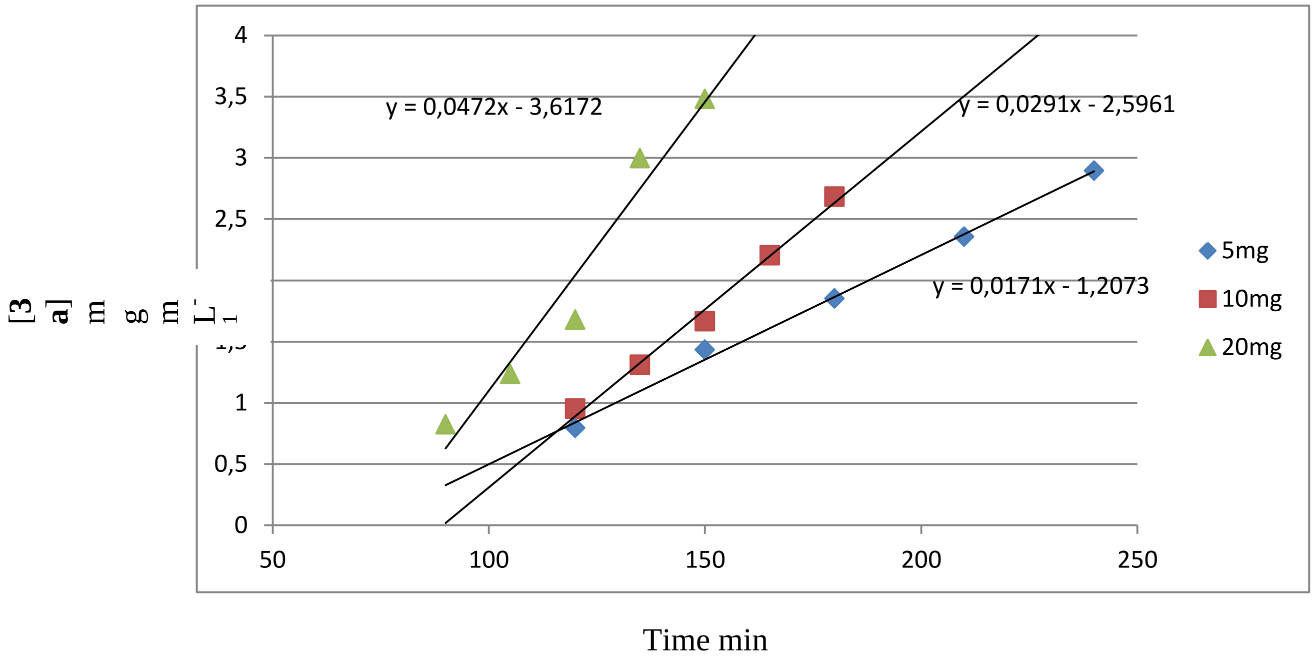

Plot of Initial Rate vs Catalyst Loading

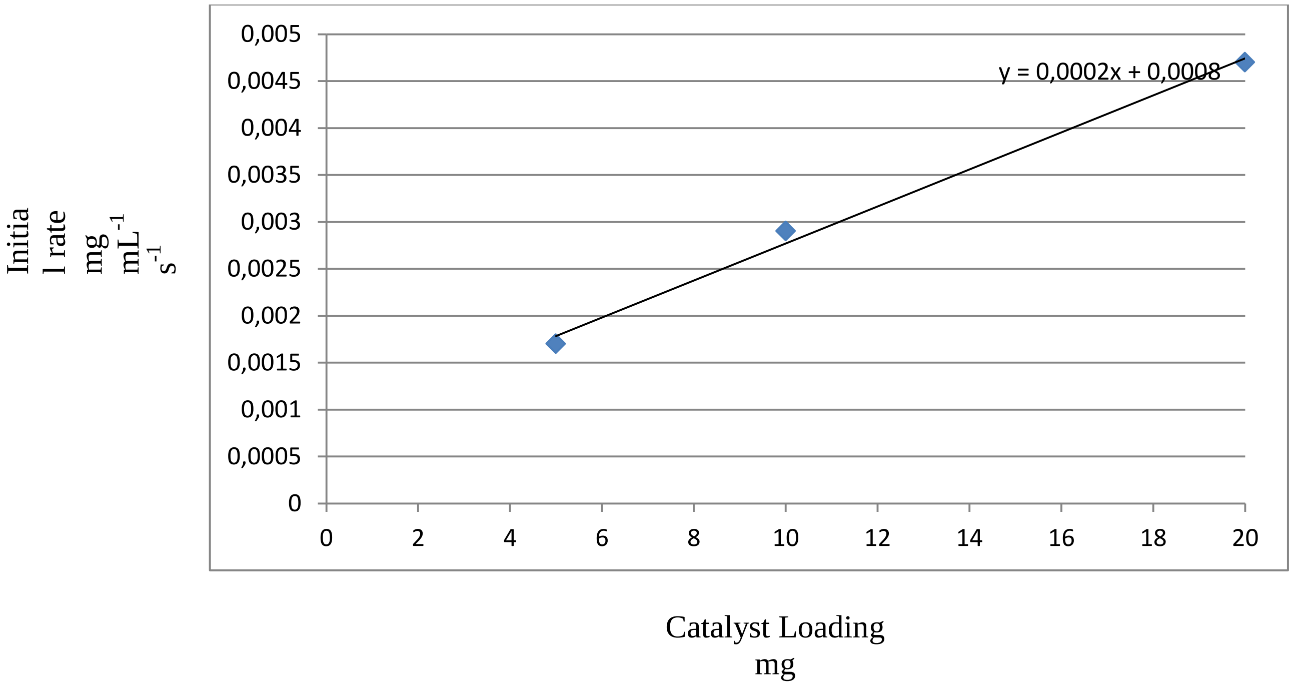

7.4.2 Order in naphthalene

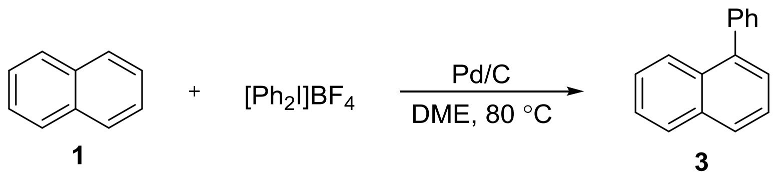

Reactions were undertaken as per general procedure A. Naphthalene concentrations of 0.10, 0.20, 0.25, 0.30 were undertaken. Initial reaction rates were determined by measuring the rate of product formation between ~4 and ~30 % yield, with a minimum of 5 data points. Plotting the initial rates (gradient) vs. concentration of naphtalene showed an absence of correlation indicative of 0 order reaction kinetics.

Plot of concentration (1) vs time

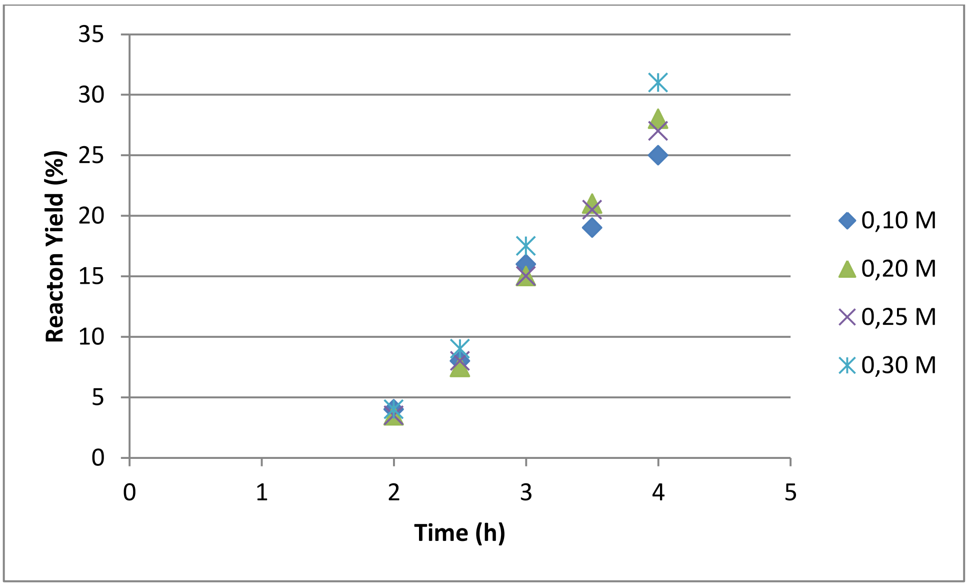

Plot of initial rate vs naphthalene (1) concentration

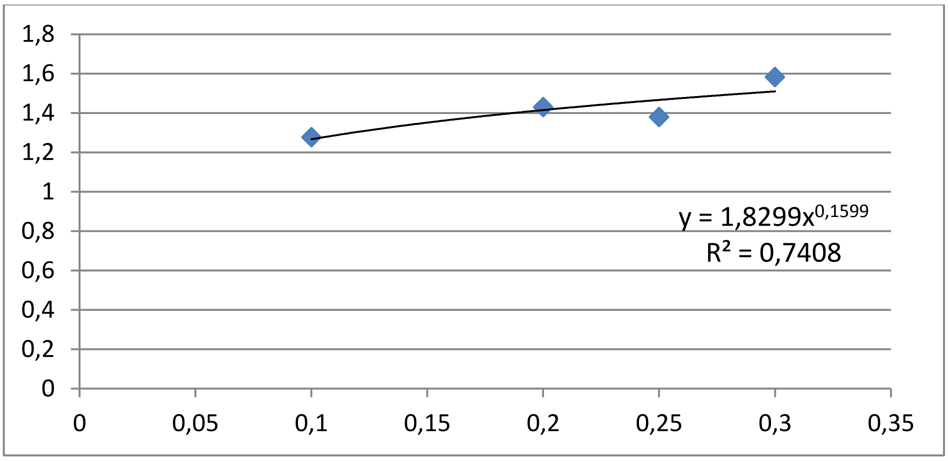

7.5 Reaction of PhI_2BF_4 in the absence of PAH

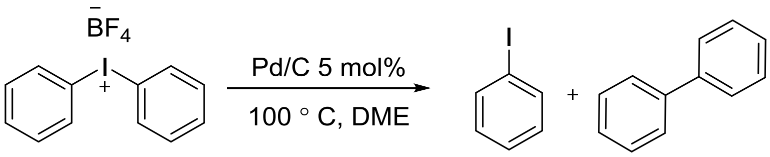

PhI_2BF_4 (28 mg, 0.076 mmol) and Pd/C (8 mg, 5 mol%) were heated at 100 ºC in DME (0.25 mL).

	**Yield %[a] 2 h**	**Yield % [a] 5 h**	**Yield % [a] 22 h**
PhI	80	80	80

[a]Yields determined by GC of crude reaction mixture, mesitylene as internal standard.

7.6 Deuteration experiments

Pd/C (5 mol%) was pre-treated with PIDA (20 mol%) at 100 °C in DME (0.25 ml) for 2 h to form the active catalytic species.

Naphthalene (**1**) (0.150 mmol) and pre-activated Pd/C (5 mol%) and MeOD-d_4 (14.5 μL) were stirred overnight at 80 °C in DME (0.25 mL). Subsequent NMR analysis indicated no deuterium incorporation.

Triphenylene (**4**) (0.150 mmol) and pre-activated Pd/C (5 mol%) and MeOD-d_4 (14.5 μL) were stirred overnight at 100 °C in DME (0.25 mL). Subsequent NMR analysis indicated no deuterium incorporation.

8.0 Failed or unselective reactions

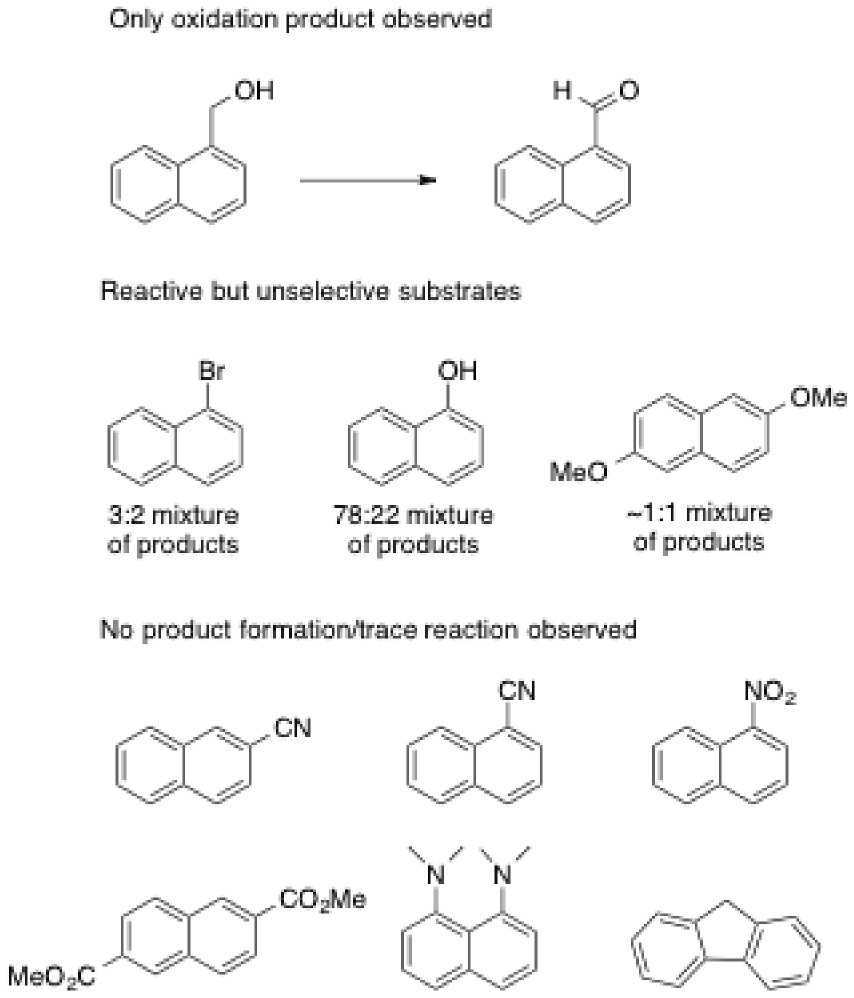

Reaction conditions as general procedure A.

9.0 NMR Spectra

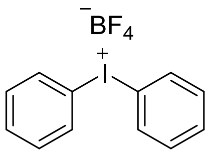

Diphenyliodonium tetrafluoroborate

^{1}H-NMR (300 MHz, DMSO-d_6)

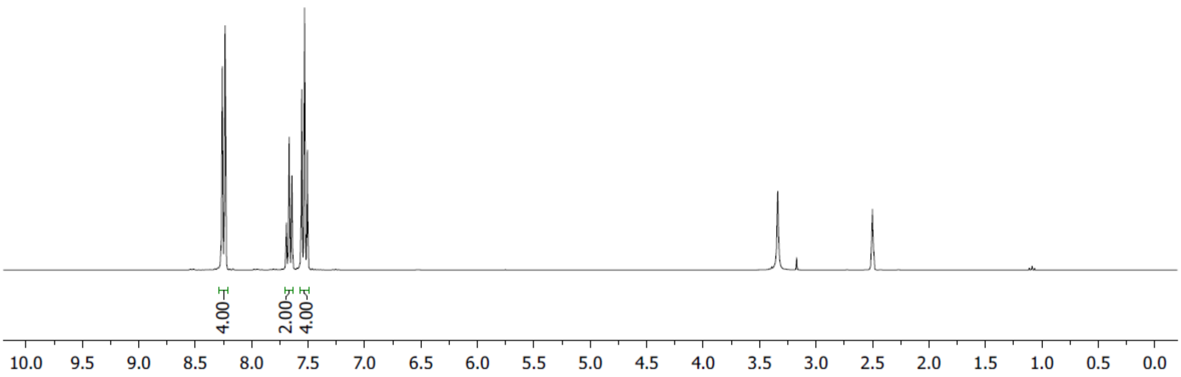

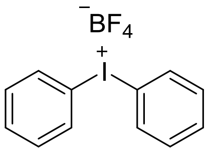

Diphenyliodonium tetrafluoroborate

^{13}C-NMR (75 MHz, DMSO-d_6)

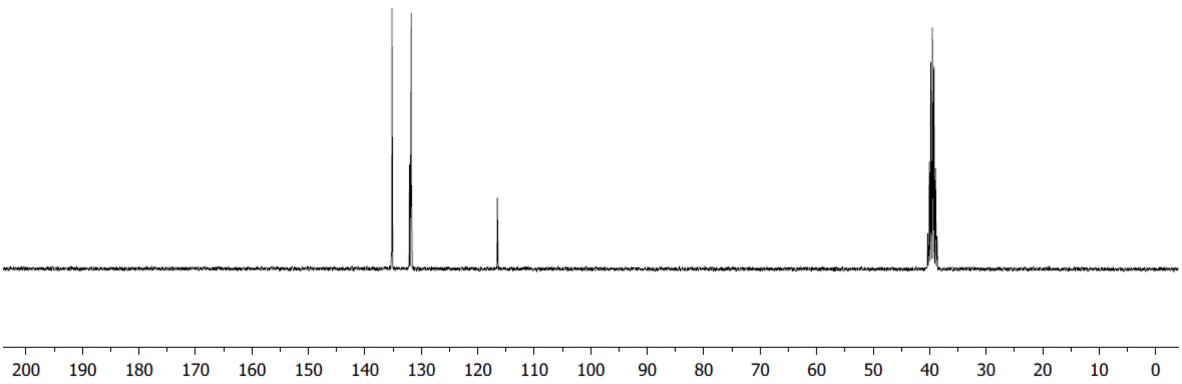

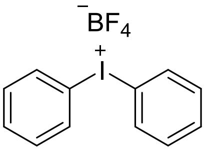

Diphenyliodonium tetrafluoroborate

^{19}F-NMR (282 MHz, DMSO-d$_6$)

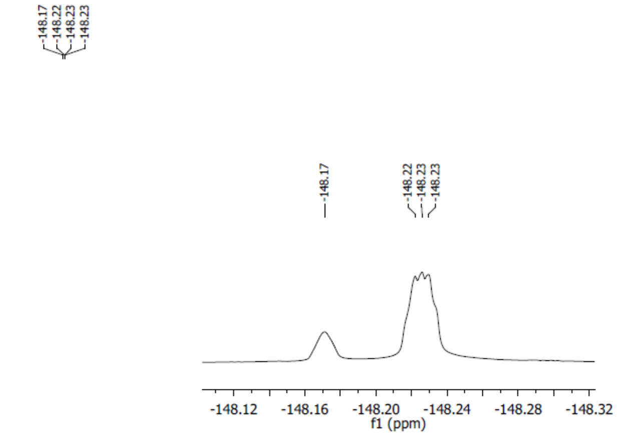

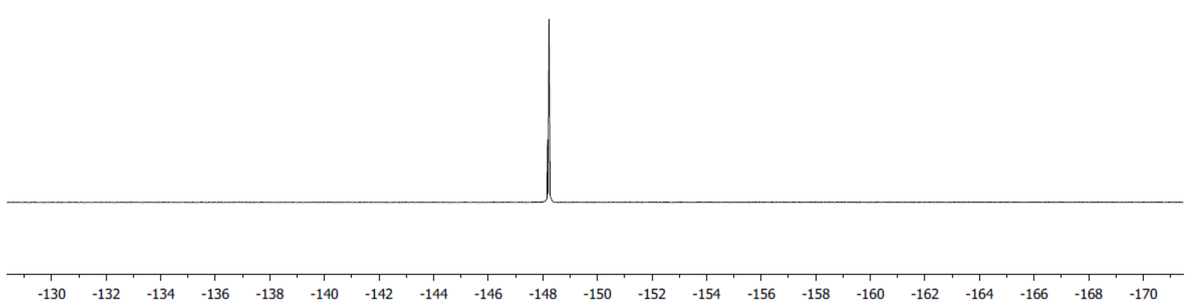

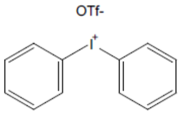

Diphenyliodonium triflate

^{1}H-NMR (300 MHz, DMSO-d$_6$)

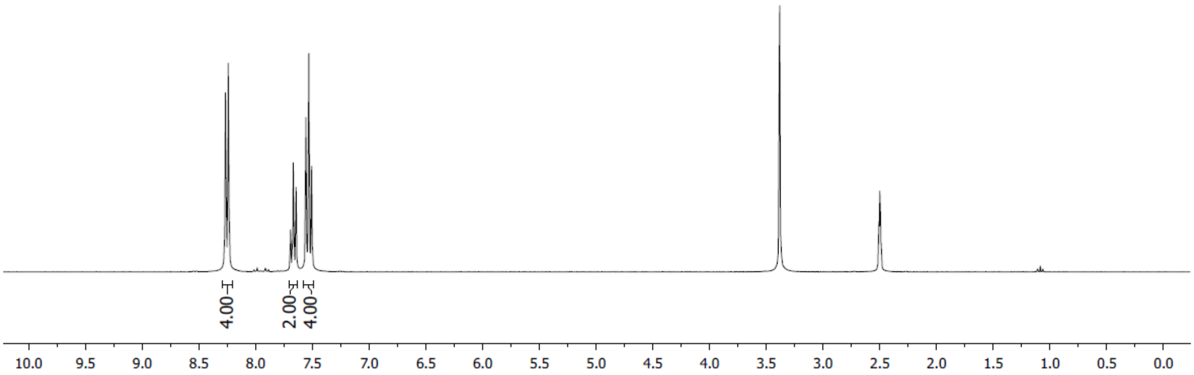

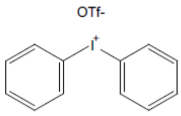

Diphenyliodonium triflate
^{13}C-NMR (75 MHz, DMSO-d_6)

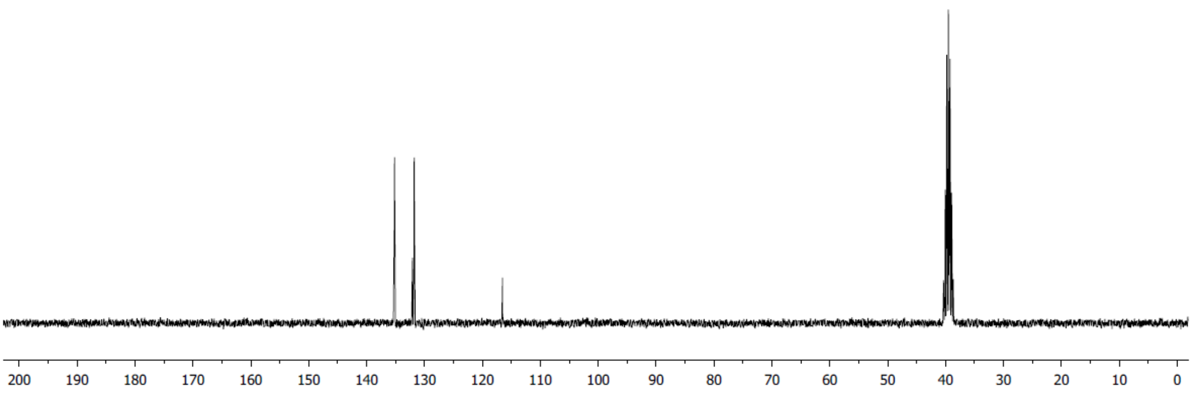

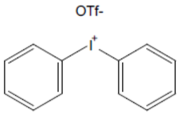

Diphenyliodonium triflate
^{19}F-NMR (282 MHz, DMSO-d_6)

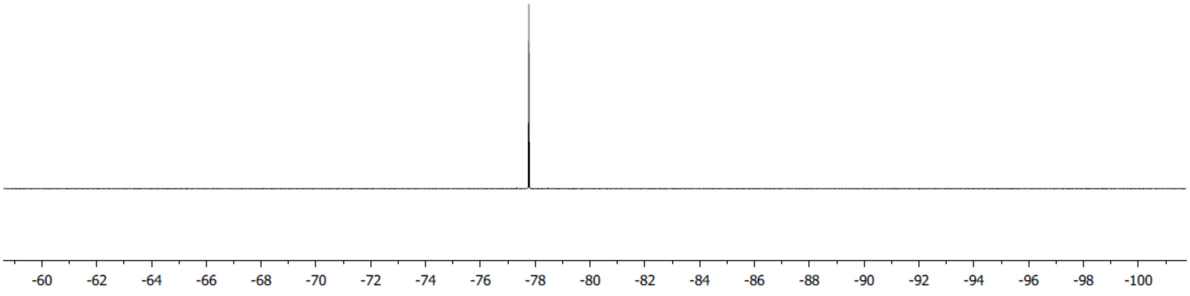

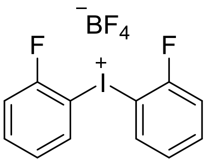

Bis(2-fluorophenyl)iodonium tetrafluoroborate

^{1}H-NMR (300 MHz, DMSO-d_6)

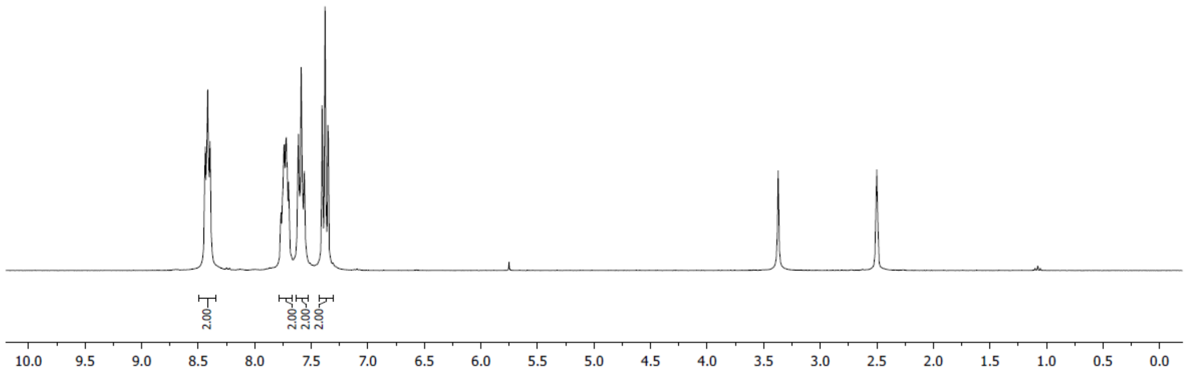

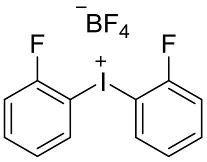

Bis(2-fluorophenyl)iodonium tetrafluoroborate

^{13}C-NMR (75 MHz, DMSO-d_6)

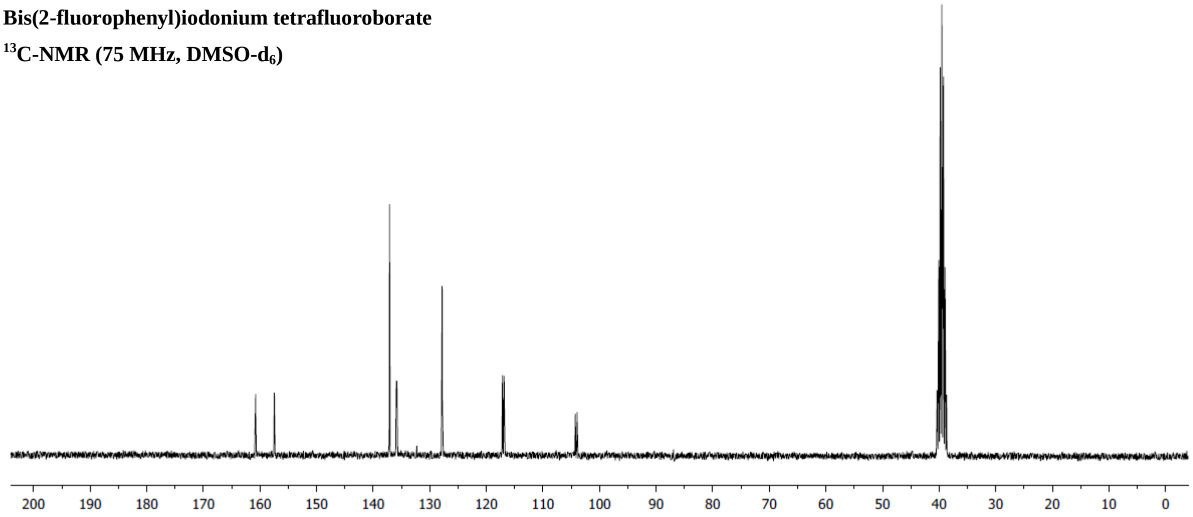

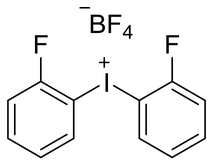

Bis(2-fluorophenyl)iodonium tetrafluoroborate

^{19}F{^{1}H}-NMR (282 MHz, CDCl$_3$)

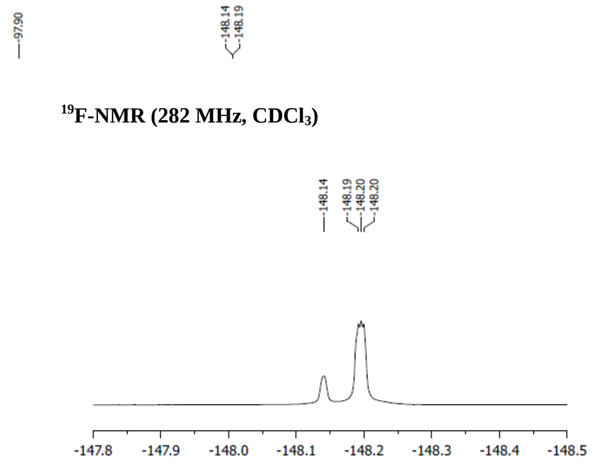

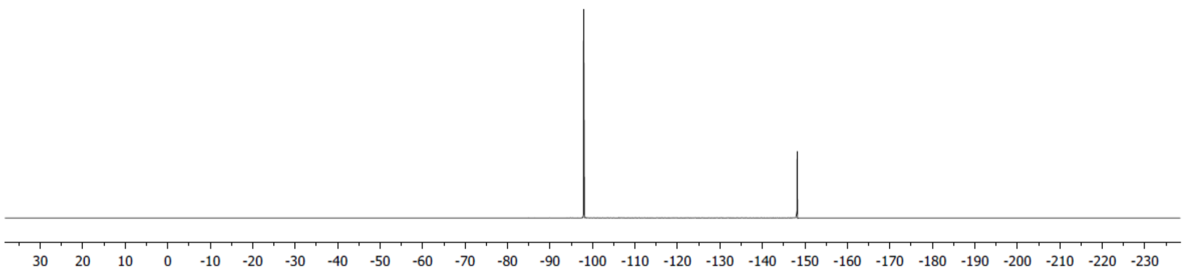

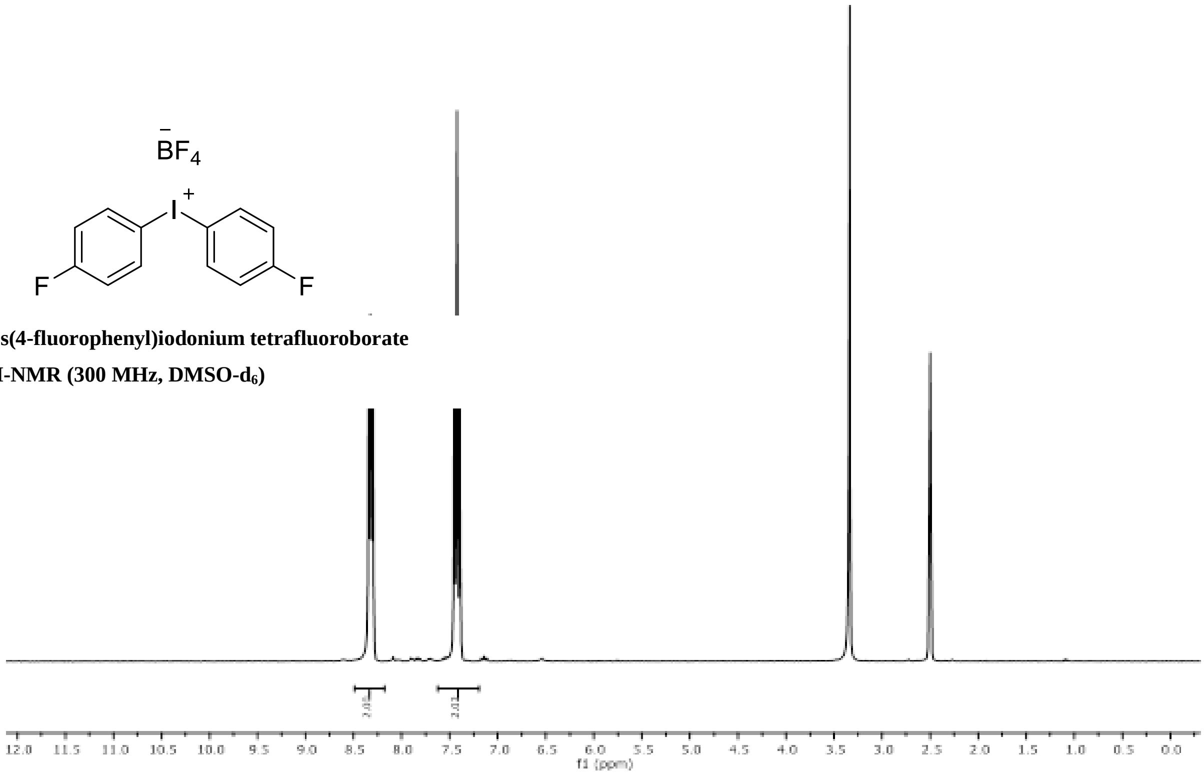

Bis(4-fluorophenyl)iodonium tetrafluoroborate

^{1}H-NMR (300 MHz, DMSO-d_6)

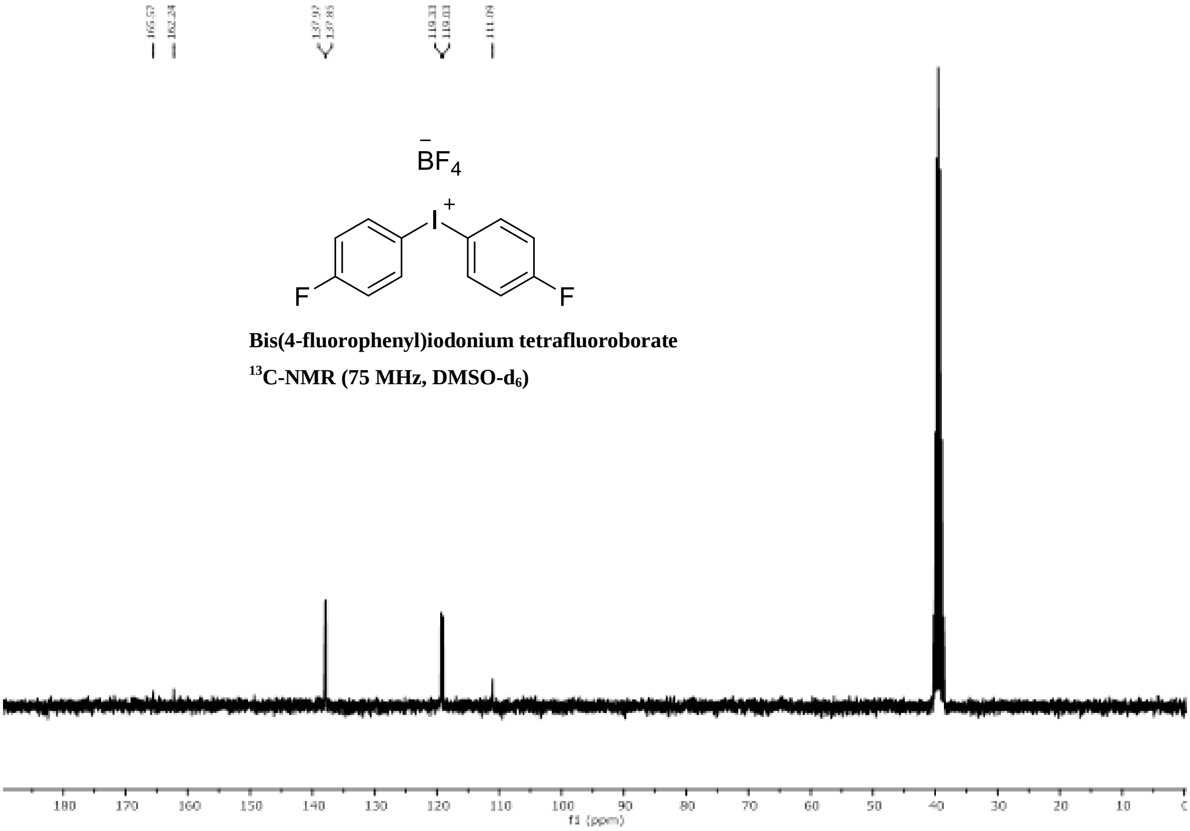

Bis(4-fluorophenyl)iodonium tetrafluoroborate

^{13}C-NMR (75 MHz, DMSO-d_6)

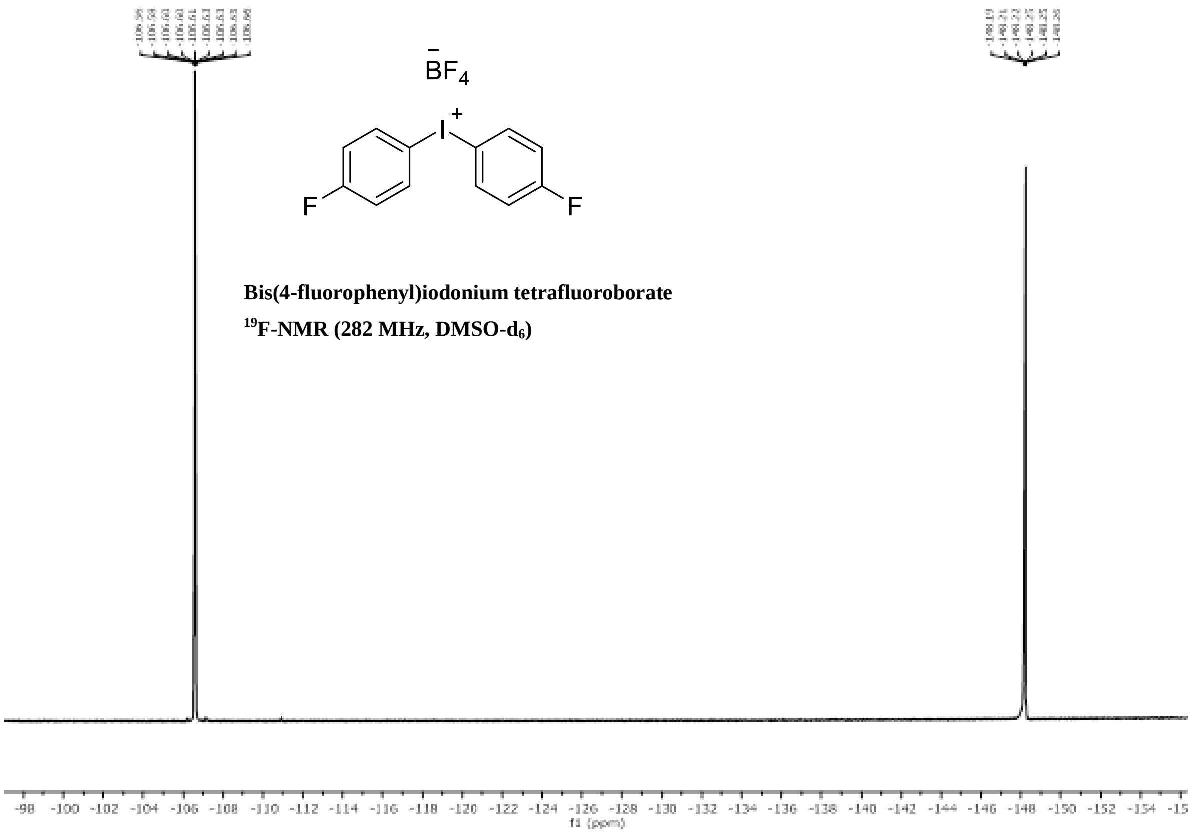

Bis(4-fluorophenyl)iodonium tetrafluoroborate

^{19}F-NMR (282 MHz, DMSO-d_6)

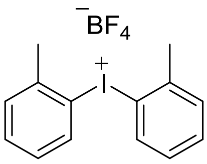

Bis(2-methylphenyl)iodonium tetrafluoroborate

^{1}H-NMR (300 MHz, DMSO-d_6)

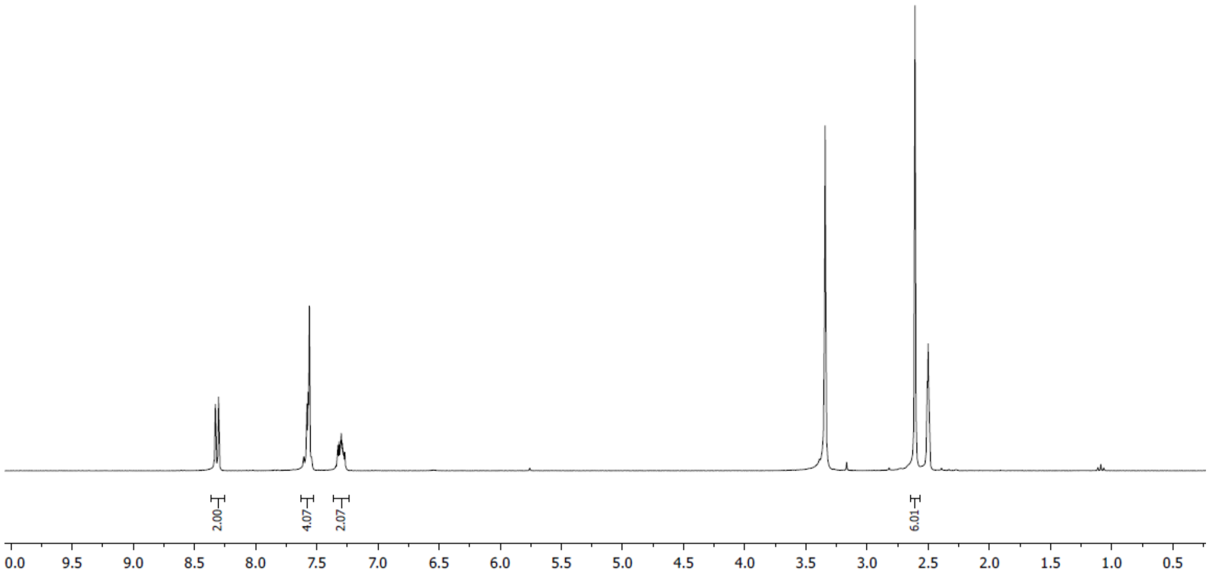

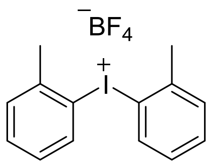

Bis(2-methylphenyl)iodonium tetrafluoroborate

^{13}C-NMR (75 MHz, DMSO-d₆)

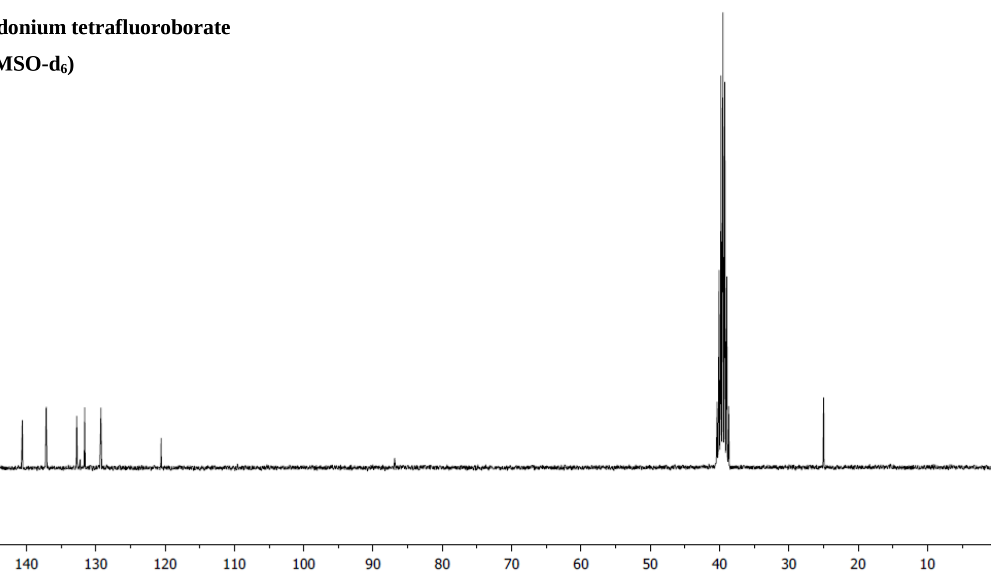

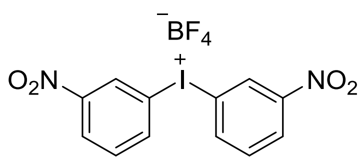

Bis(3-nitrophenyl)iodonium tetrafluoroborate

^{1}H-NMR (400 MHz, DMSO-d₆)

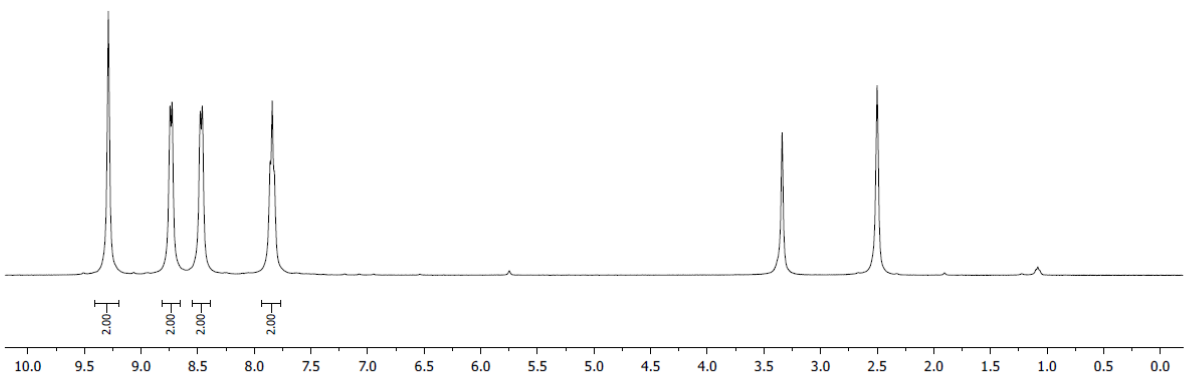

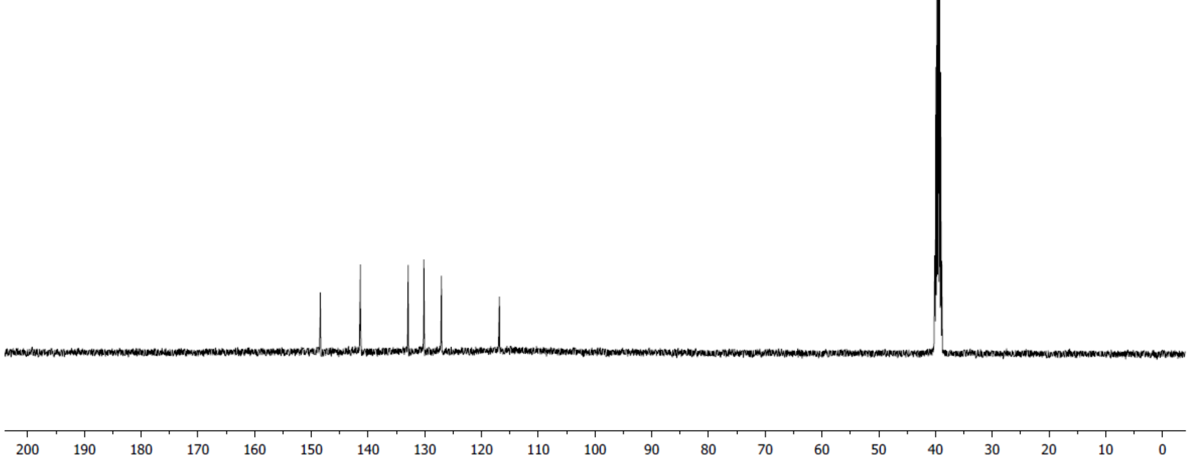

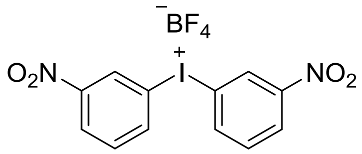

Bis(3-nitrophenyl)iodonium tetrafluoroborate

^{13}C-NMR (101 MHz, DMSO-d$_6$)

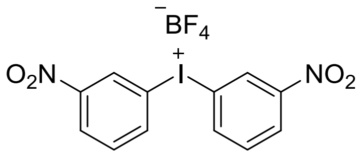

Bis(2-fluorophenyl)iodonium tetrafluoroborate

^{19}F-NMR (282 MHz, DMSO-d$_6$)

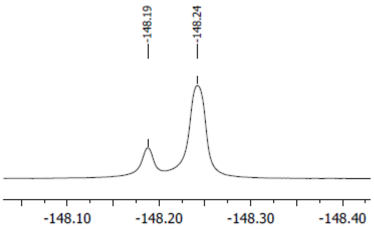

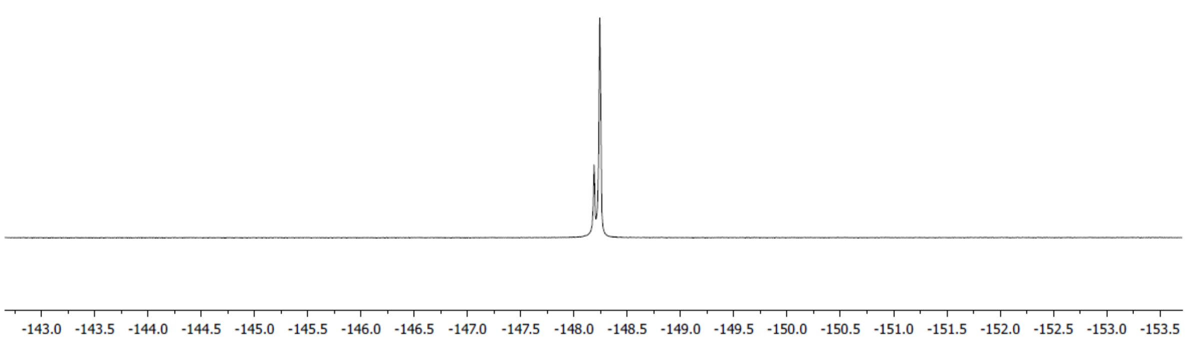

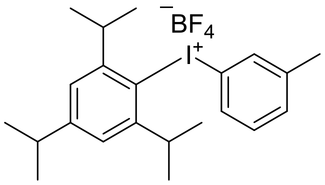

(3-Methylphenyl)(2,4,6-triisopropylphenyl)iodonium tetrafluoroborate

^{1}H-NMR (300 MHz, $CDCl_3$)

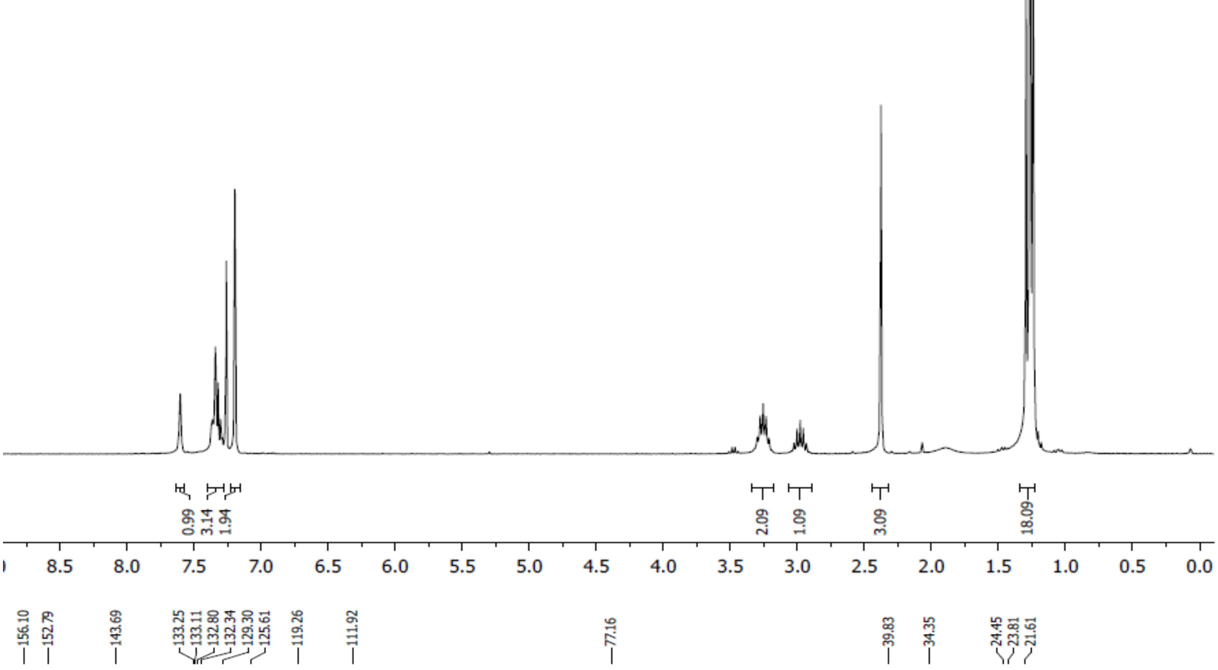

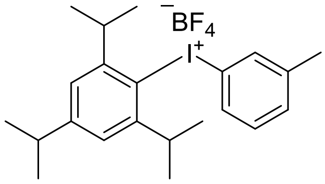

(3-Methylphenyl)(2,4,6-triisopropylphenyl)iodonium tetrafluoroborate

^{13}C-NMR (75 MHz, $CDCl_3$)

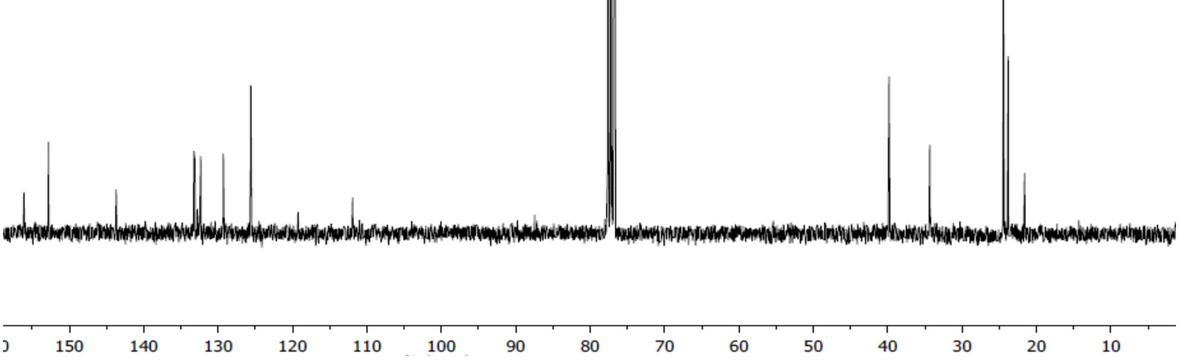

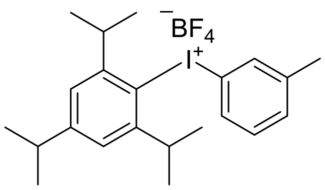

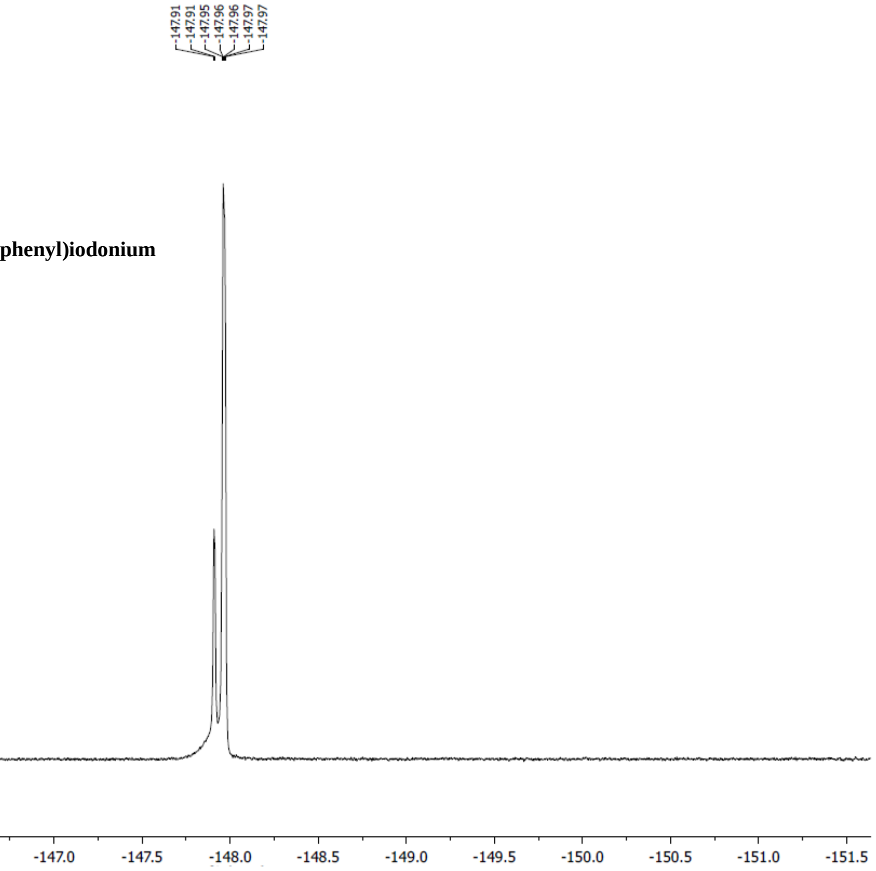

(3-Methylphenyl)(2,4,6-triisopropylphenyl)iodonium tetrafluoroborate

^{19}F-NMR (282 MHz, $CDCl_3$)

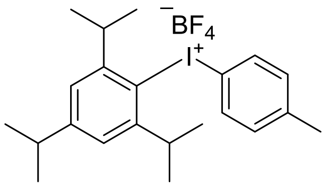

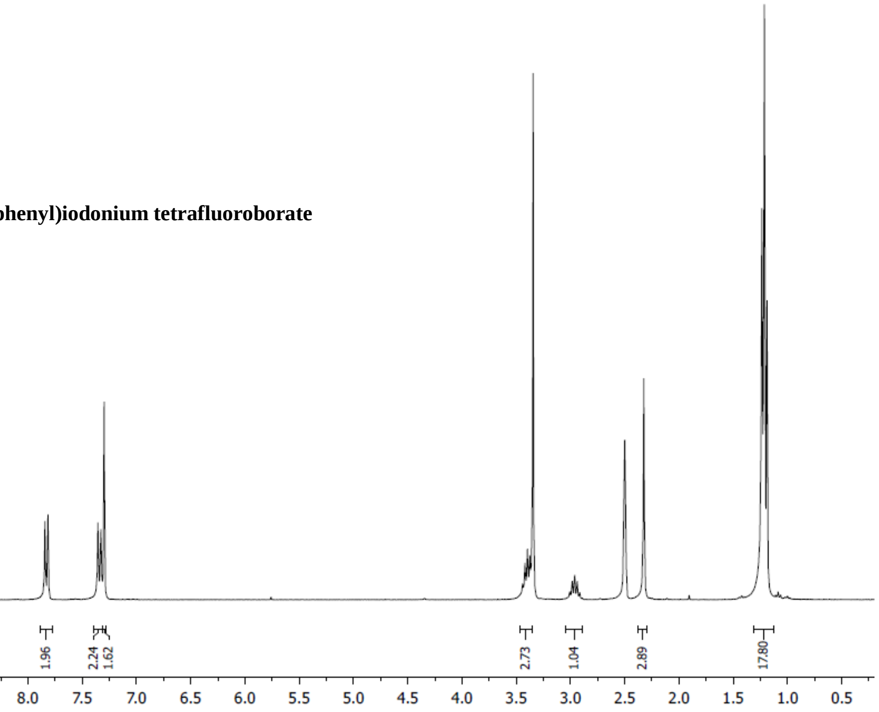

(4-Methylphenyl)(2,4,6-triisopropylphenyl)iodonium tetrafluoroborate

^{1}H-NMR (300 MHz, DMSO-d_6)

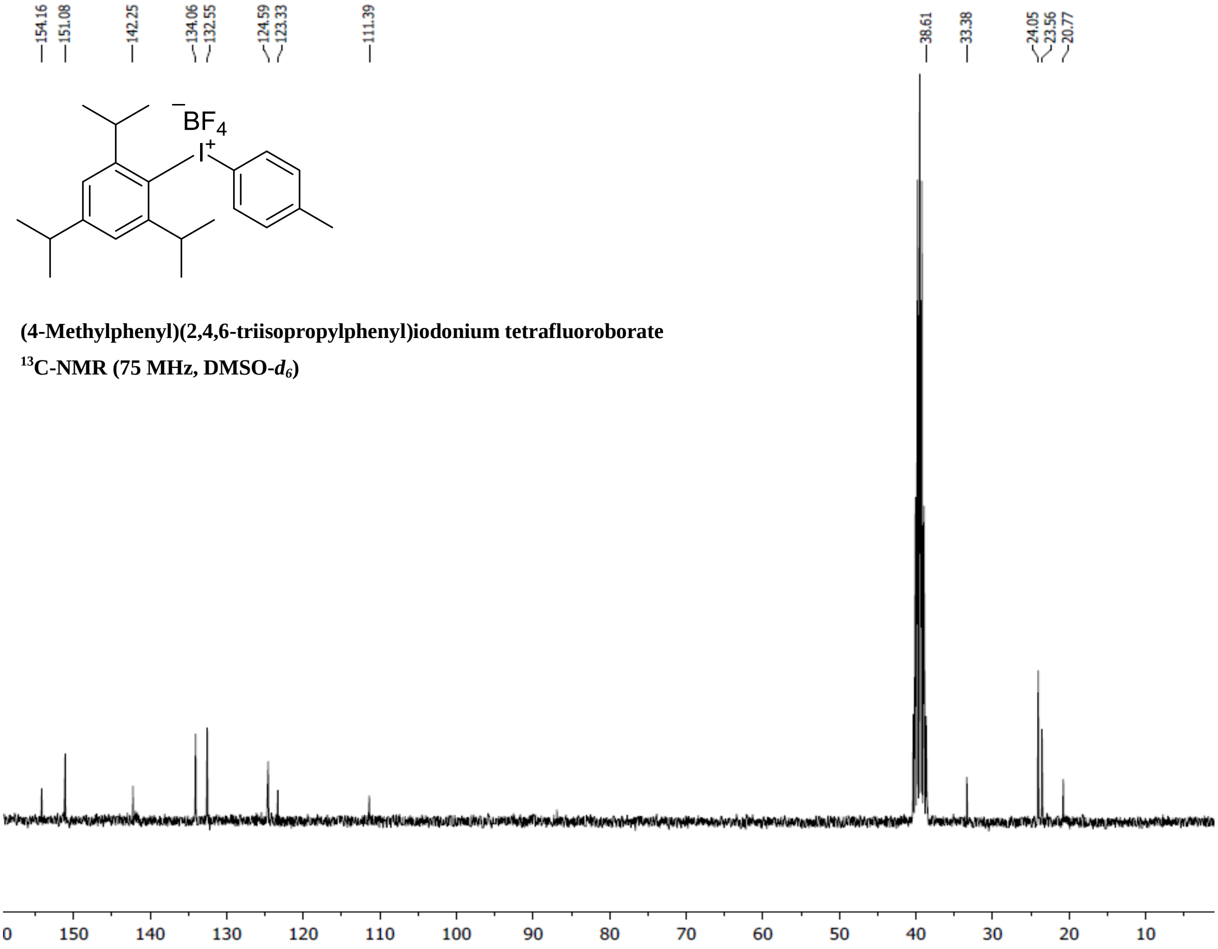

(4-Methylphenyl)(2,4,6-triisopropylphenyl)iodonium tetrafluoroborate
^{13}C-NMR (75 MHz, DMSO-d_6)

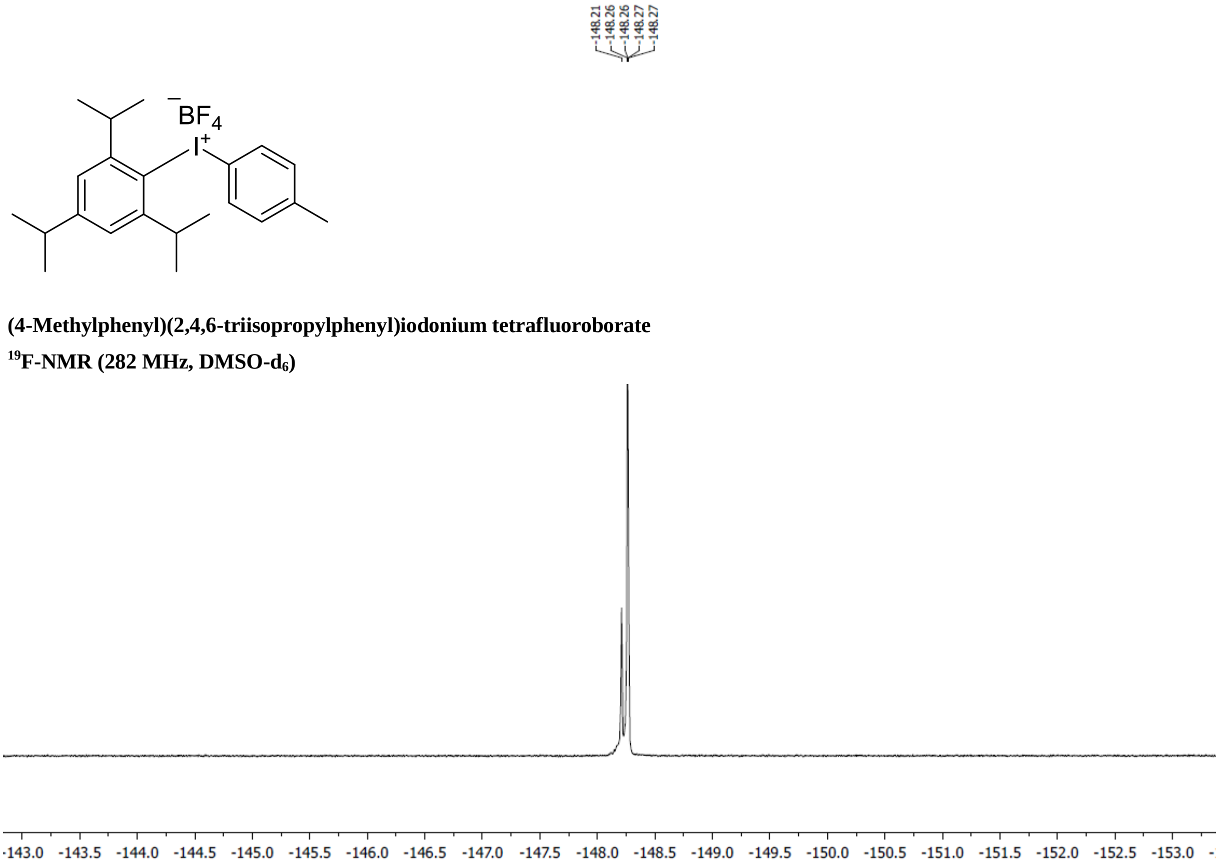

(4-Methylphenyl)(2,4,6-triisopropylphenyl)iodonium tetrafluoroborate
^{19}F-NMR (282 MHz, DMSO-d_6)

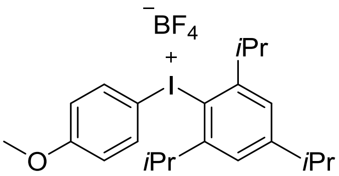

(4-Methoxyphenyl)(2,4,6-triisopropylphenyl)iodonium tetrafluoroborate

^{1}H-NMR (300 MHz, CDCl$_3$)

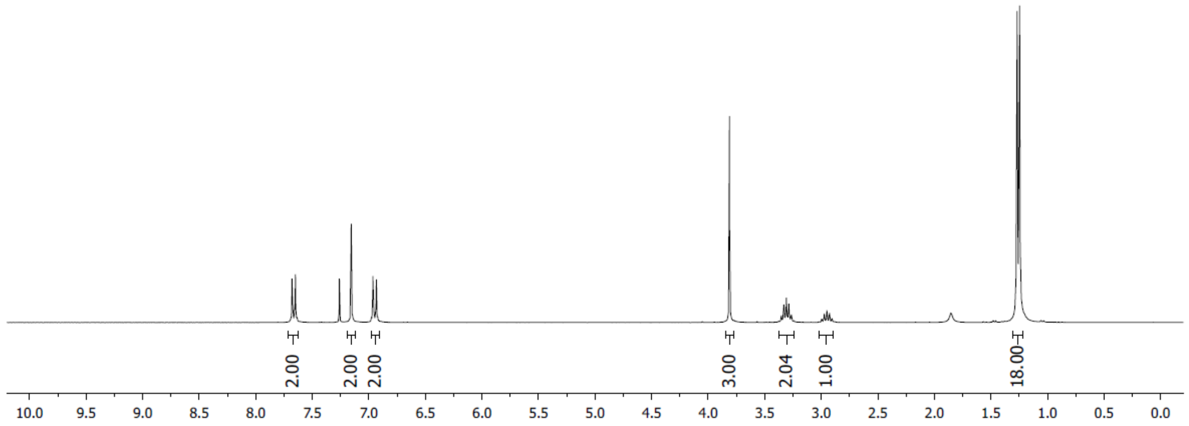

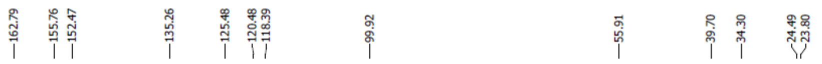

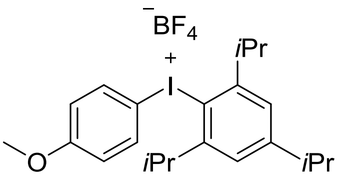

(4-Methoxyphenyl)(2,4,6-triisopropylphenyl)iodonium tetrafluoroborate

^{13}C-NMR (75 MHz, CDCl$_3$)

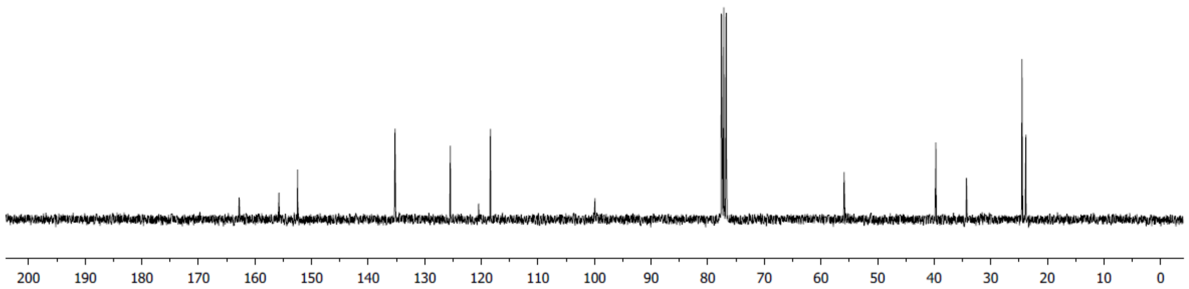

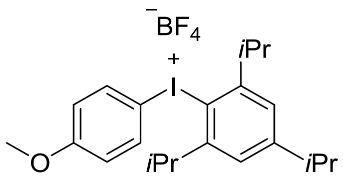

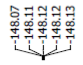

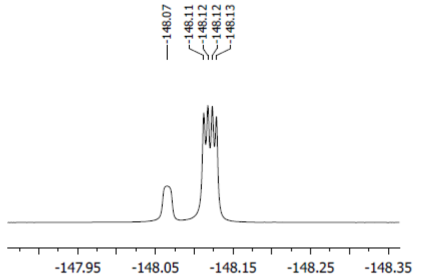

(4-Methoxyphenyl)(2,4,6-triisopropylphenyl)iodonium tetrafluoroborate

^{19}F-NMR (282 MHz, CDCl$_3$)

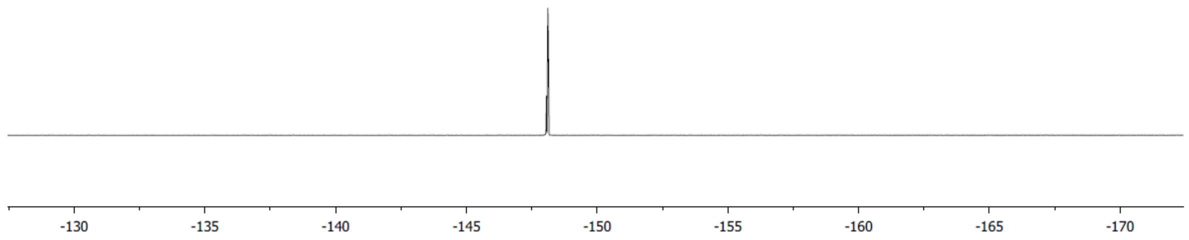

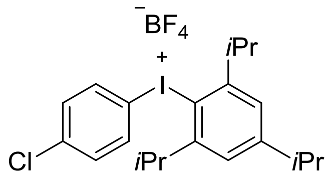

(4-Chlorophenyl)(2,4,6-triisopropylphenyl)iodonium tetrafluoroborate

^{1}H-NMR (300 MHz, CDCl$_3$)

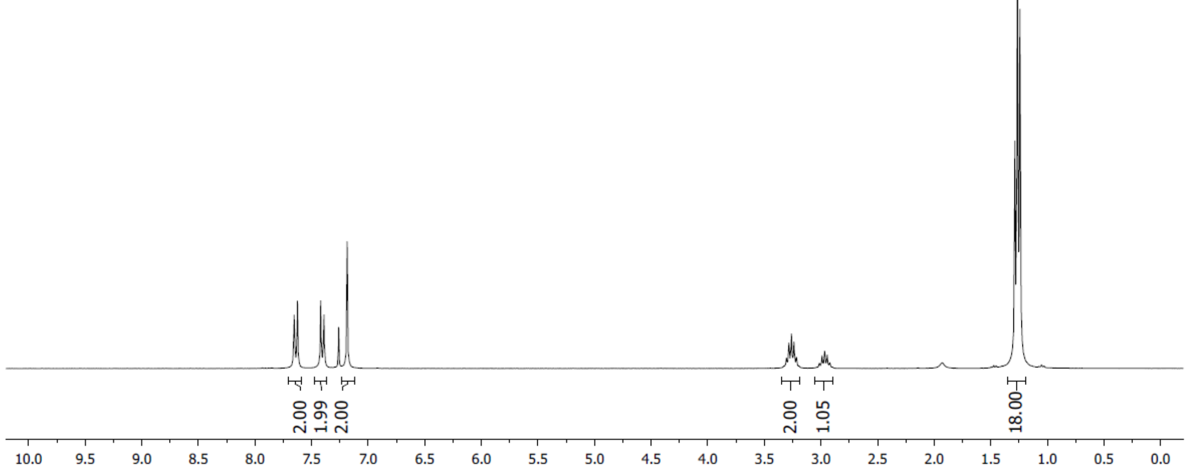

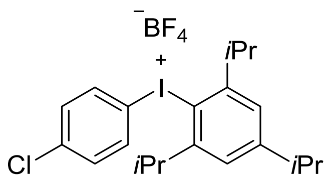

(4-Chlorophenyl)(2,4,6-triisopropylphenyl)iodonium tetrafluoroborate
^{13}C-NMR (75 MHz, $CDCl_3$)

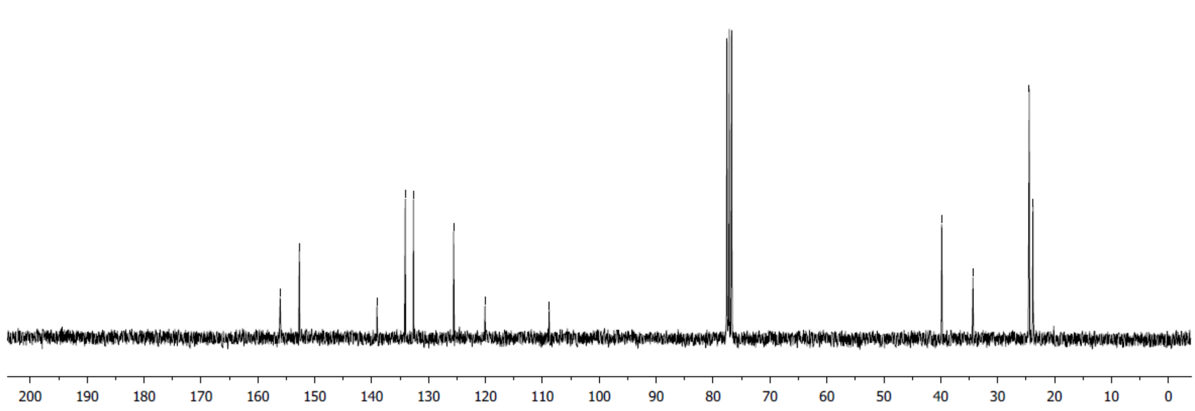

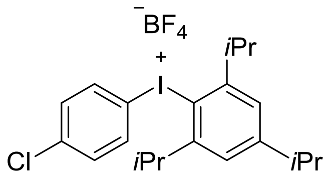

(4-Chlorophenyl)(2,4,6-triisopropylphenyl)iodonium tetrafluoroborate
^{19}F-NMR (282 MHz, $CDCl_3$)

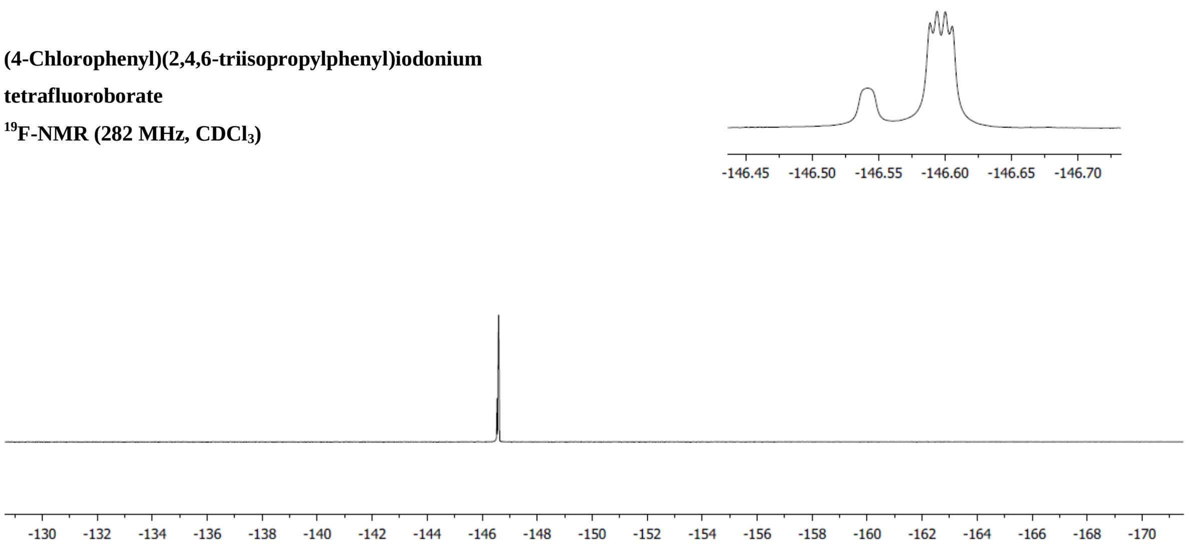

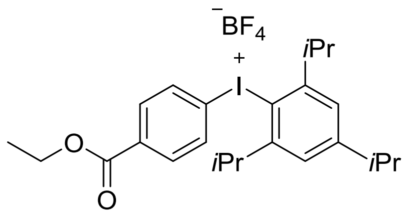

(4-Ethoxycarbonylphenyl)(2,4,6-triisopropylphenyl)iodonium tetrafluoroborate

^{1}H-NMR (300 MHz, CDCl$_3$)

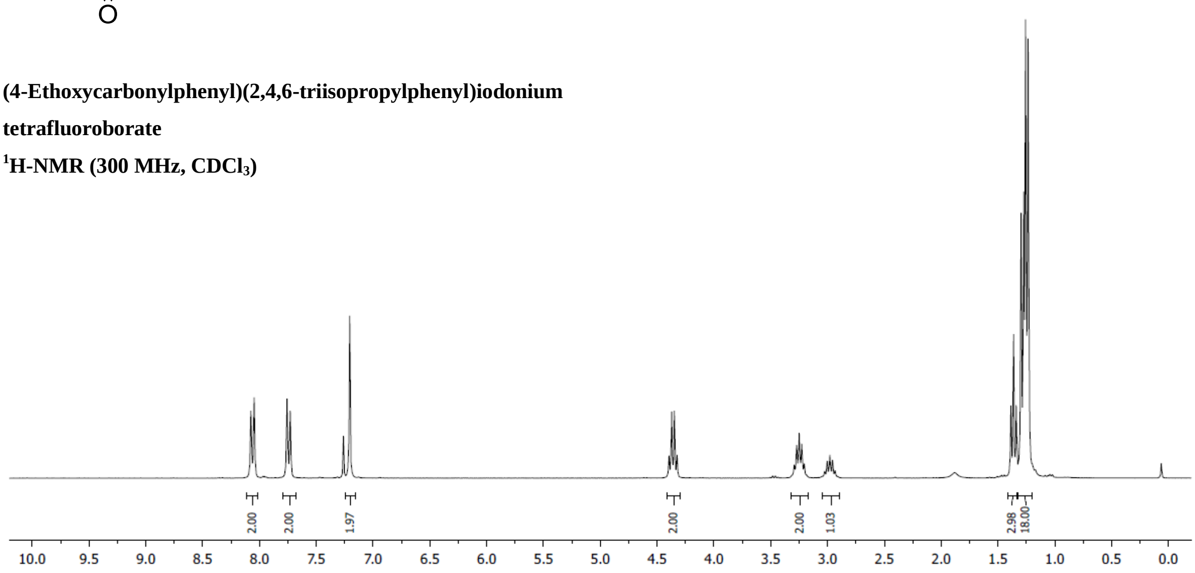

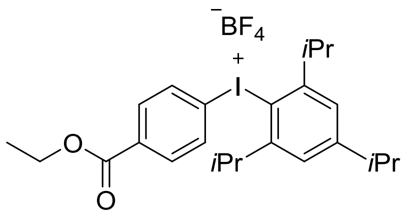

(4-Ethoxycarbonylphenyl)(2,4,6-triisopropylphenyl)iodonium tetrafluoroborate

^{13}C-NMR (75 MHz, CDCl$_3$)

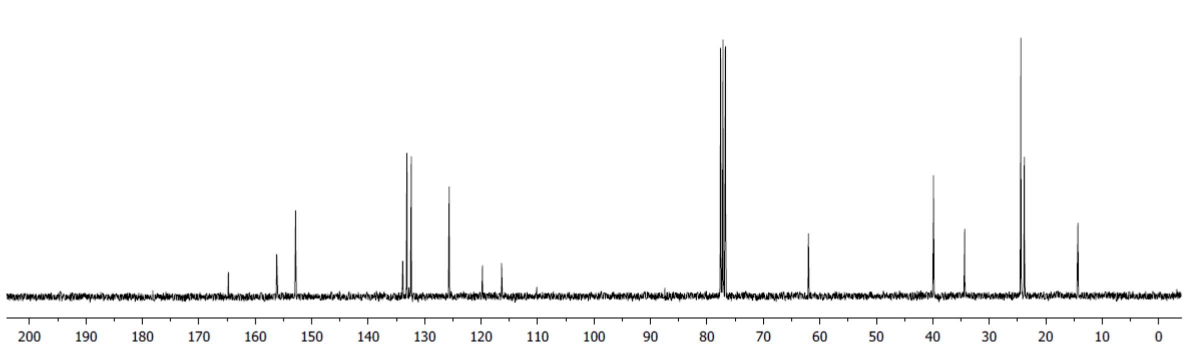

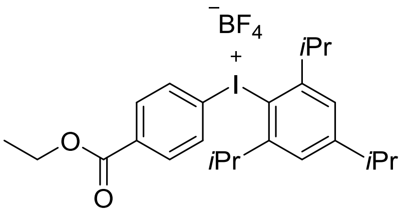

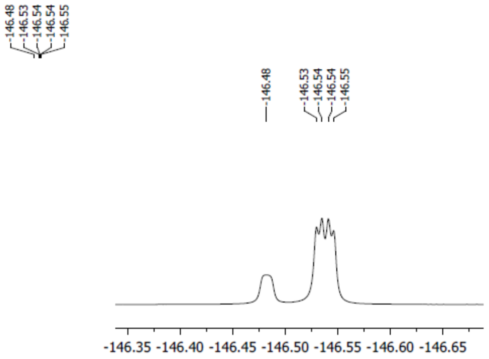

(4-Ethoxycarbonylphenyl)(2,4,6-triisopropylphenyl)iodonium tetrafluoroborate

^{19}F-NMR (282 MHz, $CDCl_3$)

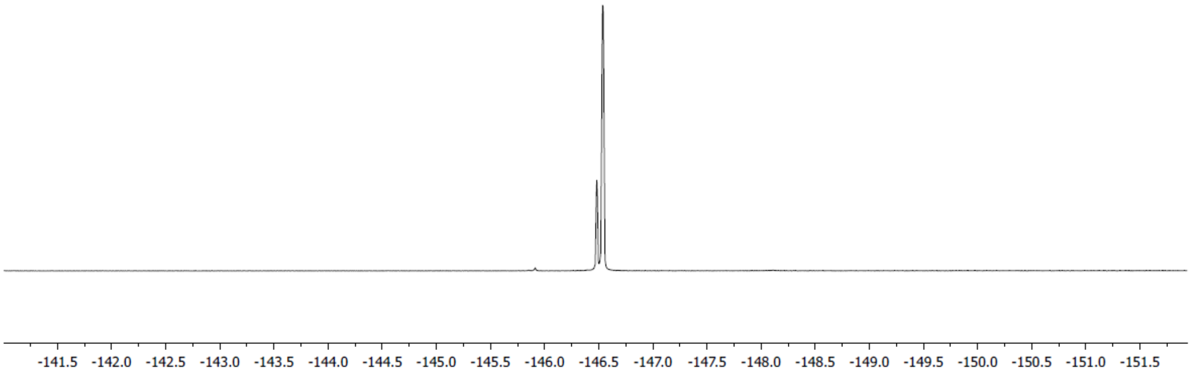

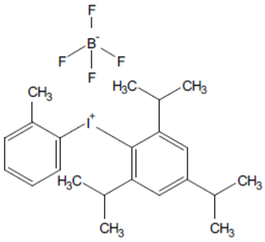

2-(Methylphenyl)(2,4,6-triisopropylphenyl)iodonium tetrafluoroborate

^{1}H-NMR (300 MHz, $CDCl_3$)

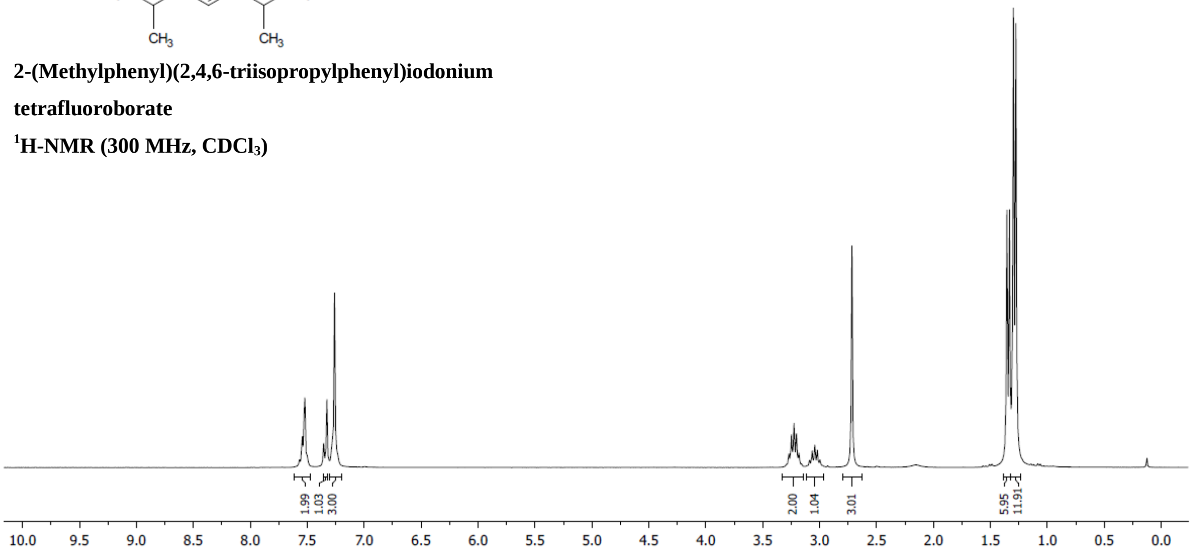

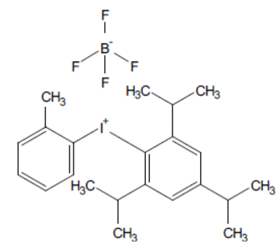

(2-Methylphenyl)(2,4,6-triisopropylphenyl)iodonium tetrafluoroborate

^{13}C-NMR (75 MHz, $CDCl_3$)

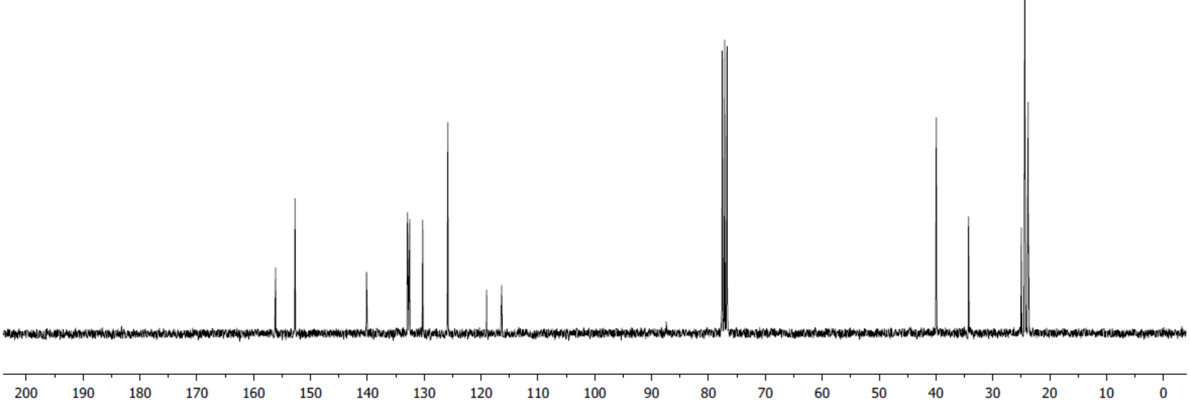

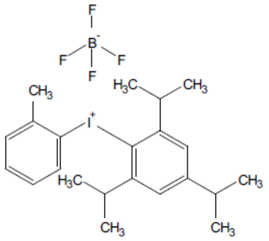

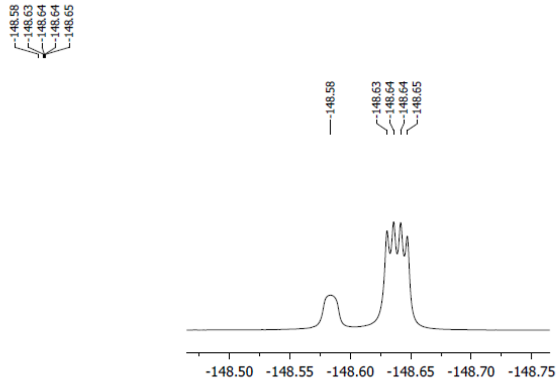

(2-Methylphenyl)(2,4,6-triisopropylphenyl)iodonium tetrafluoroborate

^{19}F-NMR (282 MHz, $CDCl_3$)

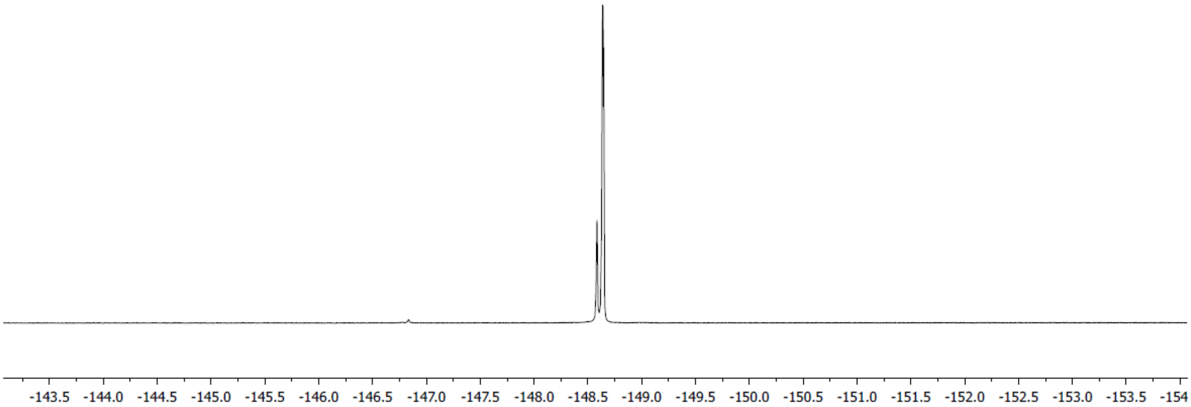

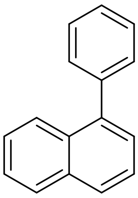

1-Phenylnaphthalene (3a)

^{1}H-NMR (400 MHz, $CDCl_3$)

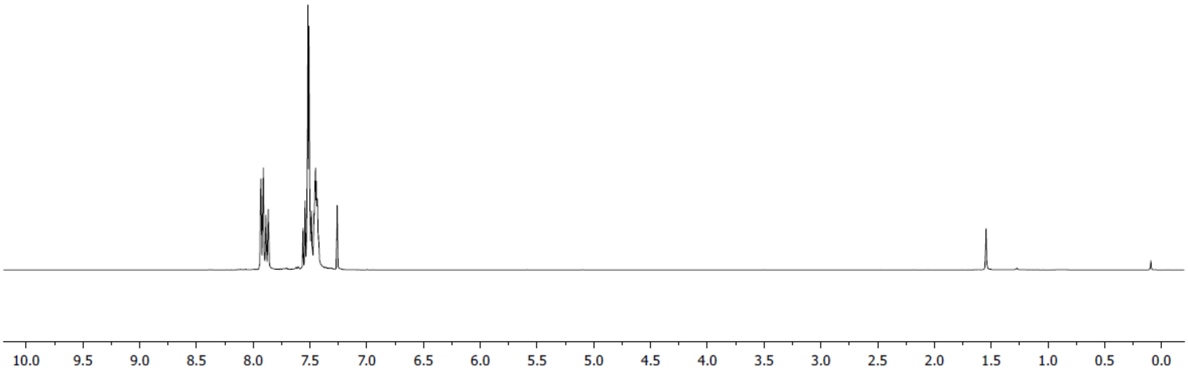

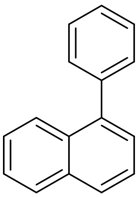

1-Phenylnaphthalene (3a)

^{13}C-NMR (101 MHz, $CDCl_3$)

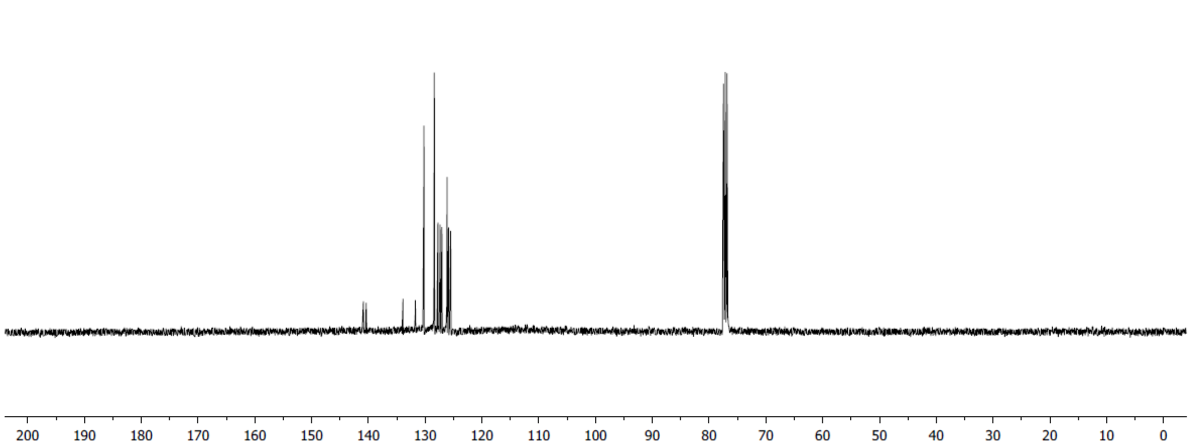

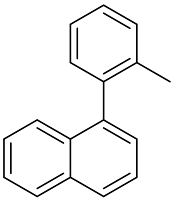

1-(2-Methylphenyl)naphthalene (3b)

^{1}H-NMR (300 MHz, $CDCl_3$)

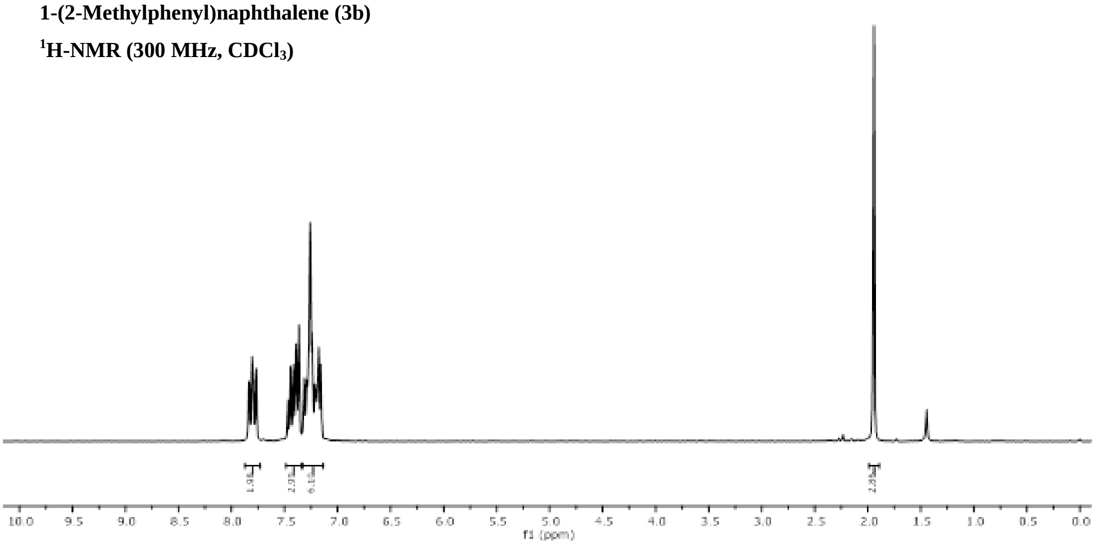

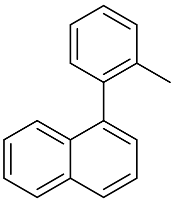

1-(2-Methylphenyl)naphthalene (3b)

^{13}C-NMR (101 MHz, $CDCl_3$)

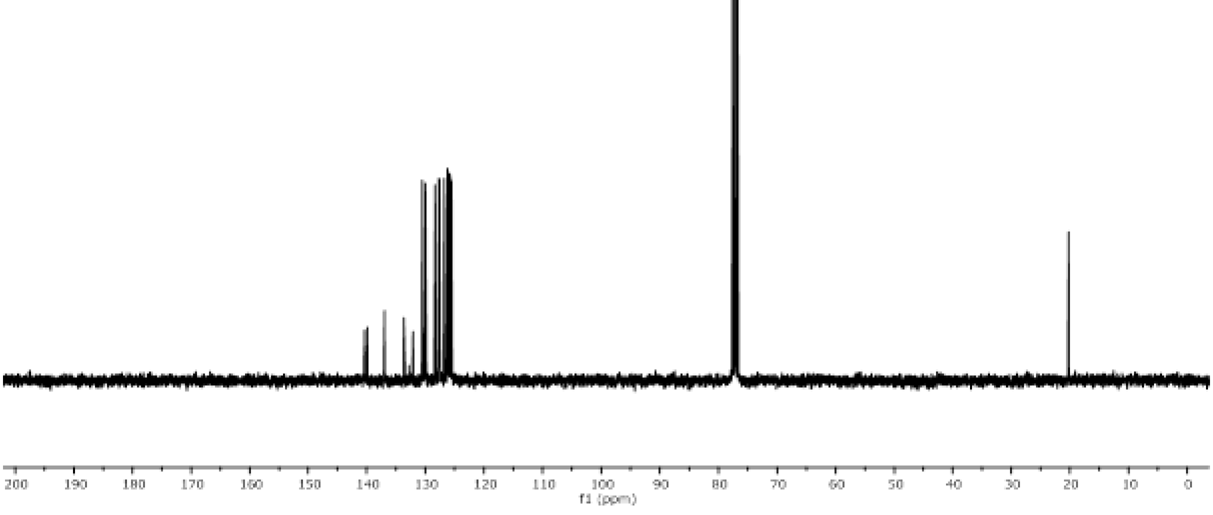

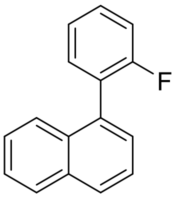

1-(2-Fluorophenyl)naphthalene (3c)

^{1}H-NMR (400 MHz, $CDCl_3$)

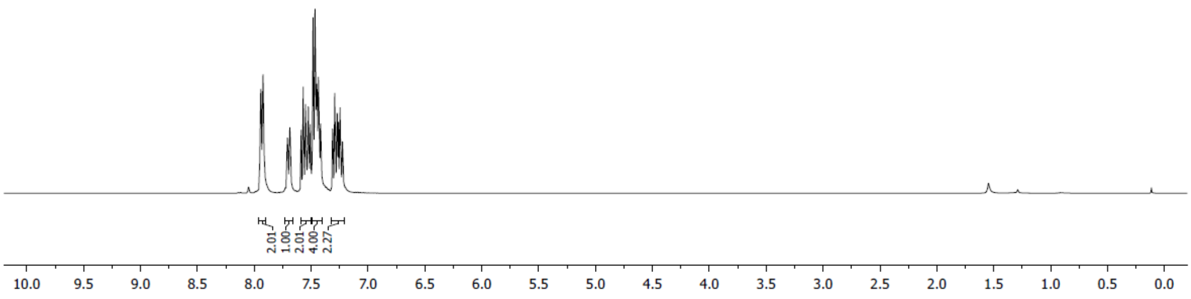

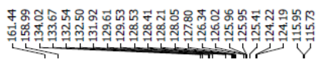

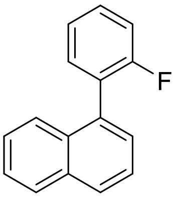

1-(2-Fluorophenyl)naphthalene (3c)

^{13}C-NMR (101 MHz, $CDCl_3$)

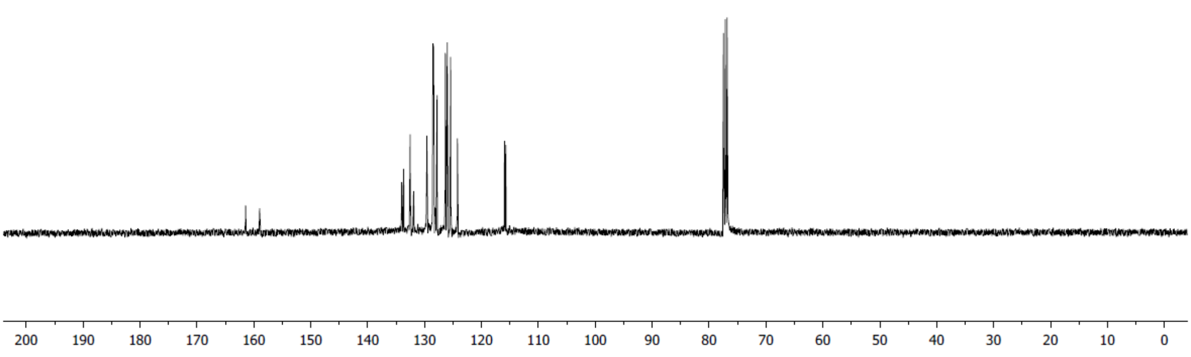

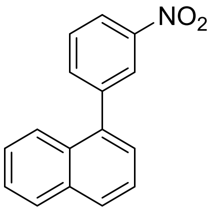

1-(3-Nitrophenyl)naphthalene (3d)

^{1}H-NMR (300 MHz, $CDCl_3$)

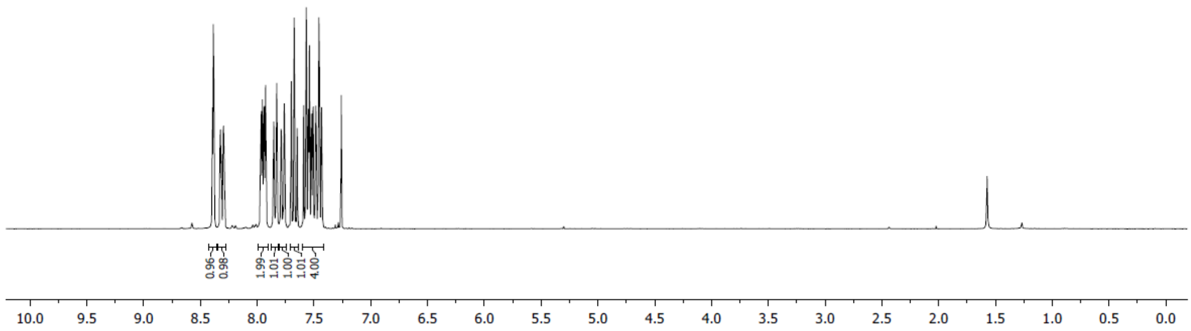

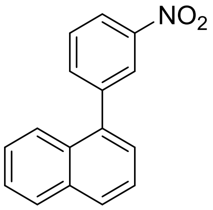

1-(3-Nitrophenyl)naphthalene (3d)

^{13}C-NMR (75 MHz, $CDCl_3$)

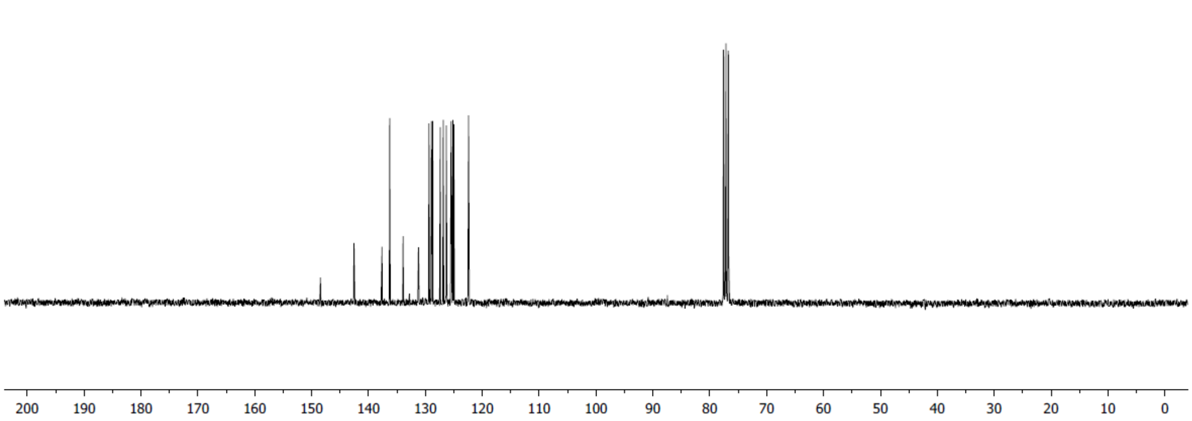

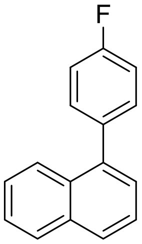

1-(4-Fluorophenyl)naphthalene (2e)

^{1}H-NMR (300 MHz, $CDCl_3$)

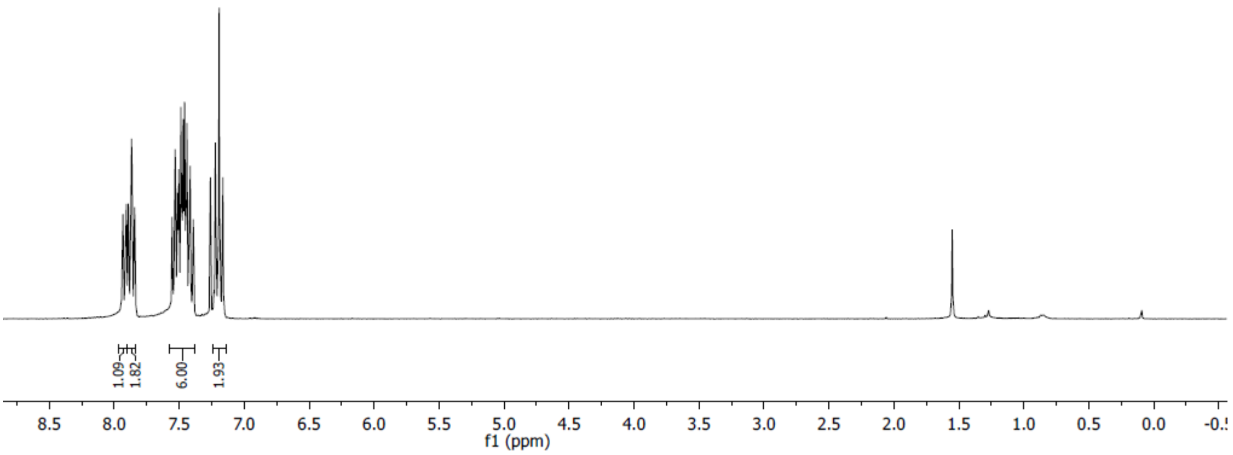

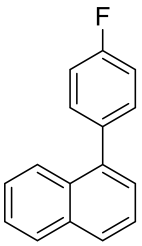

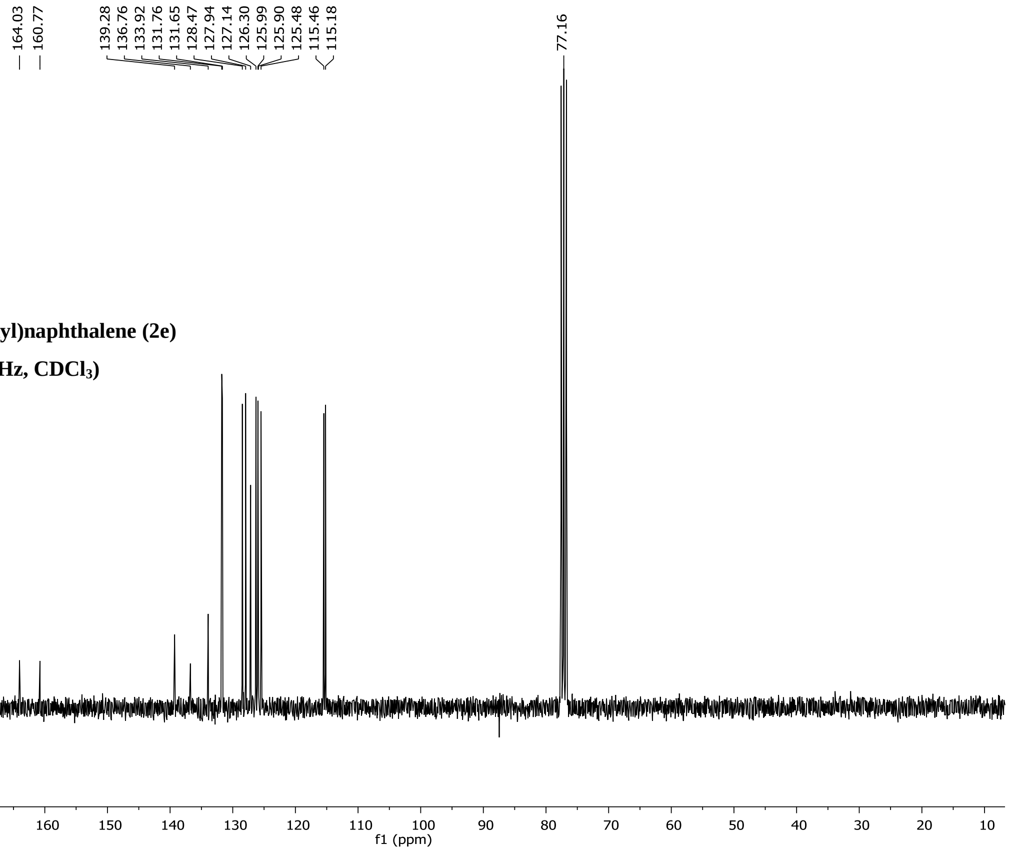

1-(4-Fluorophenyl)naphthalene (2e)

^{13}C-NMR (75 MHz, $CDCl_3$)

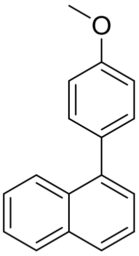

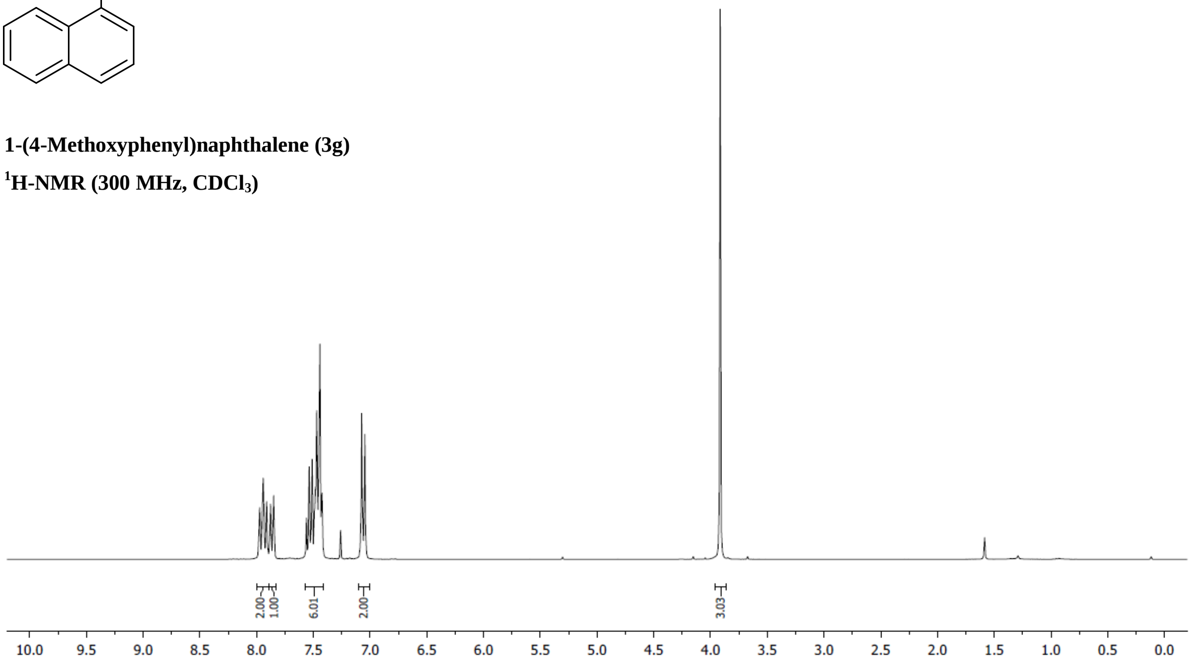

1-(4-Methoxyphenyl)naphthalene (3g)

^{1}H-NMR (300 MHz, $CDCl_3$)

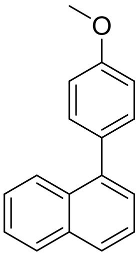

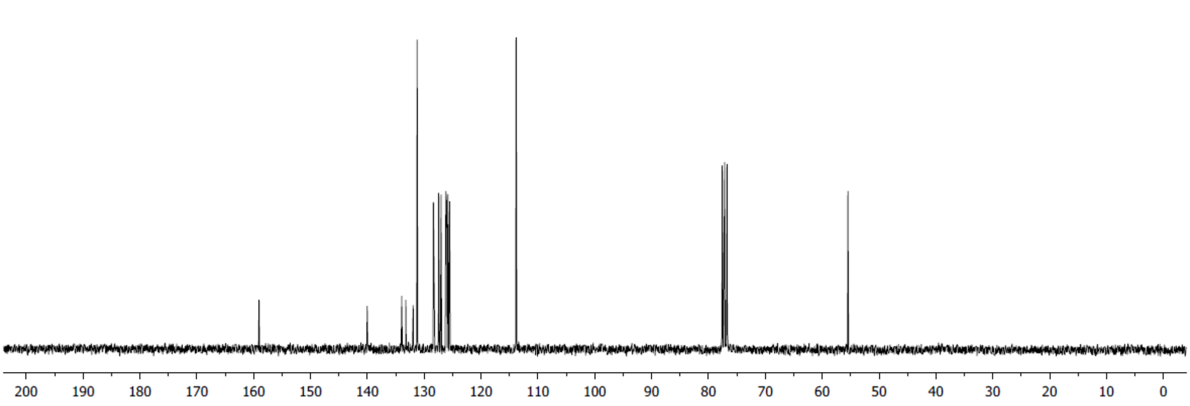

1-(4-Methoxyphenyl)naphthalene (3g)

^{13}C-NMR (75 MHz, $CDCl_3$)

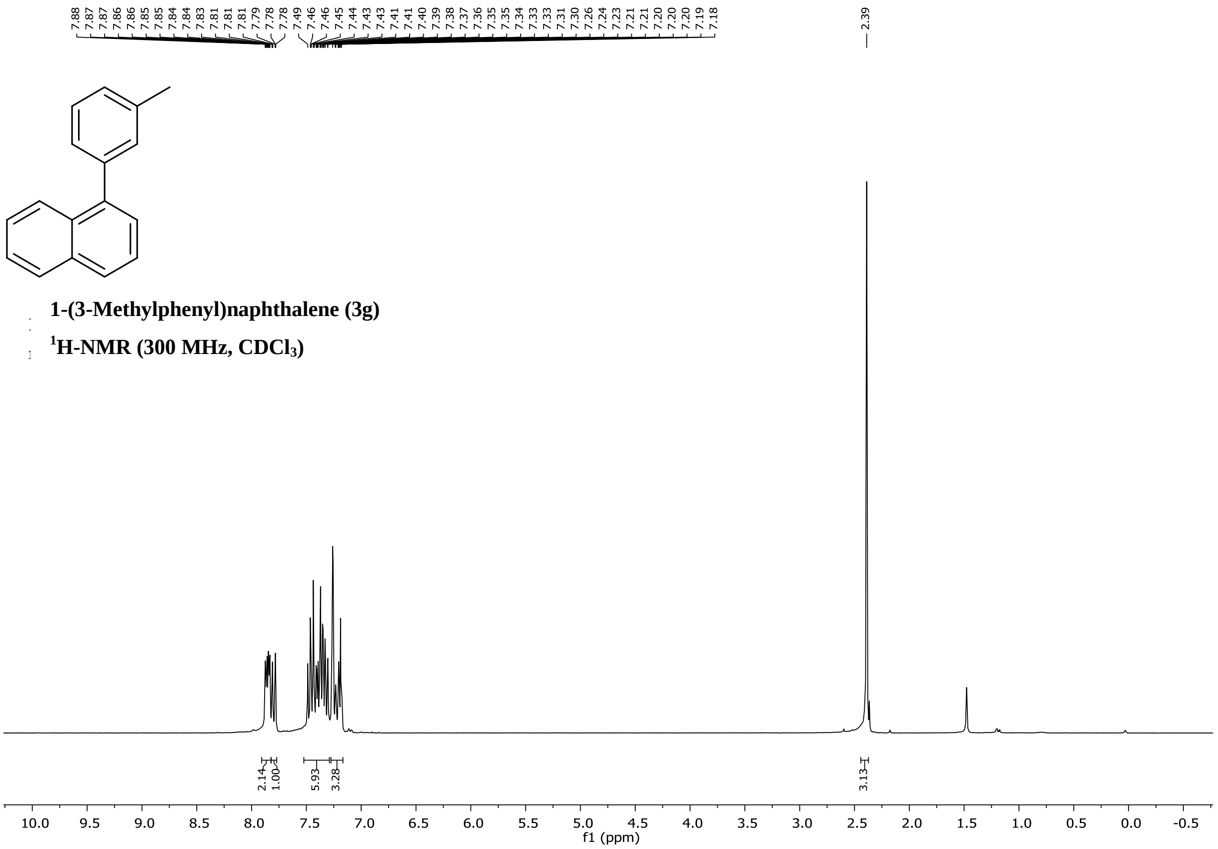
7.88 7.87 7.87 7.86 7.86 7.85 7.85 7.84 7.84 7.83 7.81 7.81 7.81 7.79 7.78 7.78 7.49 7.46 7.46 7.45 7.44 7.43 7.43 7.41 7.41 7.40 7.39 7.38 7.37 7.36 7.35 7.35 7.34 7.33 7.33 7.31 7.30 7.26 7.24 7.23 7.21 7.21 7.20 7.20 7.19 7.18
2.39
1-(3-Methylphenyl)naphthalene (3g)
1H-NMR (300 MHz, CDCl3)
2.14
1.00
5.93
3.28
3.13
10.0 9.5 9.0 8.5 8.0 7.5 7.0 6.5 6.0 5.5 5.0 4.5 4.0 3.5 3.0 2.5 2.0 1.5 1.0 0.5 0.0 -0.5
f1 (ppm)

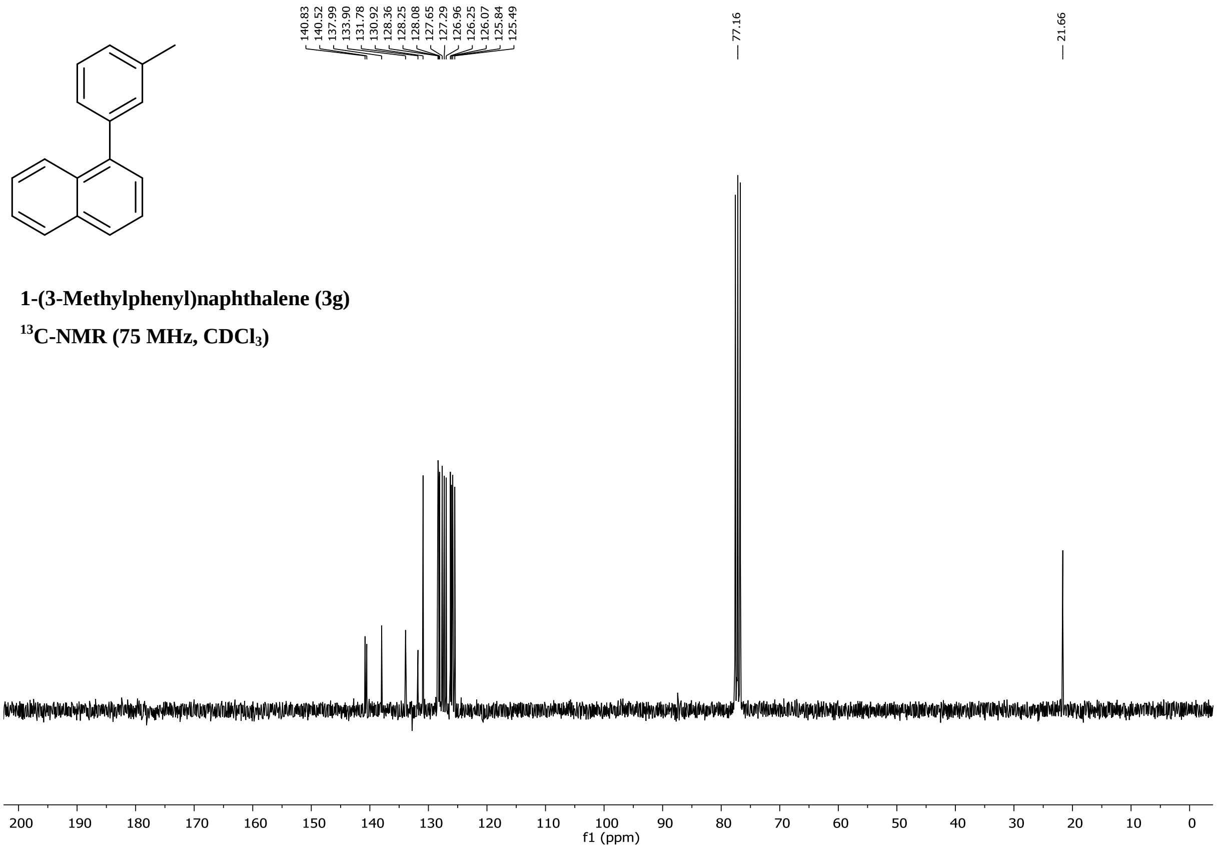
140.83 140.52 137.99 133.90 131.78 130.92 128.36 128.25 128.08 127.65 127.29 126.96 126.25 126.07 125.84 125.49
77.16
21.66
1-(3-Methylphenyl)naphthalene (3g)
13C-NMR (75 MHz, CDCl3)
200 190 180 170 160 150 140 130 120 110 100 90 80 70 60 50 40 30 20 10 0
f1 (ppm)

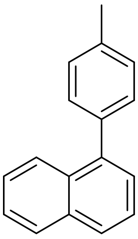

1-(4-Methylphenyl)naphthalene (3i)

^{1}H-NMR (400 MHz, $CDCl_3$)

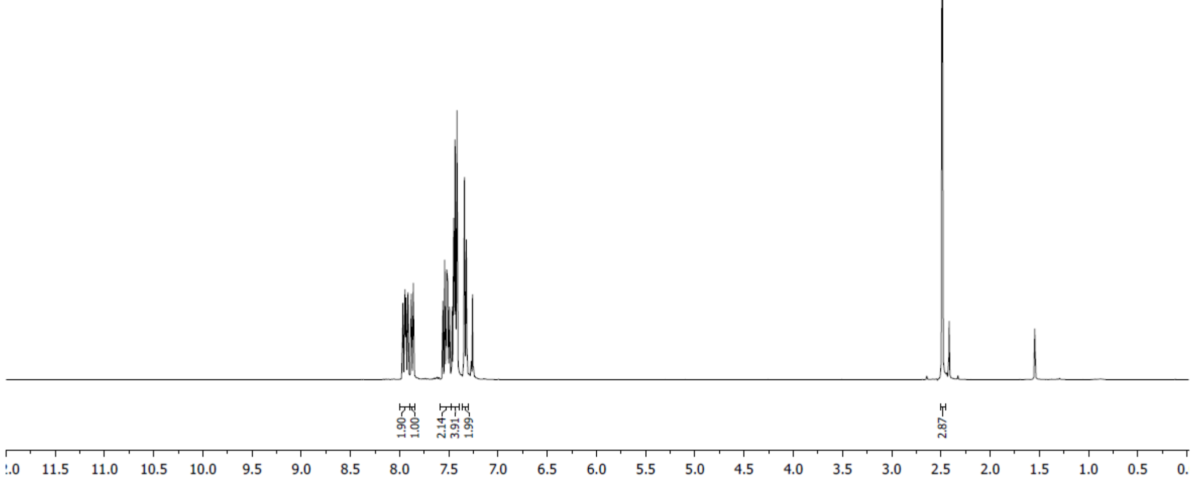

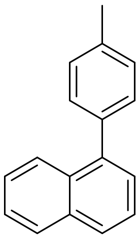

1-(4-Methylphenyl)naphthalene (3i)

^{13}C-NMR (101 MHz, $CDCl_3$)

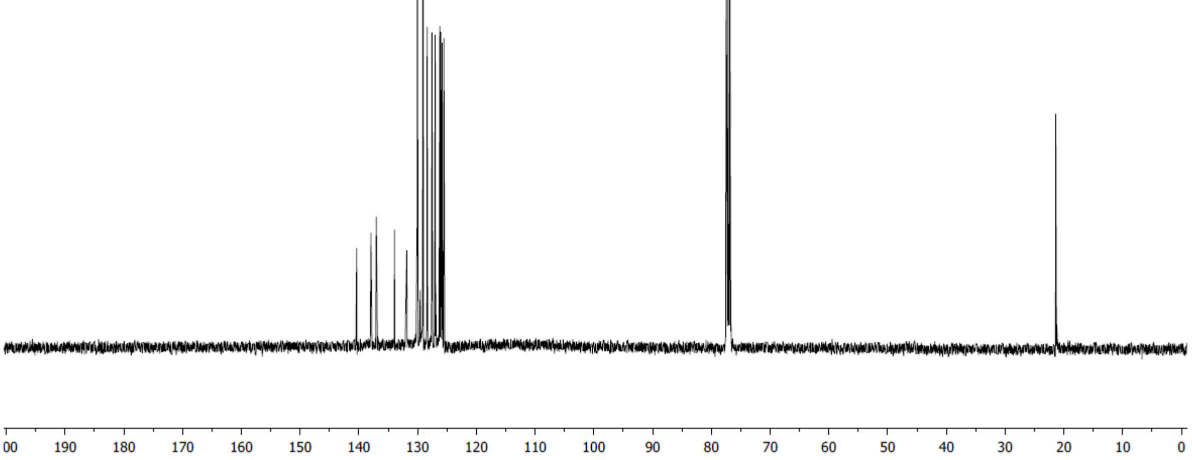

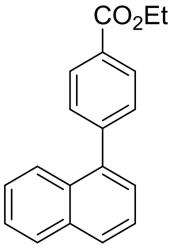

1-(4-Ethoxycarbonylphenyl)naphthalene (3j)

^{1}H-NMR (400 MHz, $CDCl_3$)

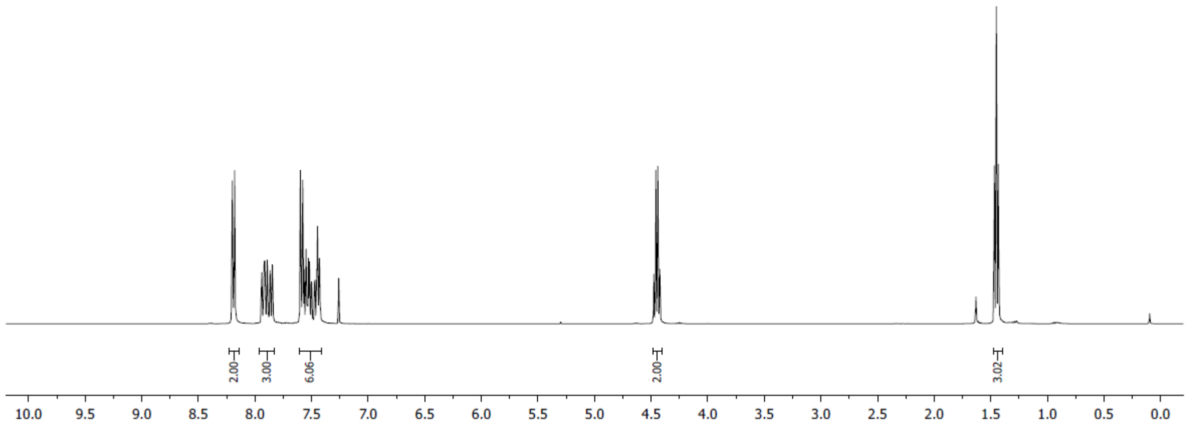

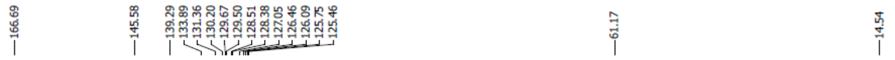

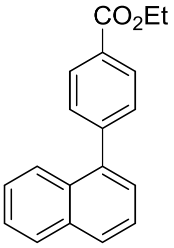

1-(4-Ethoxycarbonylphenyl)naphthalene (3j)

^{13}C-NMR (101 MHz, $CDCl_3$)

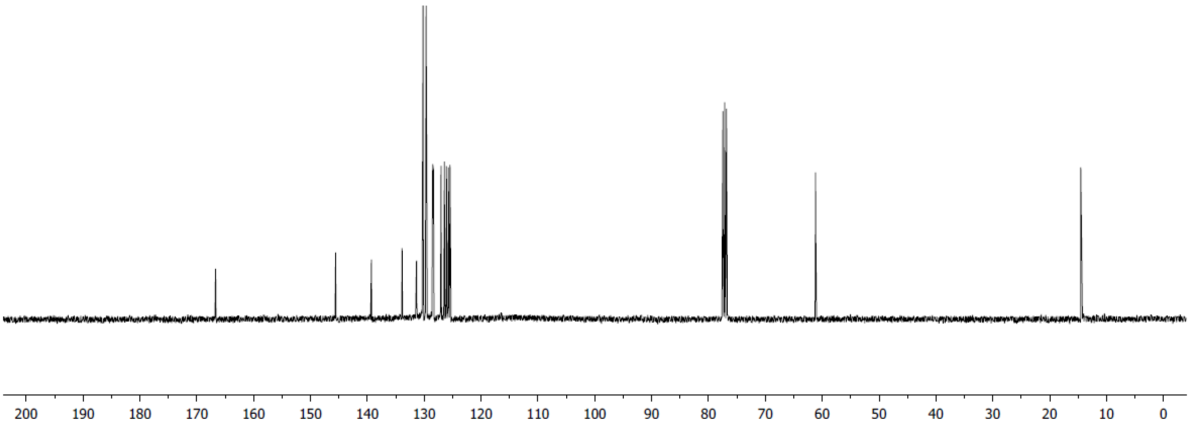

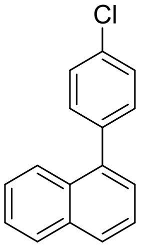

1-(4-Chlorophenyl)naphthalene (3k)

^{1}H-NMR (400 MHz, CDCl$_3$)

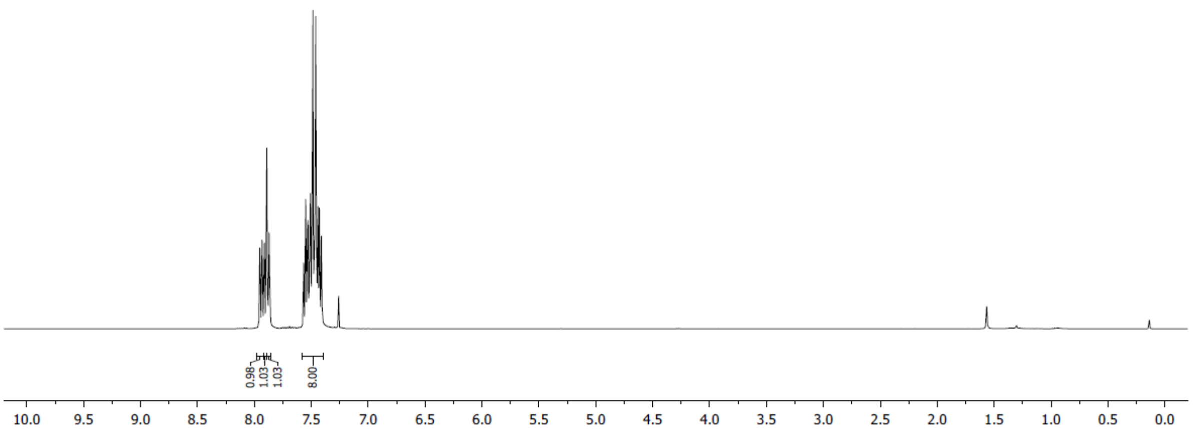

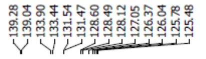

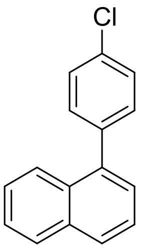

1-(4-Chlorophenyl)naphthalene (3k)

^{13}C-NMR (101 MHz, CDCl$_3$)

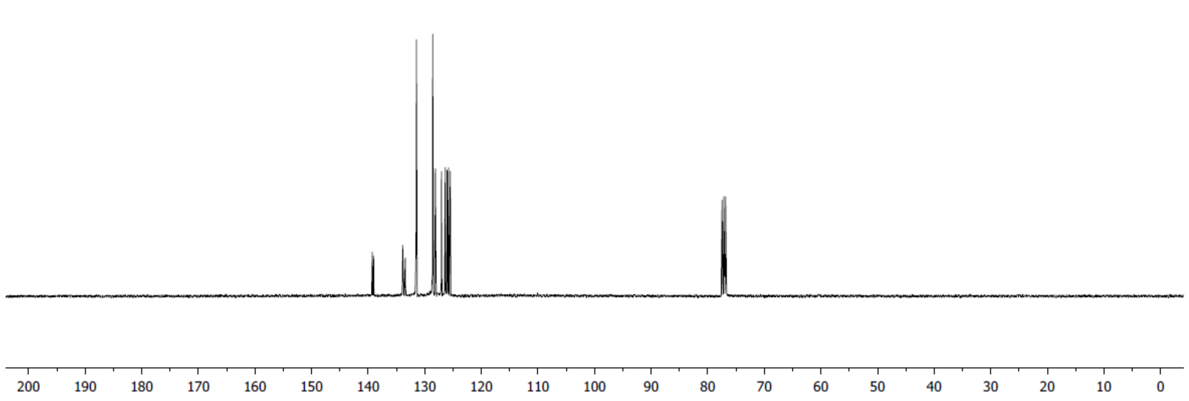

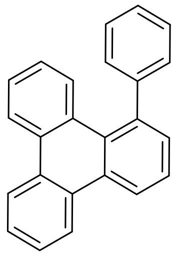

1-Phenyltriphenylene (5a)

^{1}H-NMR (300 MHz, $CDCl_3$)

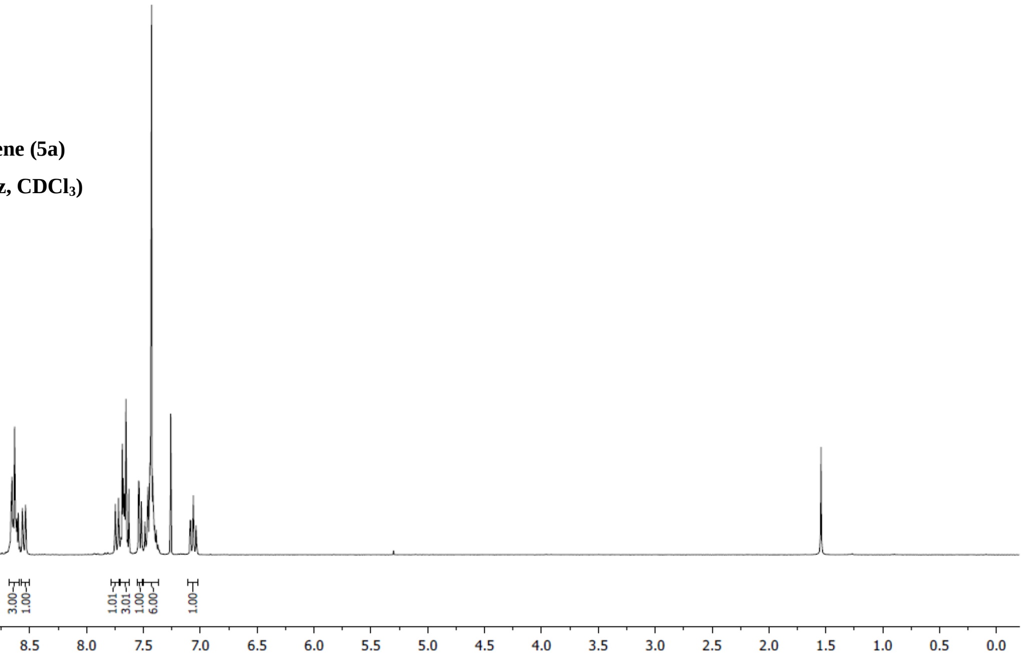

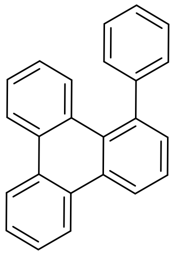

1-Phenyltriphenylene (5b)

^{13}C-NMR (101 MHz, $CDCl_3$)

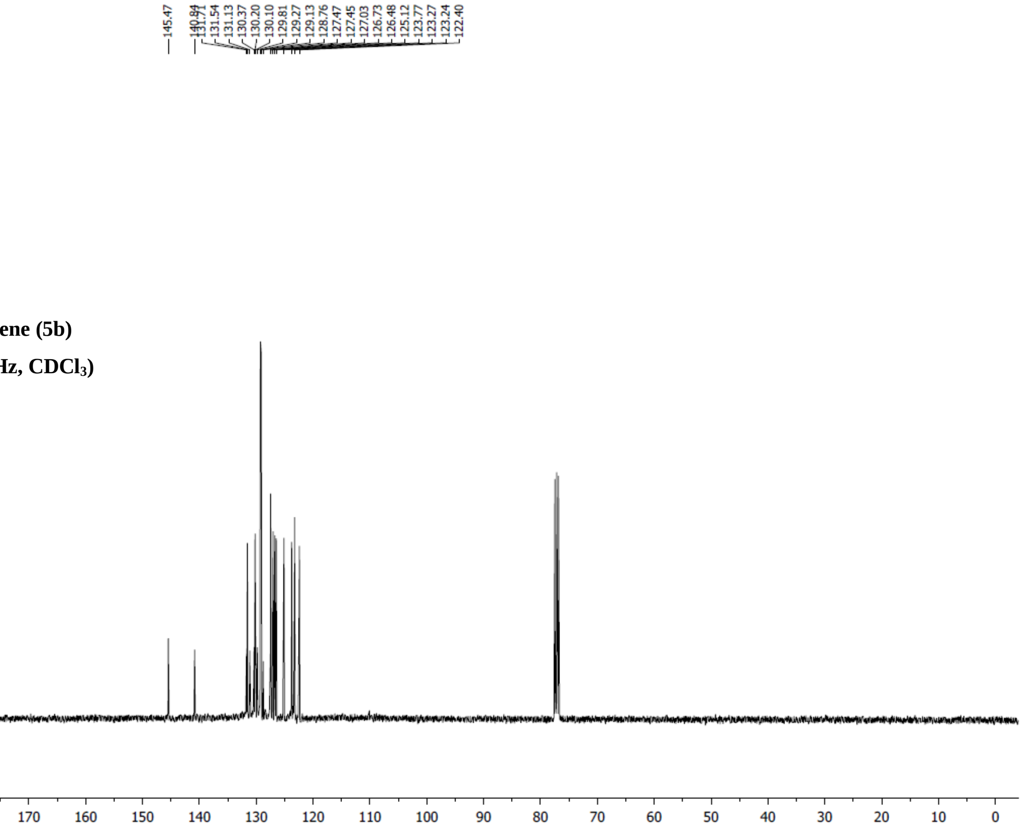

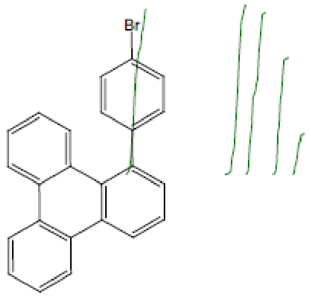

1-(4-Bromophenyl)triphenylene (5b)

^{1}H-NMR (300 MHz, $CDCl_3$)

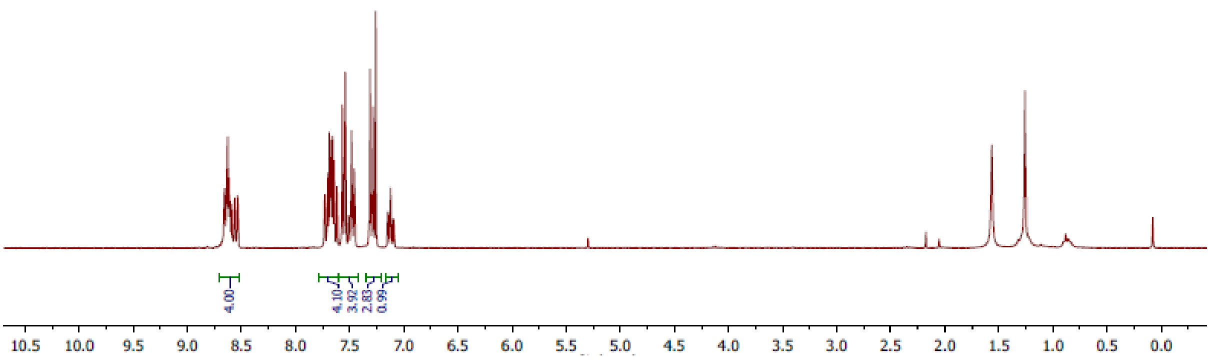

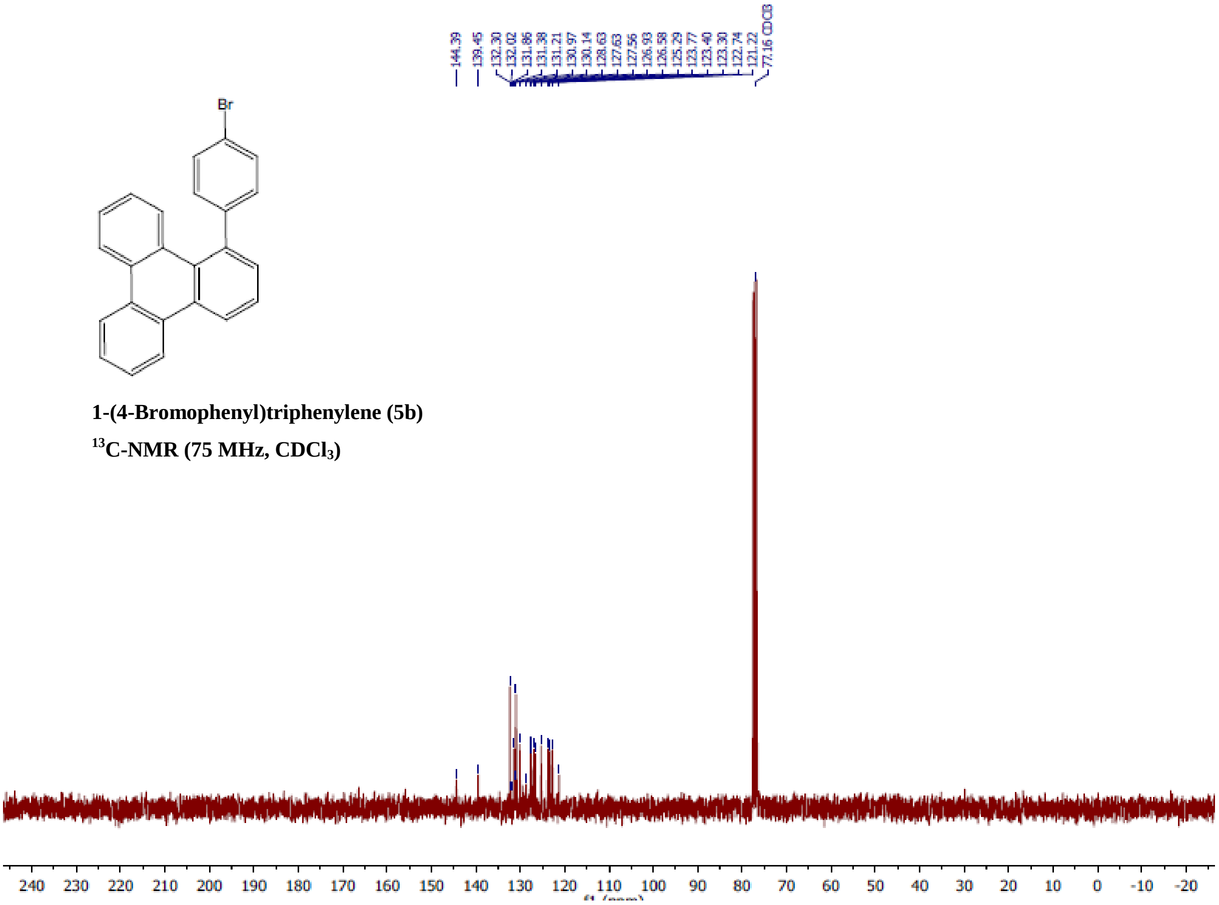

1-(4-Bromophenyl)triphenylene (5b)

^{13}C-NMR (75 MHz, $CDCl_3$)

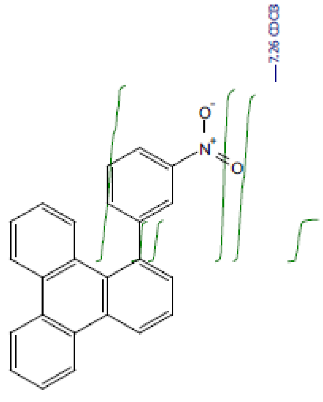

1-(3-Nitrophenyl)triphenylene (5c)

^{13}C-NMR (101 MHz, $CDCl_3$)

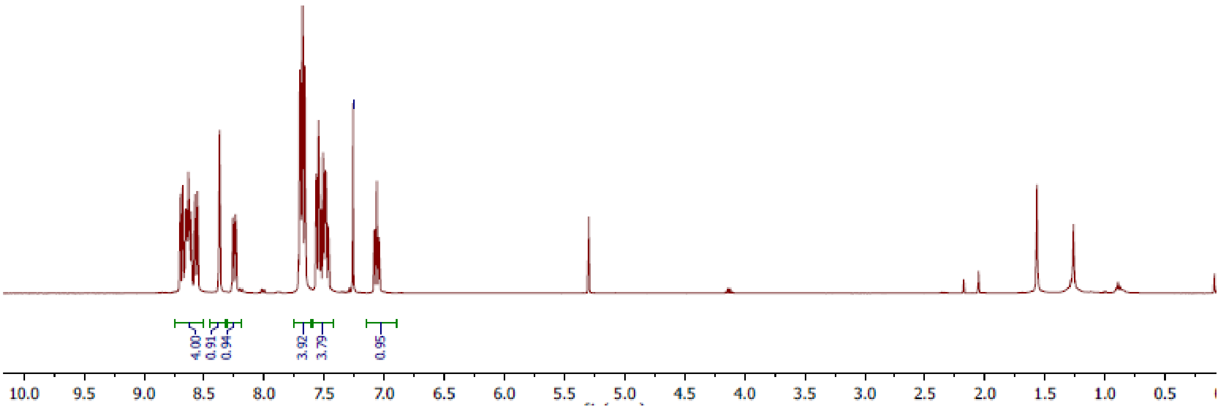

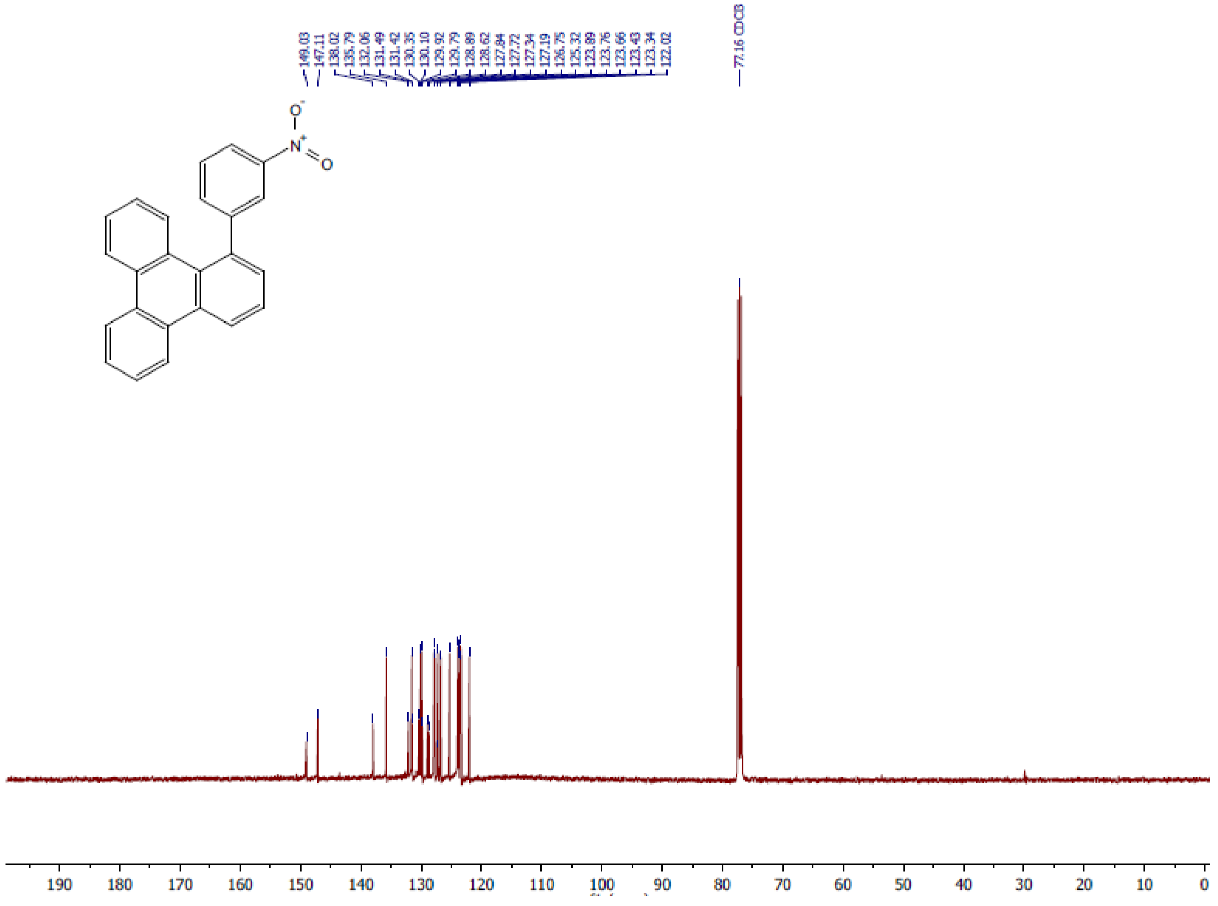

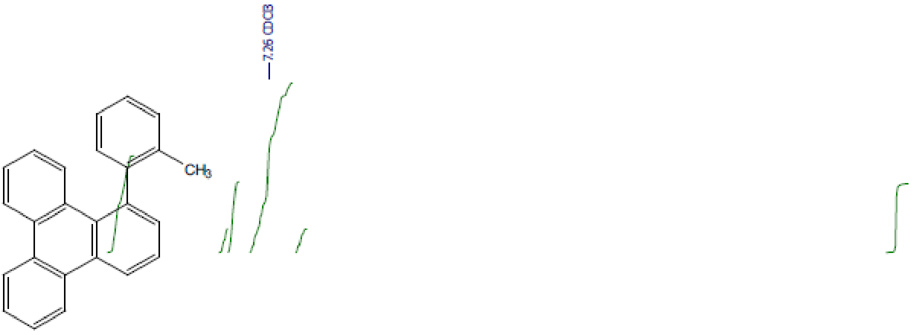

1-(2-Methylphenyl)triphenylene (5d)

^{1}H-NMR (300 MHz, CDCl$_3$)

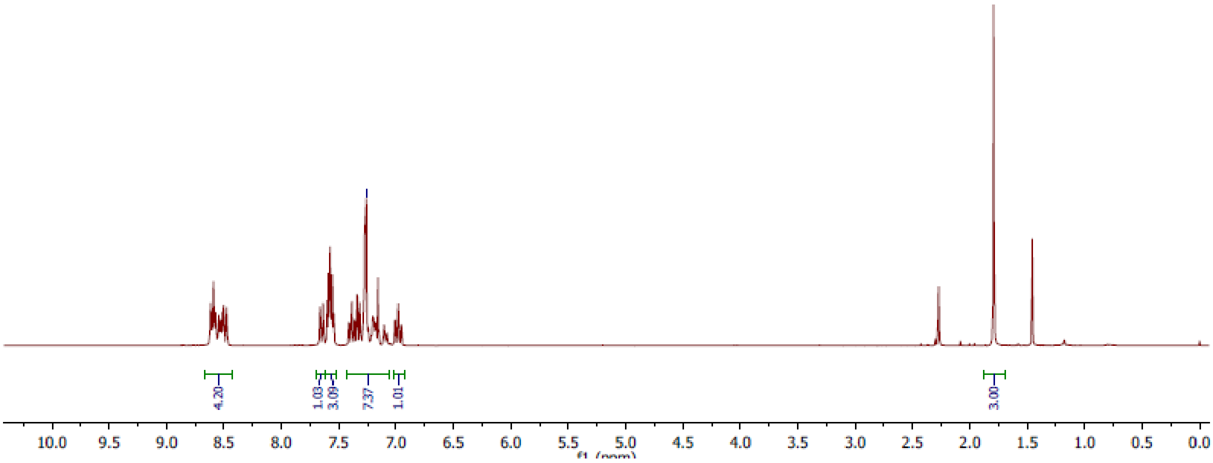

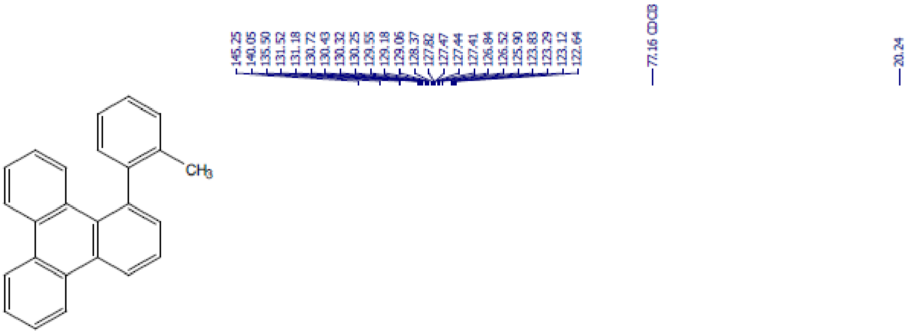

1-(2-Methylphenyl)triphenylene (5d)

^{13}C-NMR (75 MHz, CDCl$_3$)

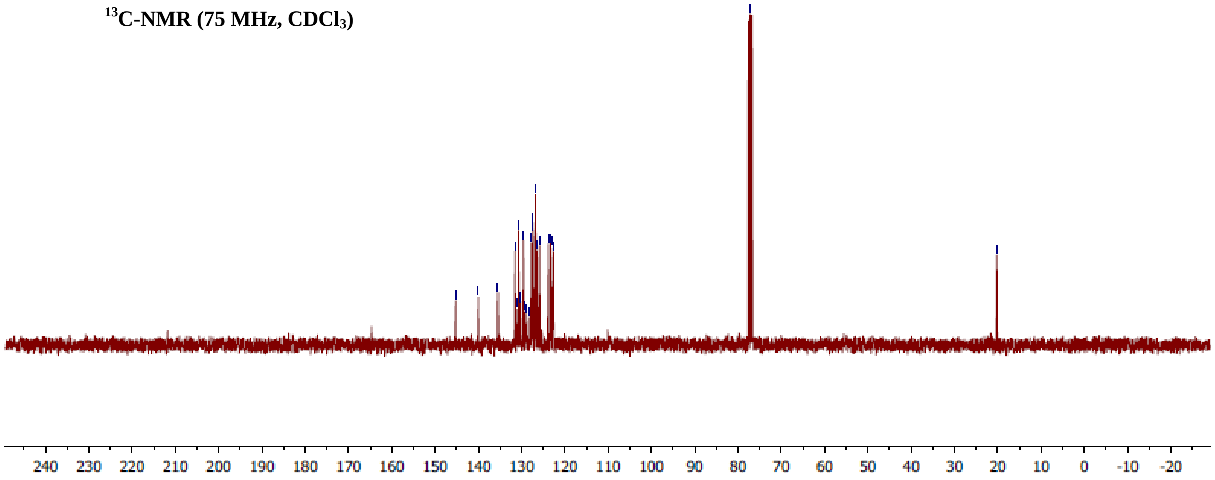

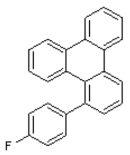

1-(4-Fluorophenyl)triphenylene (5e)

^{13}C-NMR (75 MHz, $CDCl_3$)

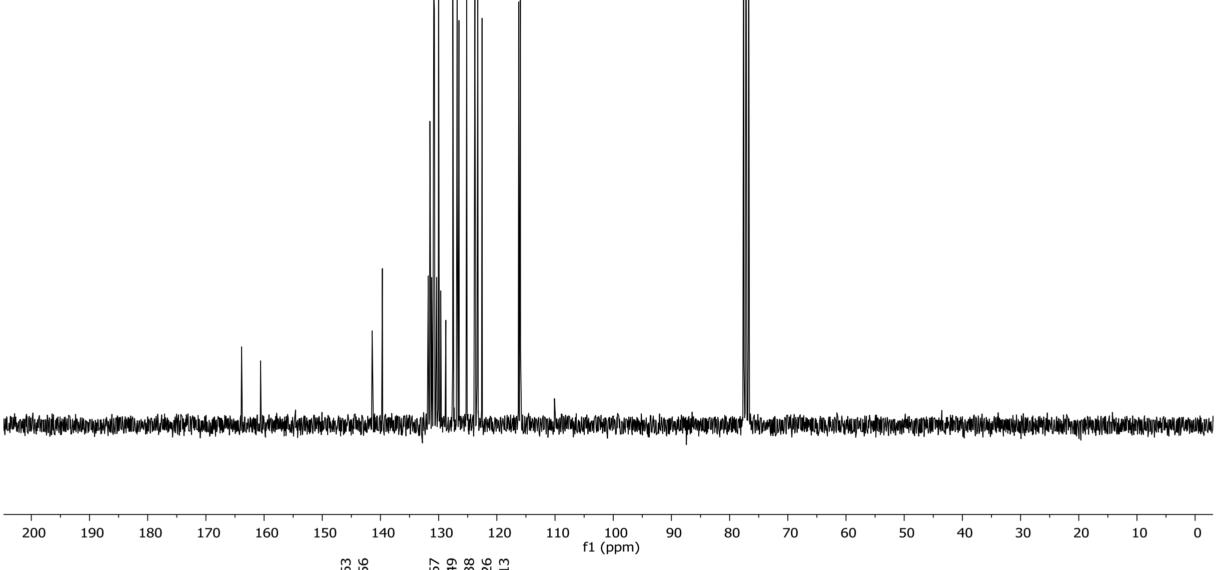

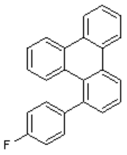

1-(4-Fluorophenyl)triphenylene (5e)

^{1}H-NMR (300 MHz, $CDCl_3$)

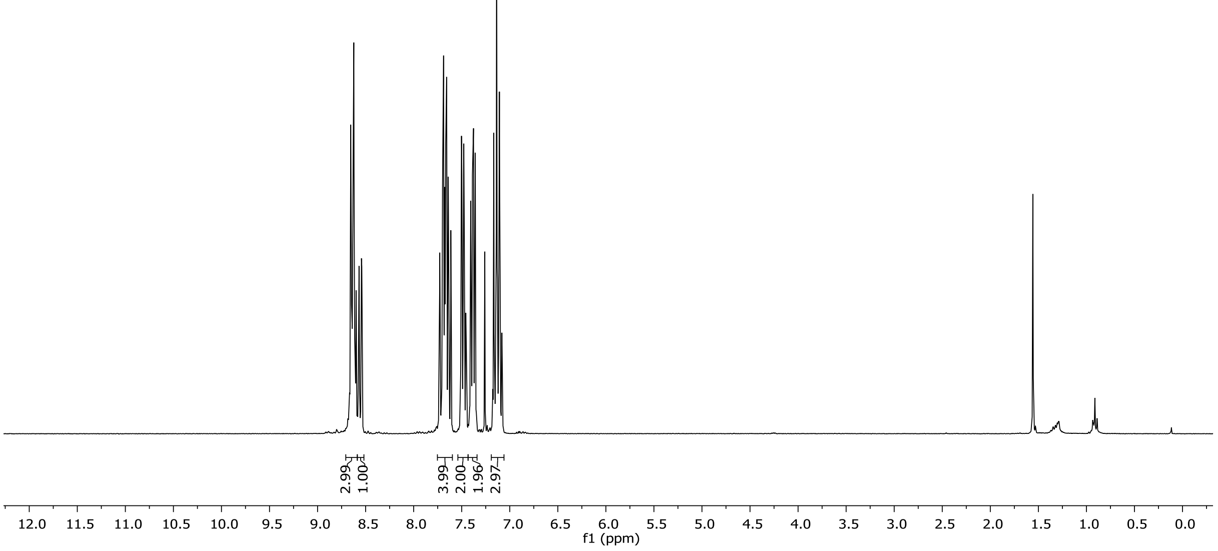

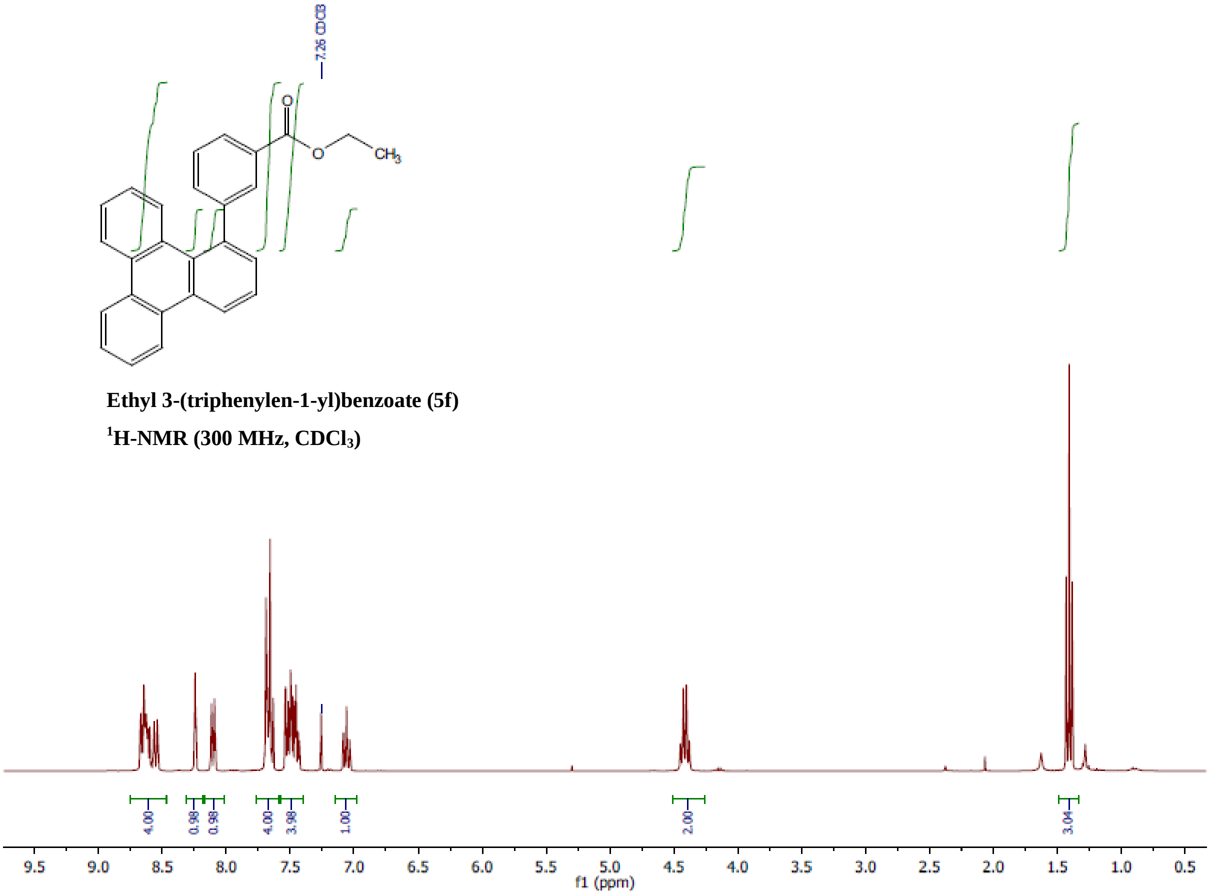

Ethyl 3-(triphenylen-1-yl)benzoate (5f)

^{1}H-NMR (300 MHz, $CDCl_3$)

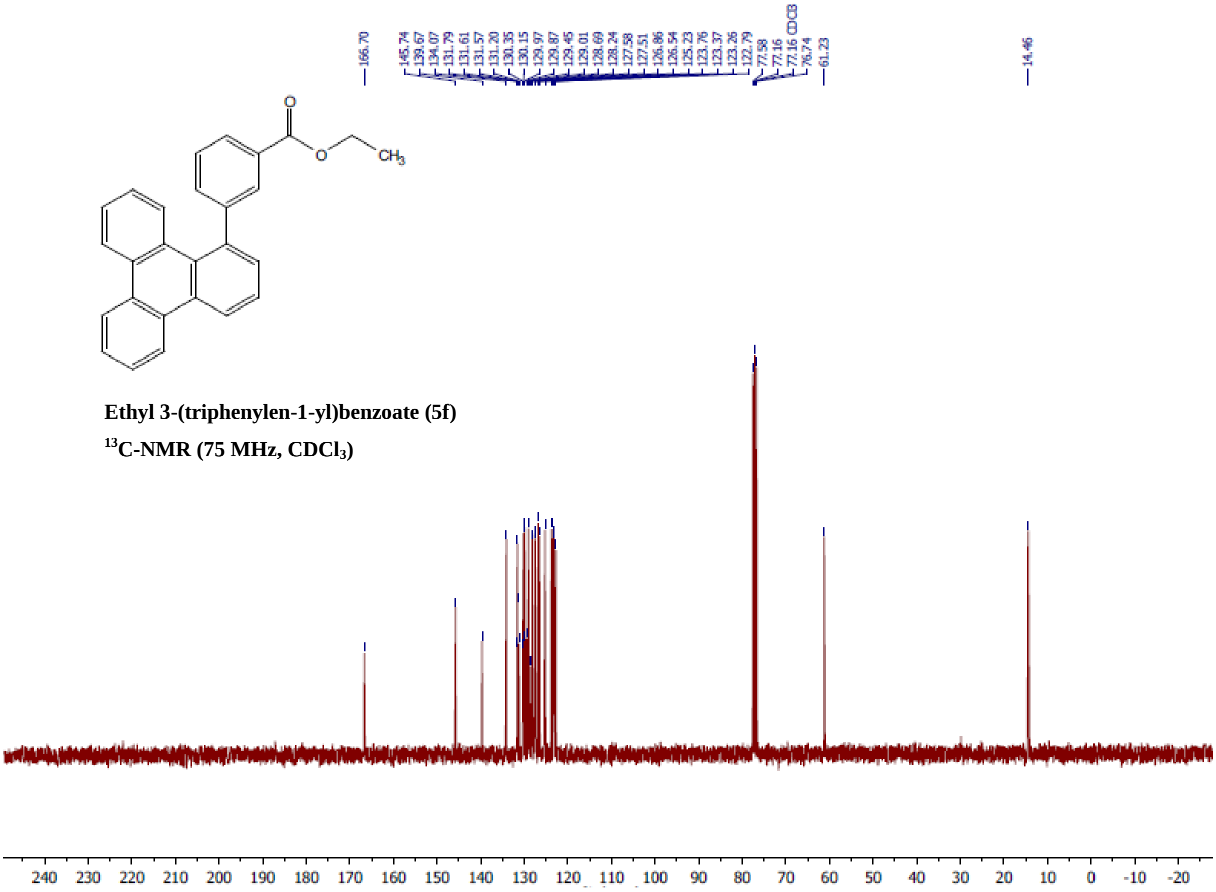

Ethyl 3-(triphenylen-1-yl)benzoate (5f)

^{13}C-NMR (75 MHz, $CDCl_3$)

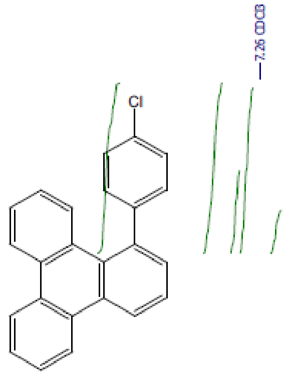

1-(4-Chlorophenyl)triphenylene (5g)

^{1}H-NMR (300 MHz, $CDCl_3$)

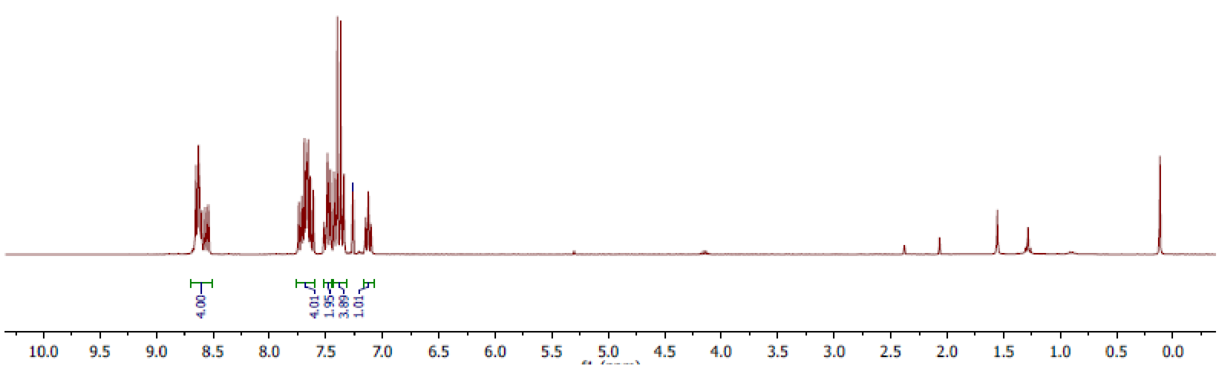

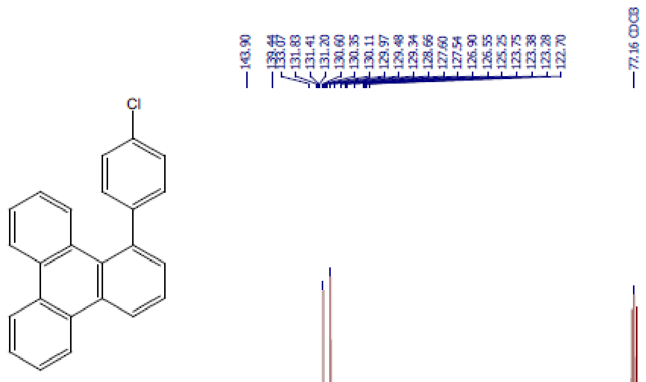

1-(4-Chlorophenyl)triphenylene (5g)

^{13}C-NMR (101 MHz, $CDCl_3$)

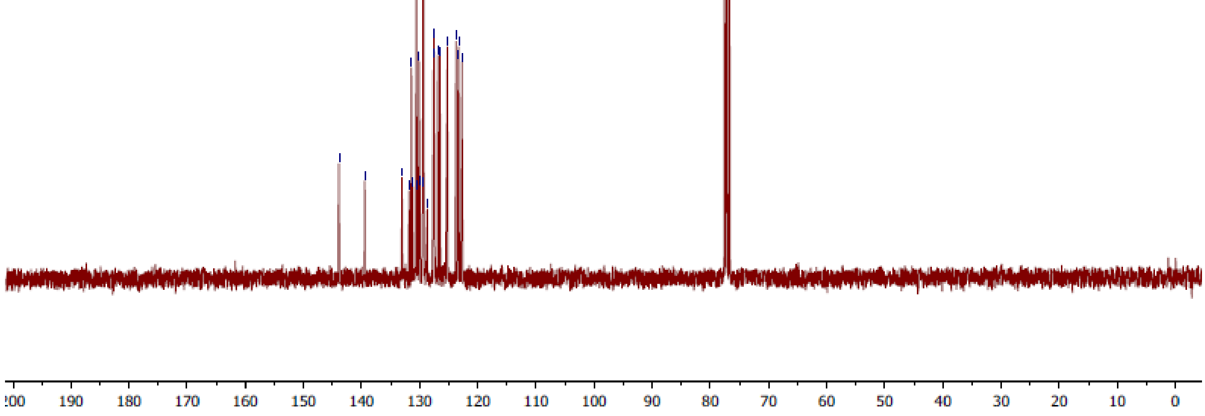

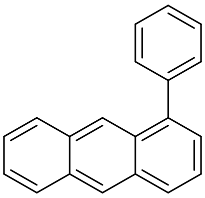

1-Phenylanthracene (6a)

^{1}H-NMR (400 MHz, CDCl$_3$)

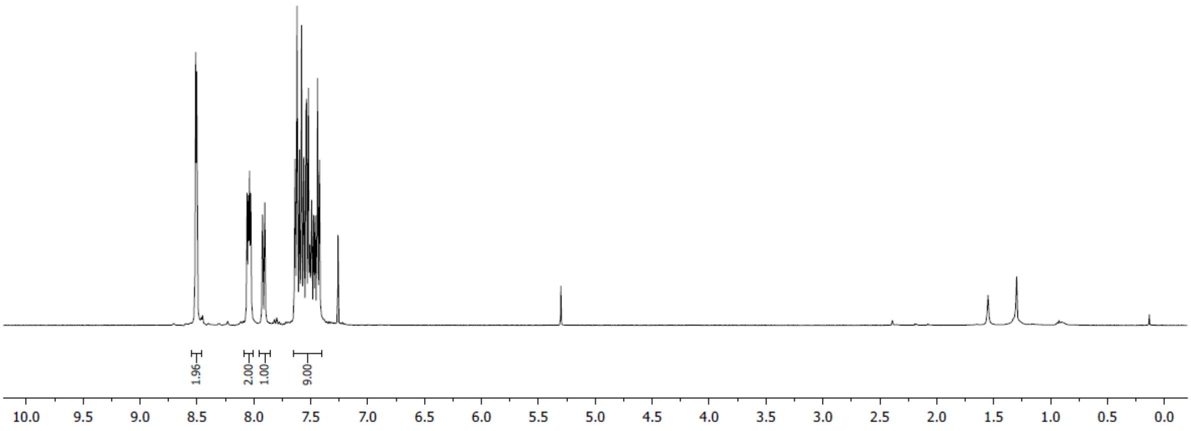

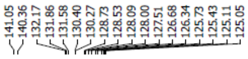

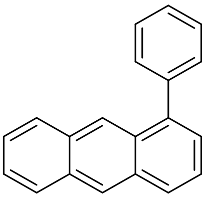

1-Phenylanthracene (6a)

^{13}C-NMR (101 MHz, CDCl$_3$)

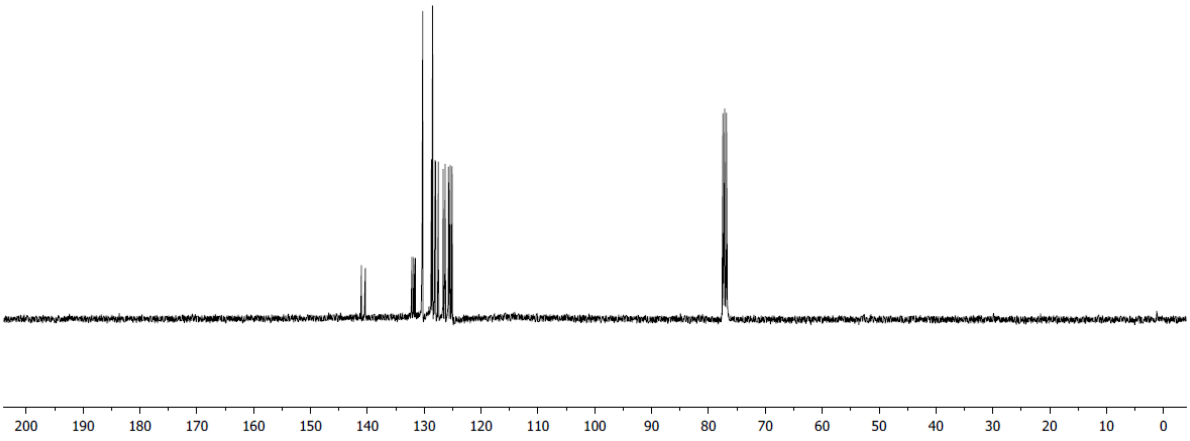

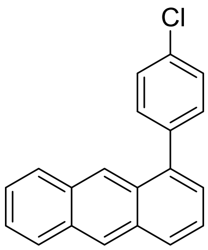

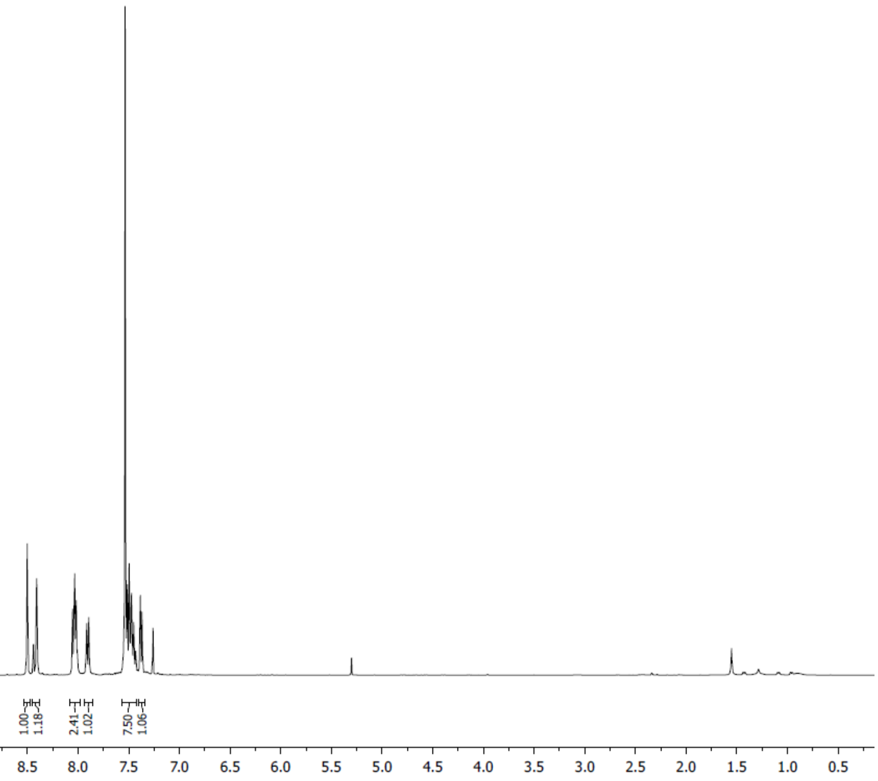

1-(4-Chlorophenyl)anthracene (6b)

^{1}H-NMR (300 MHz, $CDCl_3$)

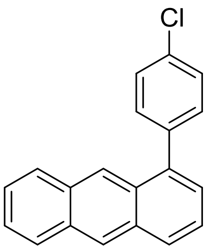

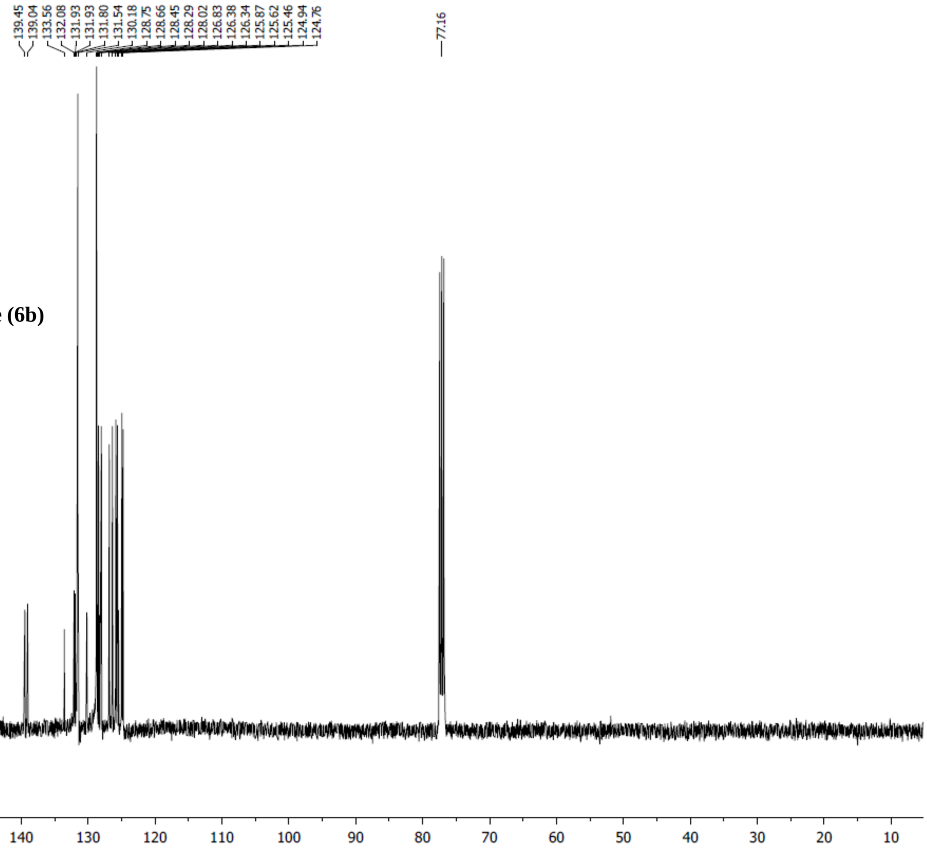

1-(4-Chlorophenyl)anthracene (6b)

^{13}C-NMR (101 MHz, $CDCl_3$)

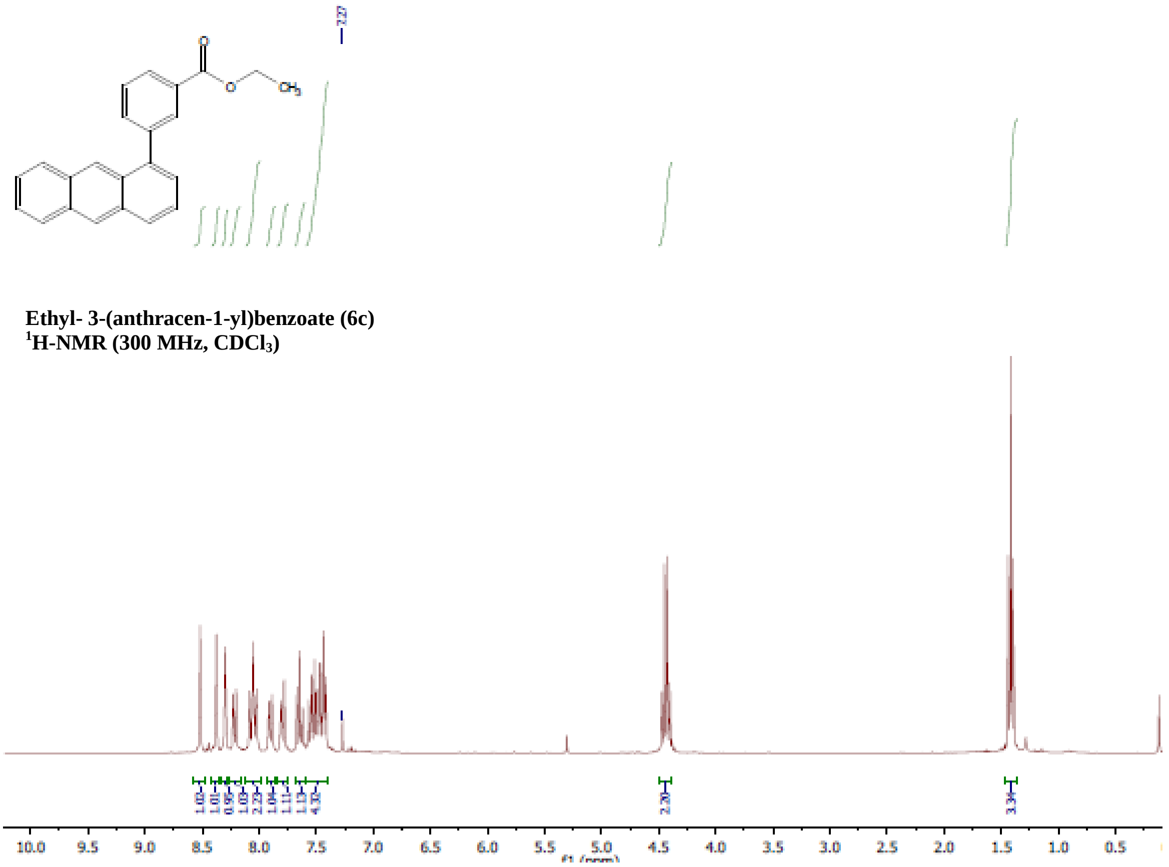

Ethyl- 3-(anthracen-1-yl)benzoate (6c)
^{1}H-NMR (300 MHz, CDCl$_3$)

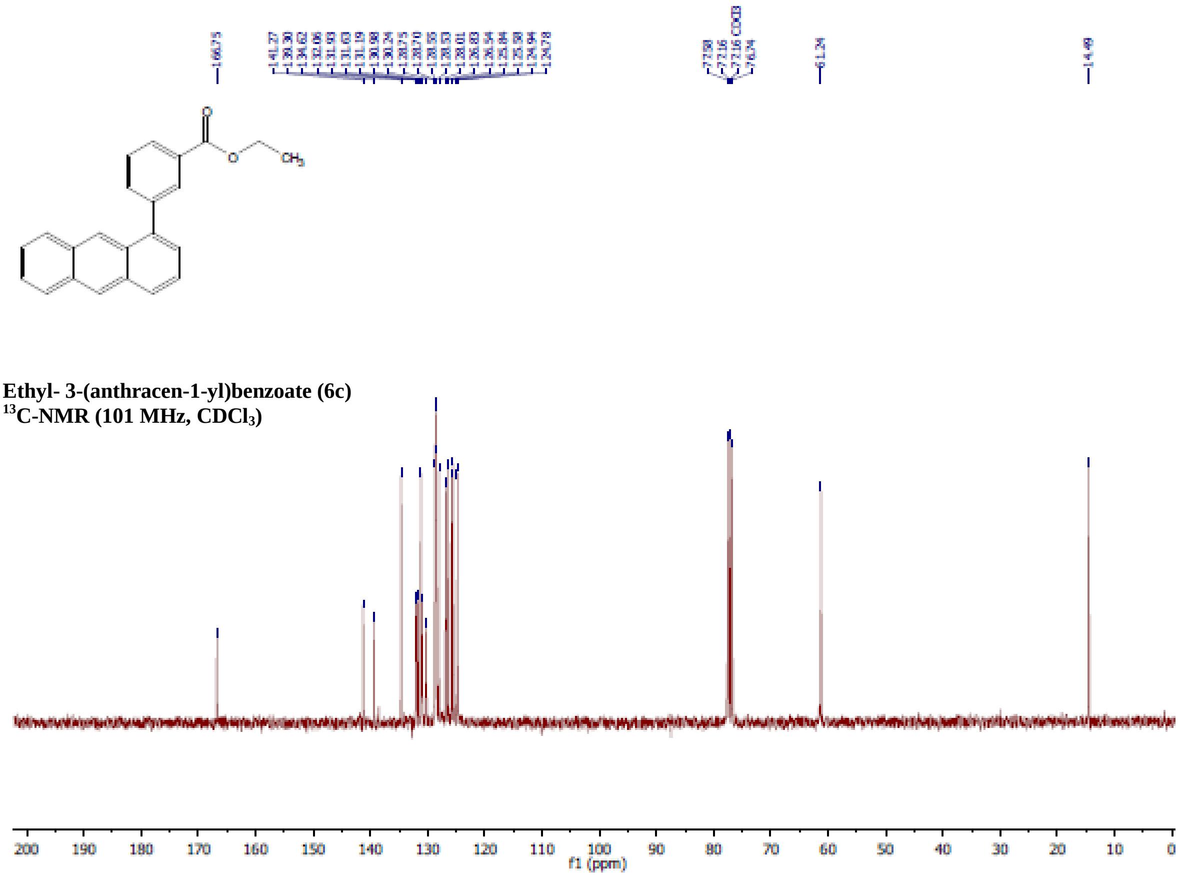

Ethyl- 3-(anthracen-1-yl)benzoate (6c)
^{13}C-NMR (101 MHz, CDCl$_3$)

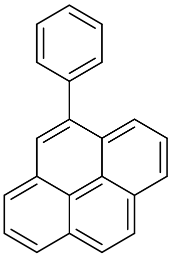

4-Phenylpyrene (7a)

^{1}H-NMR (300 MHz, $CDCl_3$)

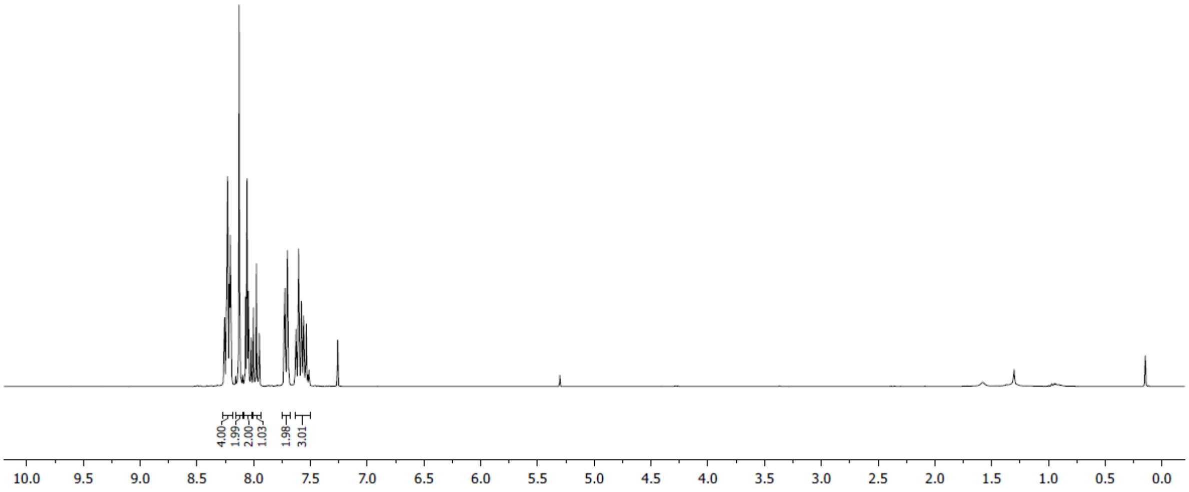

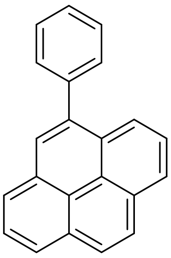

4-Phenylpyrene (7a)

^{13}C-NMR (75 MHz, $CDCl_3$)

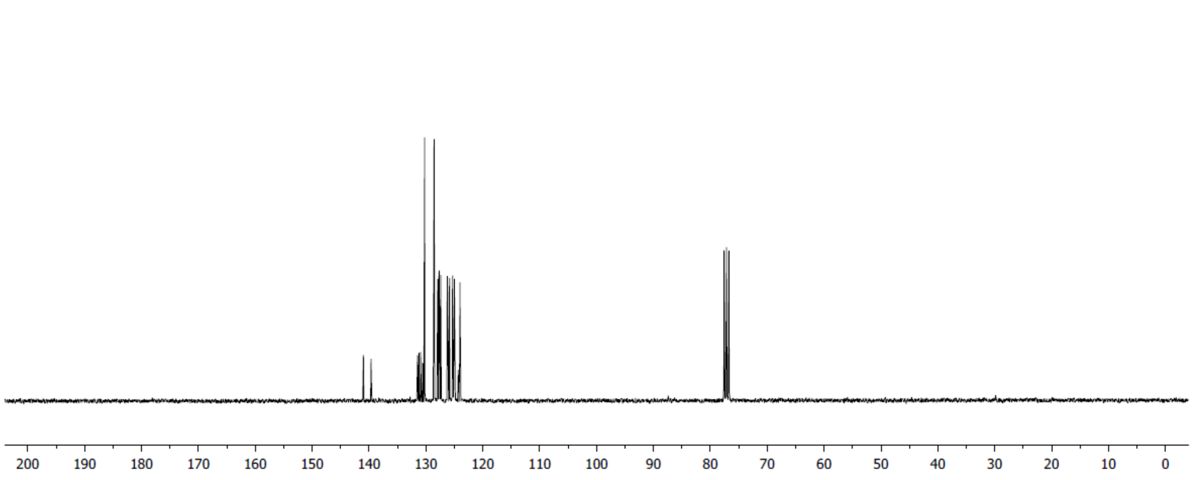

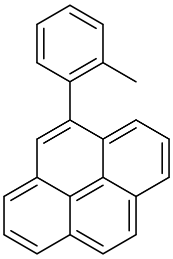

4-(2-Methylphenyl)pyrene (7b)

^{1}H-NMR (300 MHz, $CDCl_3$)

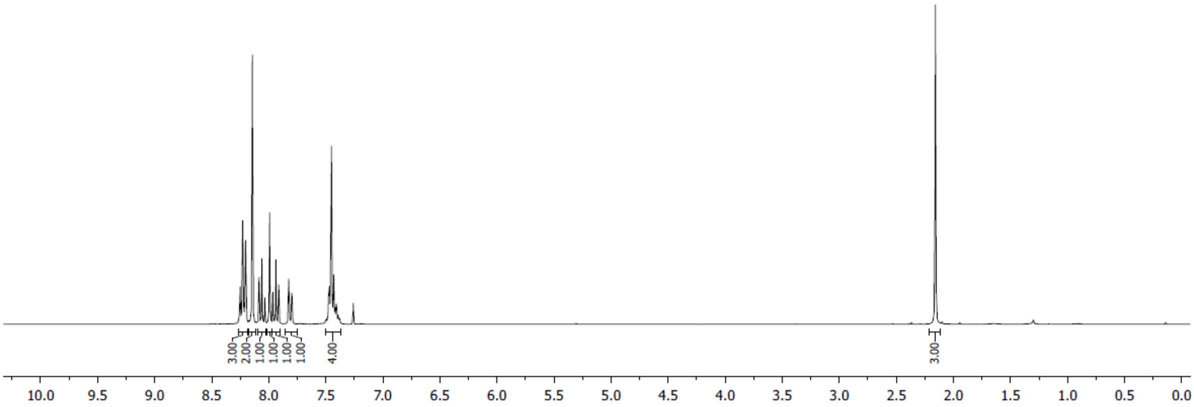

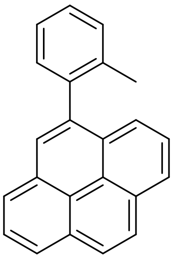

4-(2-Methylphenyl)pyrene (7b)

^{13}C-NMR (75 MHz, $CDCl_3$)

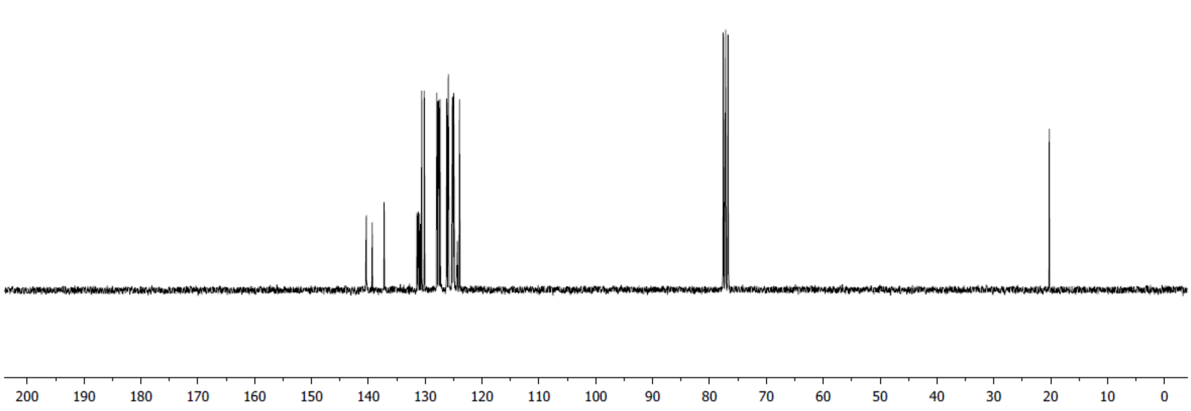

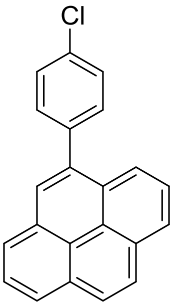

4-(4-Chlorophenyl)pyrene (7c)

^{1}H-NMR (400 MHz, $CDCl_3$)

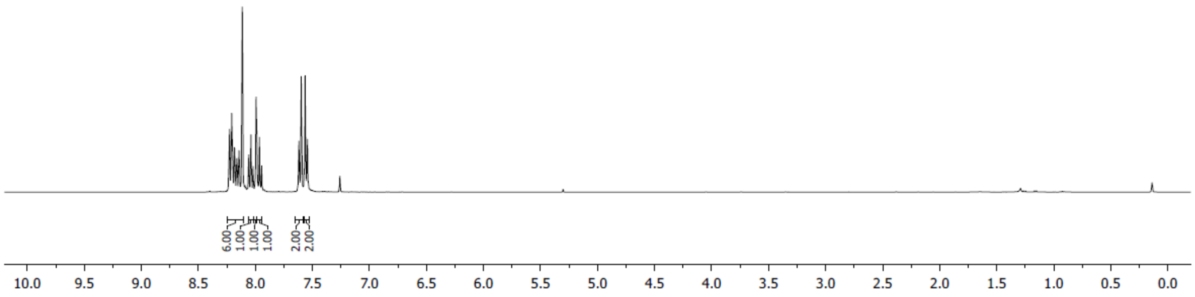

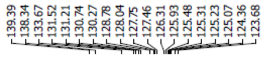

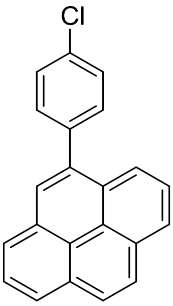

4-(4-Chlorophenyl)pyrene (7c)

^{13}C-NMR (100 MHz, $CDCl_3$)

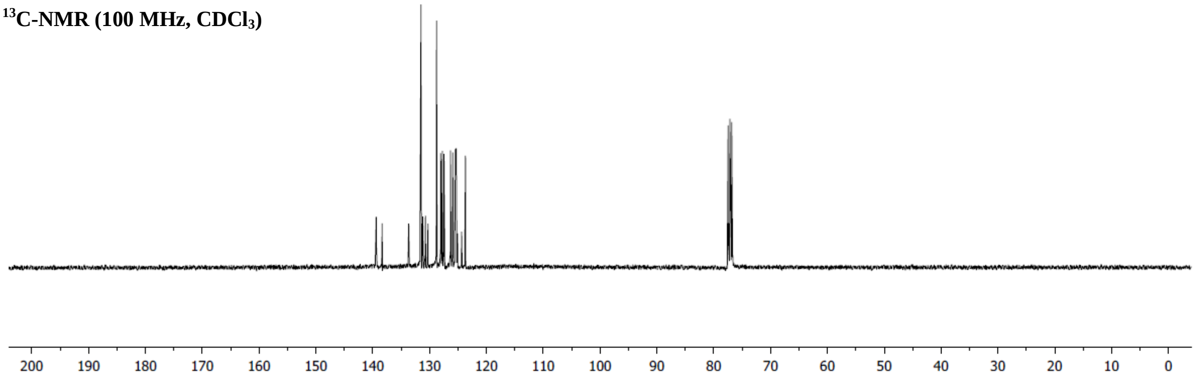

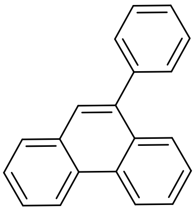

9-Phenylphenanthrene (8) + two regioisomers

^{1}H-NMR (300 MHz, $CDCl_3$)

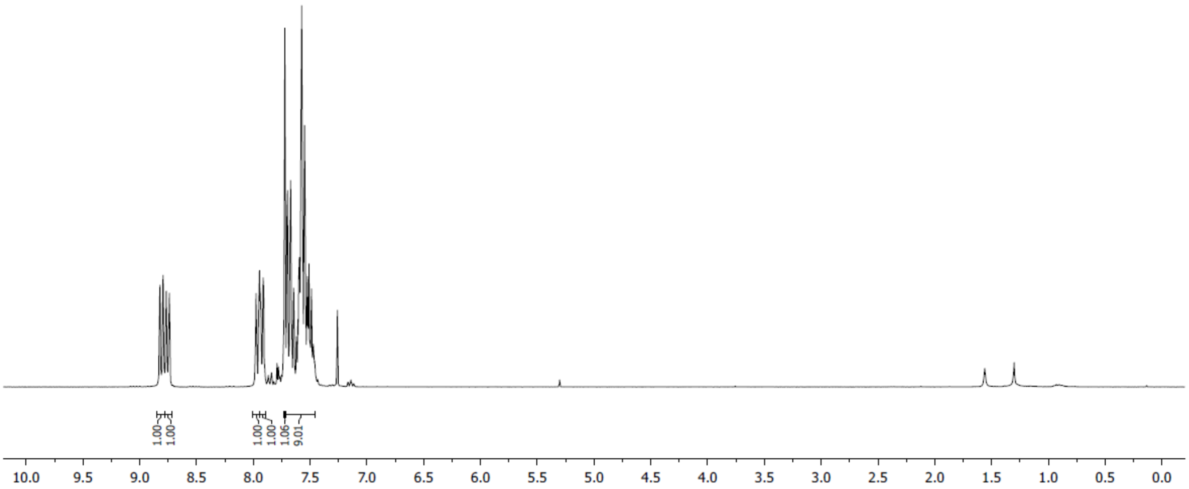

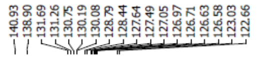

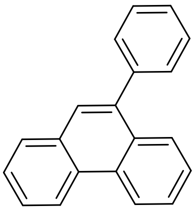

9-Phenylphenanthrene (8) + two regioisomers

^{13}C-NMR (75 MHz, $CDCl_3$)

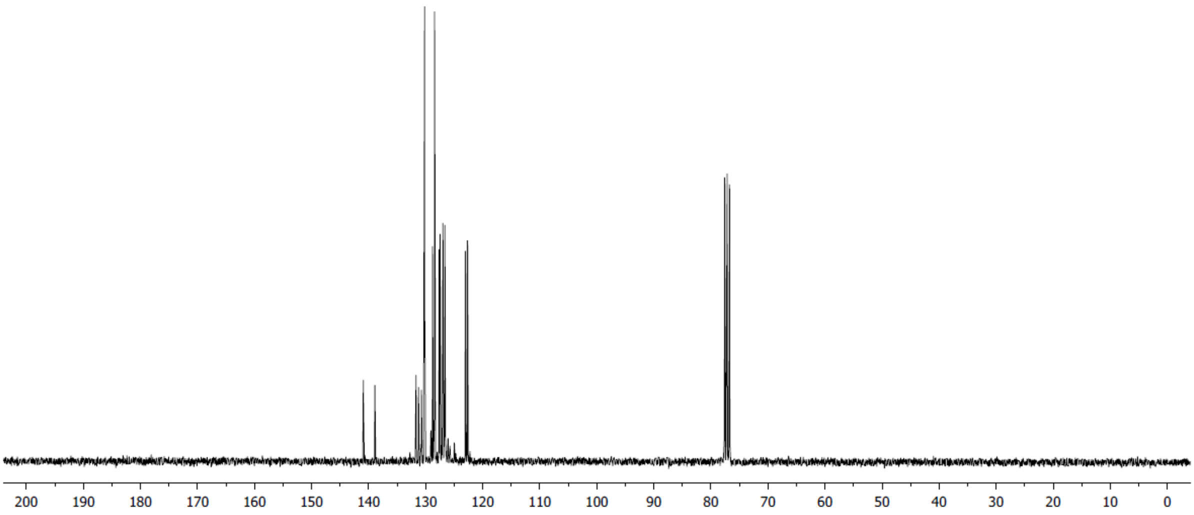

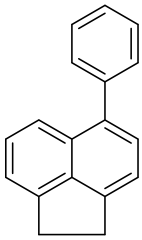

5-Phenylacenaphthene (9a)

^{1}H-NMR (300 MHz, CDCl$_3$)

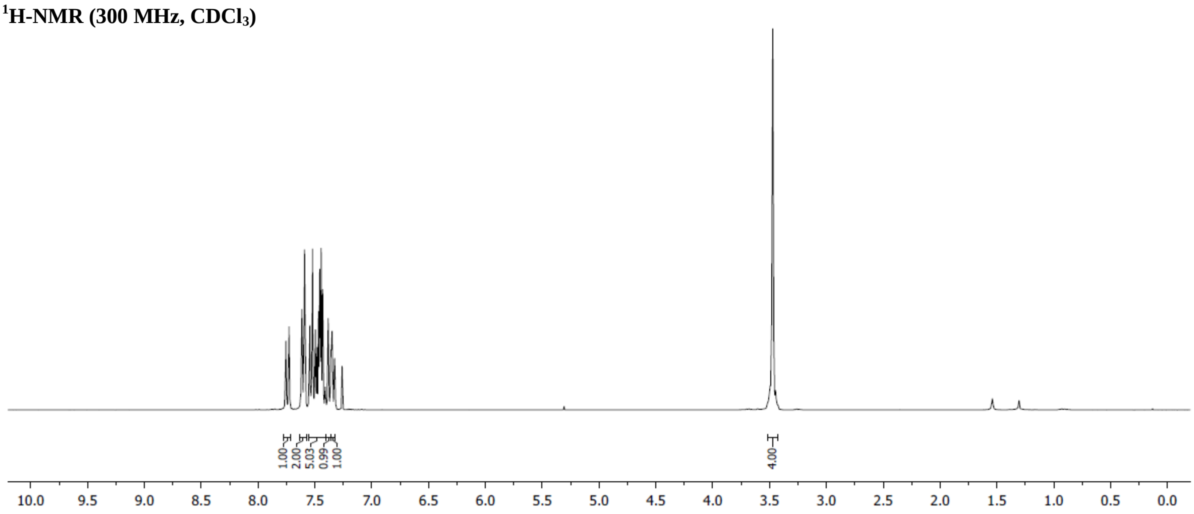

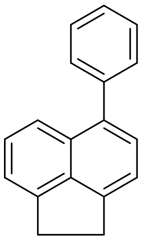

5-Phenylacenaphthene (9a)

^{13}C-NMR (75 MHz, CDCl$_3$)

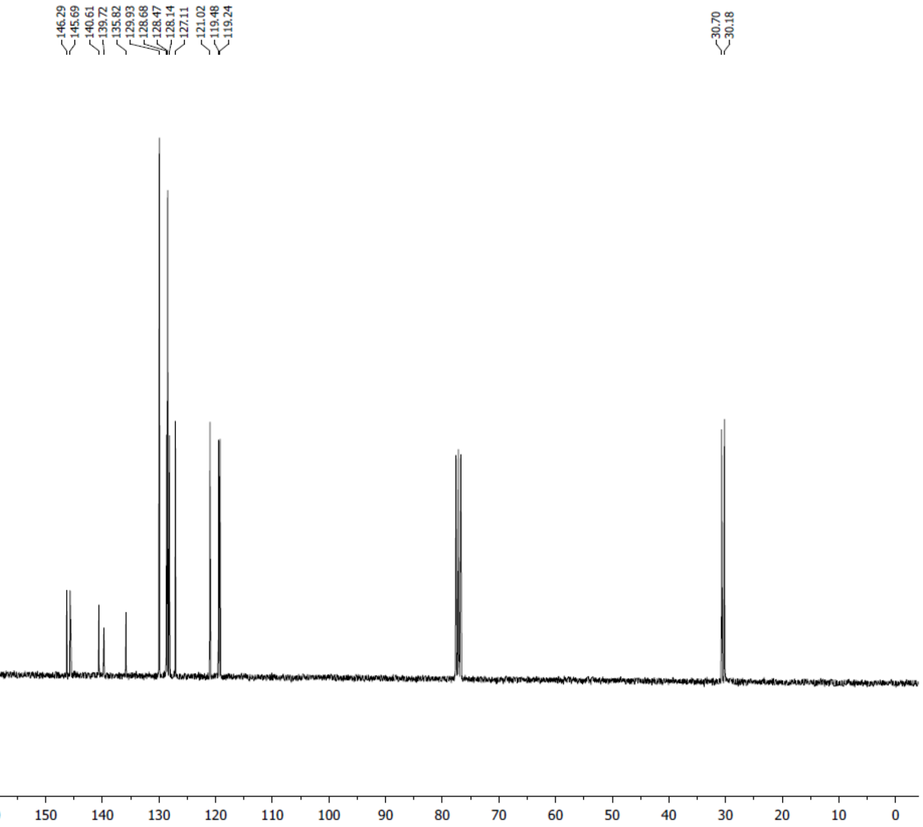

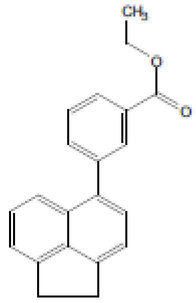

Ethyl- 3-(1,2-dihydroacenaphthylen-5-yl)benzoate (9b)

^{1}H-NMR (300 MHz, $CDCl_3$)

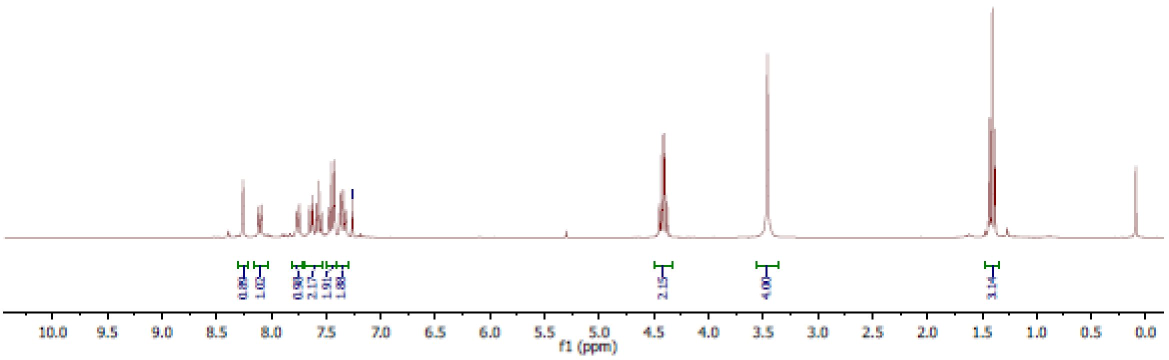

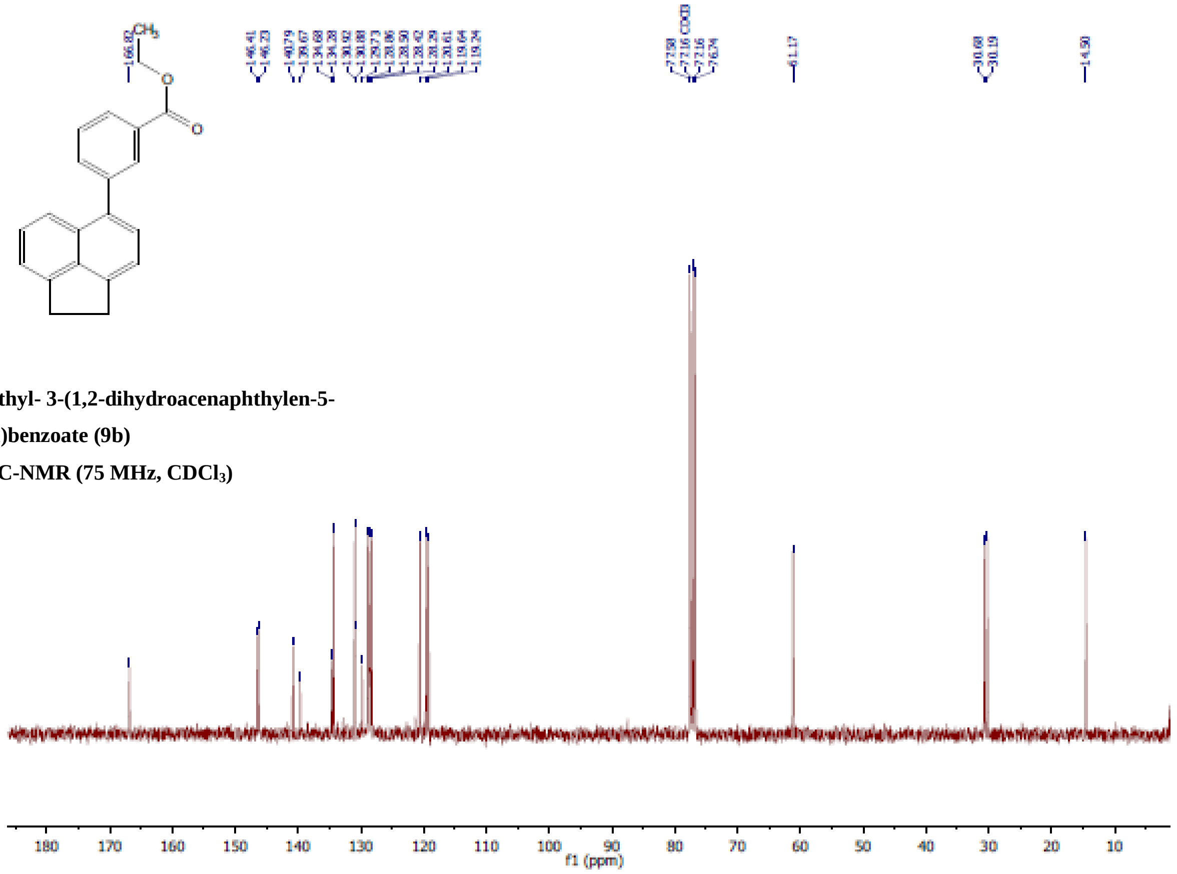

Ethyl- 3-(1,2-dihydroacenaphthylen-5-yl)benzoate (9b)

^{13}C-NMR (75 MHz, $CDCl_3$)

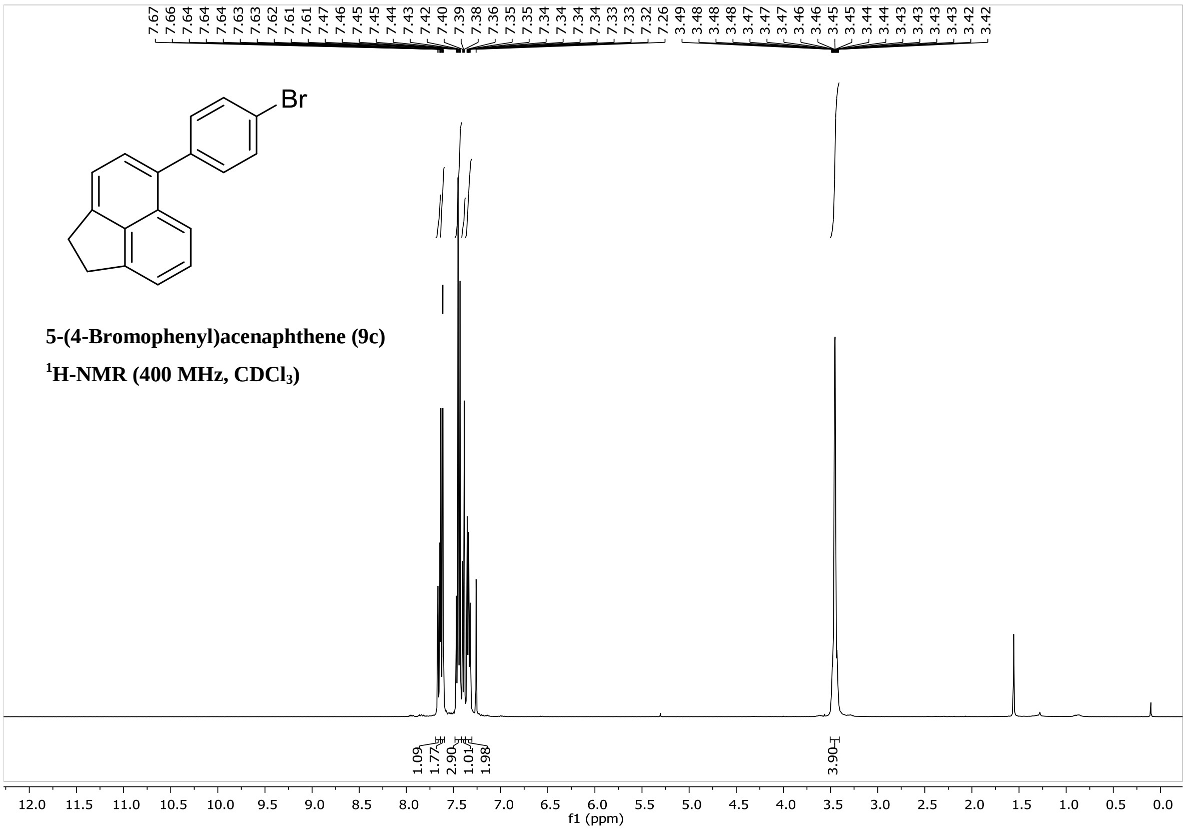

5-(4-Bromophenyl)acenaphthene (9c)

^{1}H-NMR (400 MHz, $CDCl_3$)

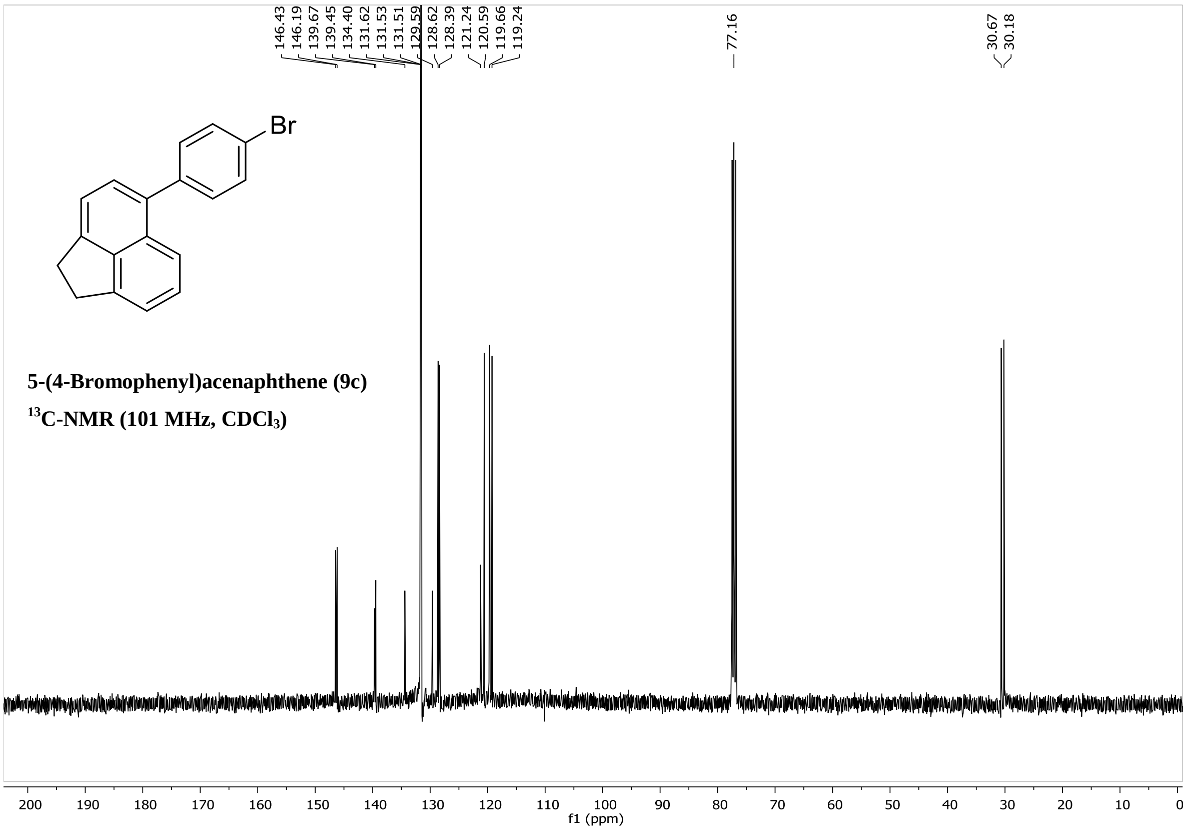

5-(4-Bromophenyl)acenaphthene (9c)

^{13}C-NMR (101 MHz, $CDCl_3$)

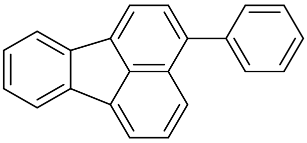

3-Phenylfluoranthene (10a) + regioisomer

^{1}H-NMR (400 MHz, $CDCl_3$)

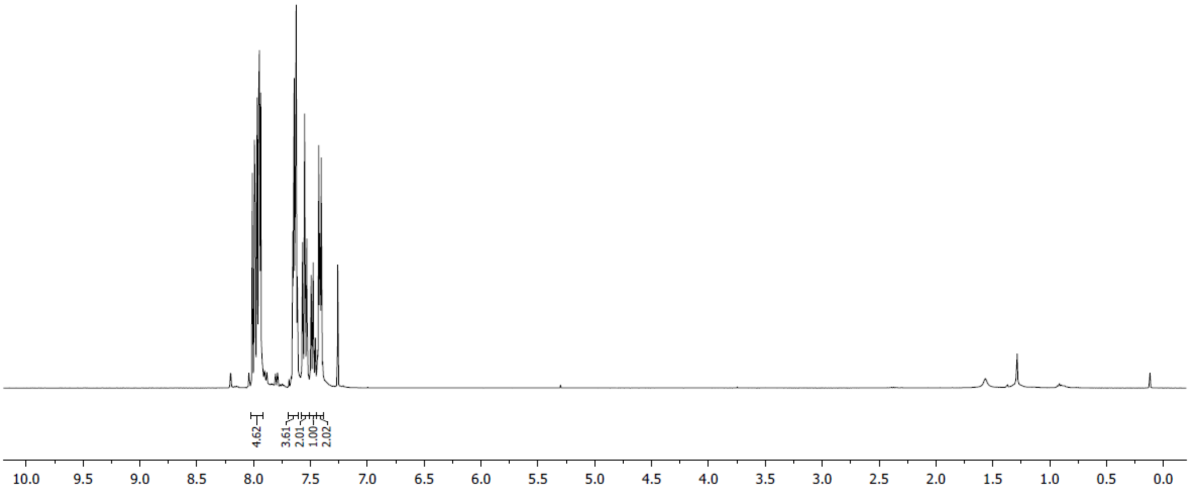

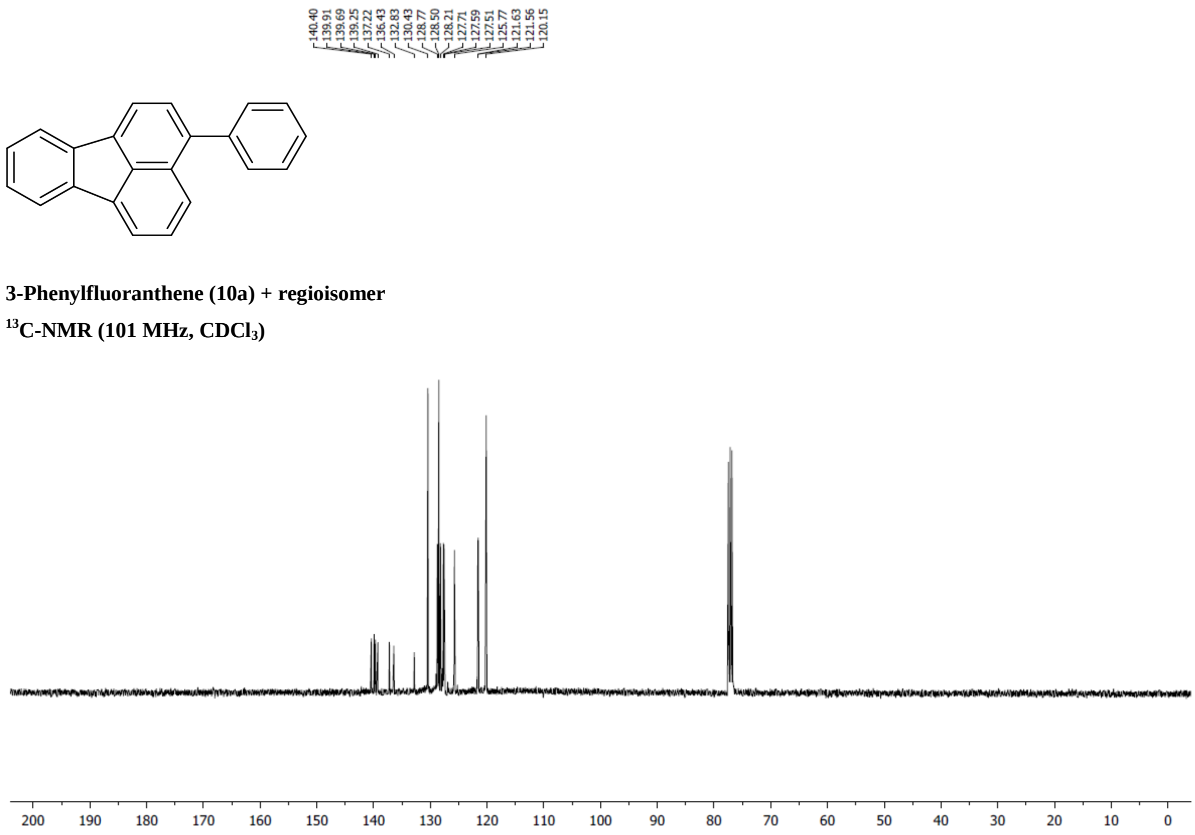

3-Phenylfluoranthene (10a) + regioisomer

13C-NMR (101 MHz, CDCl$_3$)

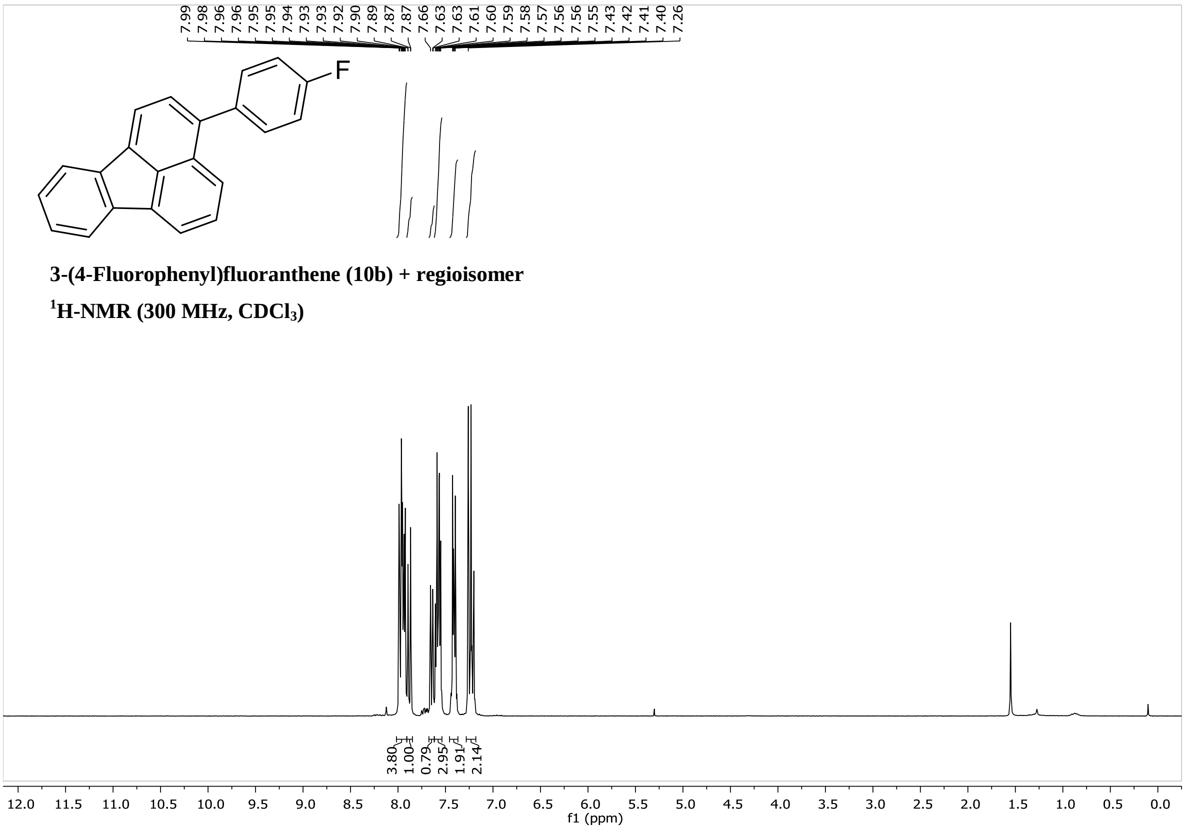

3-(4-Fluorophenyl)fluoranthene (10b) + regioisomer

1H-NMR (300 MHz, CDCl$_3$)

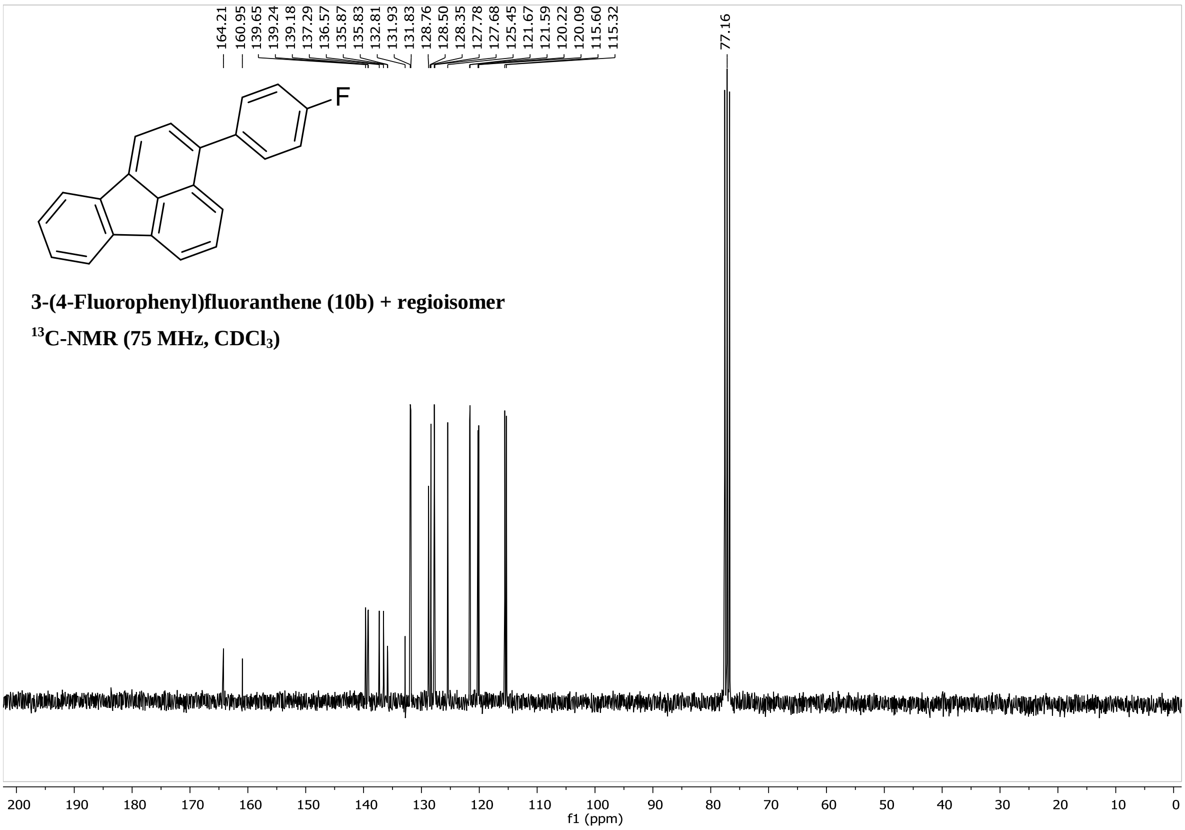

3-(4-Fluorophenyl)fluoranthene (10b) + regioisomer

^{13}C-NMR (75 MHz, $CDCl_3$)

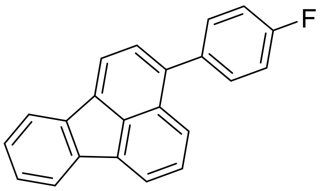

3-(4-Fluorophenyl)fluoranthene (10b) + regioisomer

^{19}F-NMR (282 MHz, $CDCl_3$)

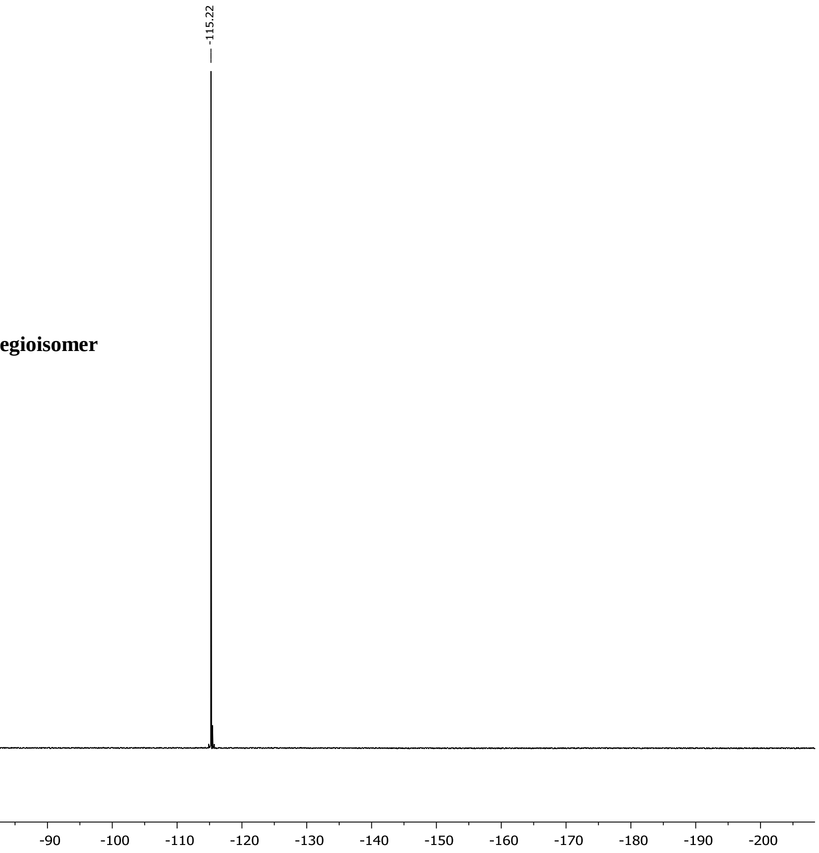

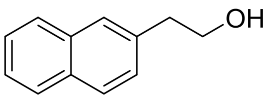

2-(2-Naphthyl)ethan-1-ol

^{1}H-NMR (400 MHz, $CDCl_3$)

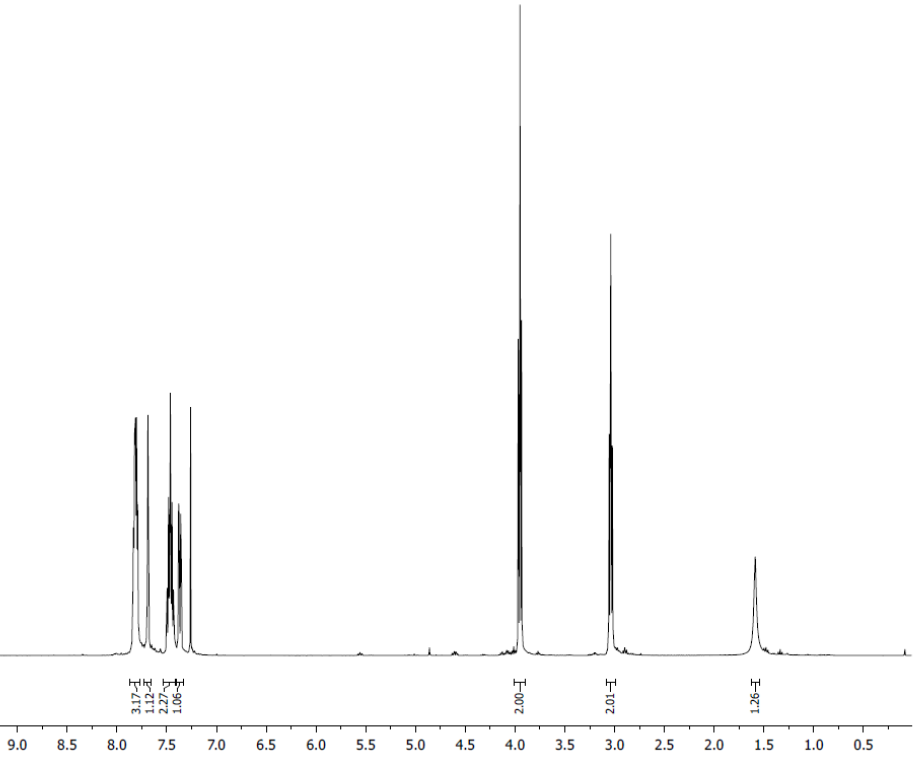

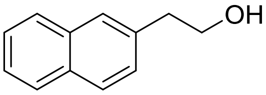

2-(2-Naphthyl)ethan-1-ol

^{13}C-NMR (101 MHz, $CDCl_3$)

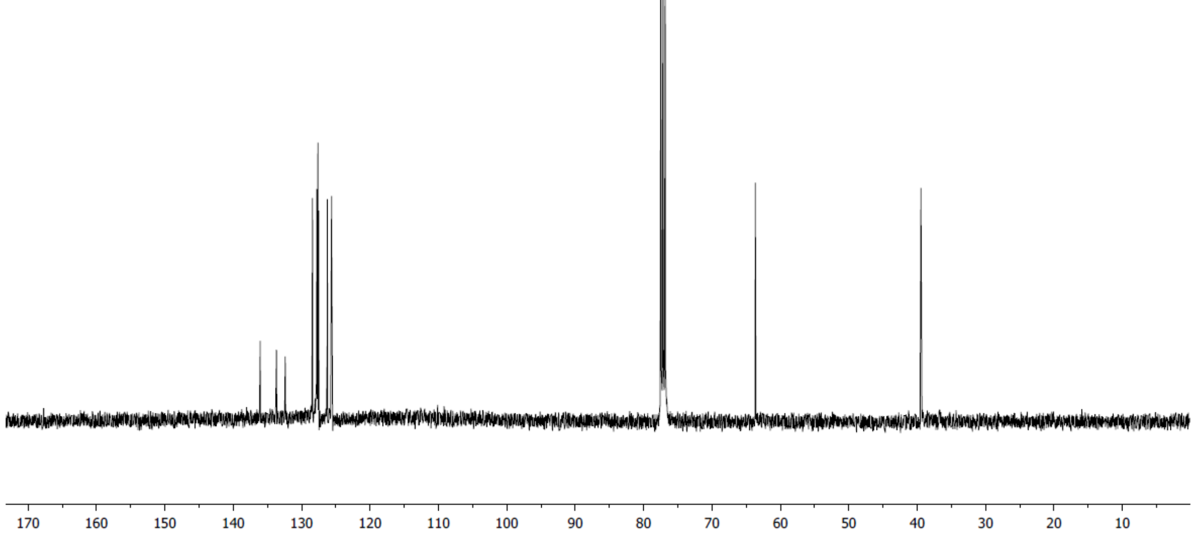

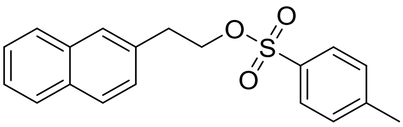

2-(2-Naphthyl)ethyl tosylate

^{1}H-NMR (400 MHz, $CDCl_3$)

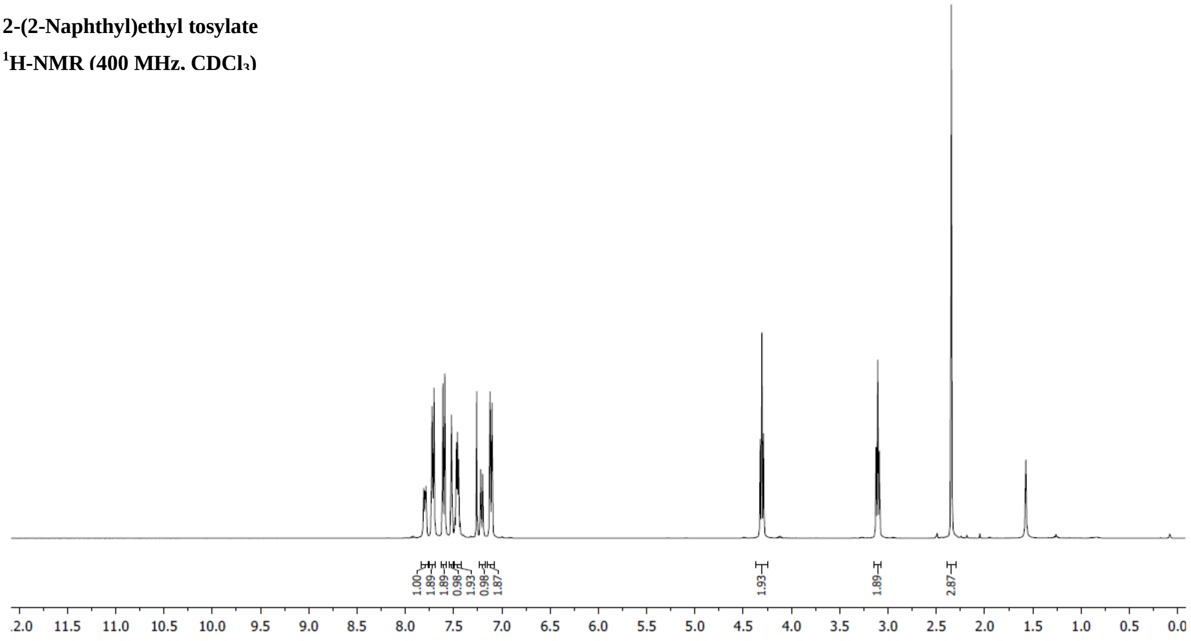

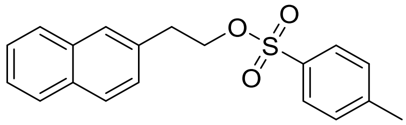

2-(2-Naphthyl)ethyl tosylate

^{13}C-NMR (101 MHz, CDCl$_3$)

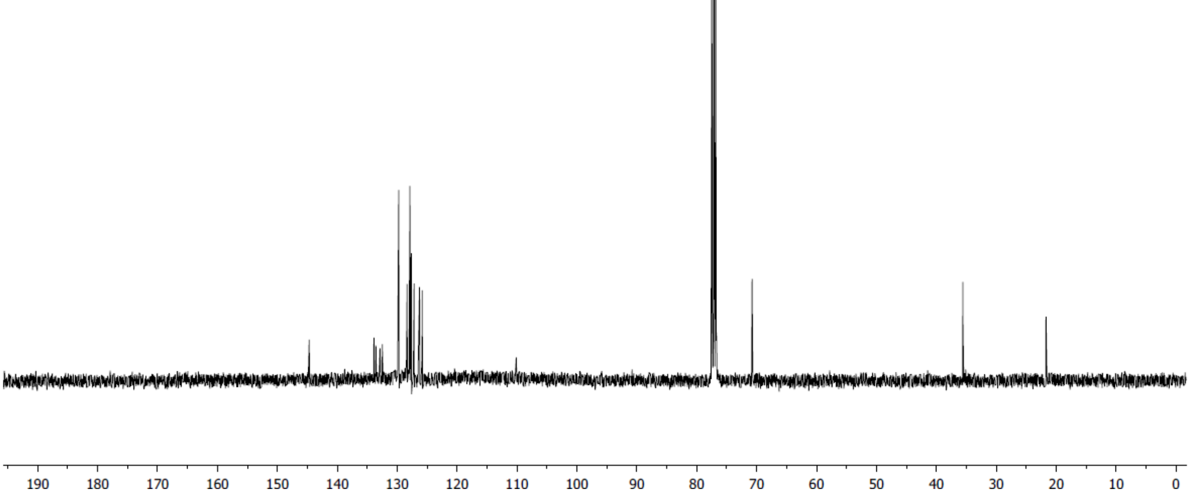

10. 0 References

[1] M. Bielawski, D. Aili, B. Olofsson, *J. Org. Chem.* **2008**, 73, 4602.

[2] A. M. Wagner, M. S. Sanford, *Org. Lett*. **2011,** *13*, 288.

[3] Storr, T. E.; Greaney, M. F. *Org. Lett.* **2013**, *15*, 1410.

[4] Kieffer, M.; Chuang, K.; Reisman, S. *Chem. Sci.*, **2012**, *3*, 3170.

[5] Phipps, R. J.; Grimster, N. P.; Gaunt, M. J.; *J. Am. Chem. Soc.* **2008**, *130*, 8172.

[6] Castro, S.; Fernández, J. J.; Vicente, R.; Fañanás, F. J.; Rodriguez, F. *Chem. Commun.* **2012**, *48*, 9089.

[7] Kobayashi, O.; Uraguchi, Y.; Yamakawa, T. *Org. Lett*. **2009**, *11*, 2679.

[8] Anbarasan, P.; Neumann, H.; Beller, M. *Chem. Asian J.* **2010**, *5*, 1775.

[9] Qin, C.; Lu, W. *J. Org. Chem.* **2008**, *73*, 7424-7427; J. Hassan, C. Hathroubi, C. Gozzi, M. Lemaire, *Tetrahedron* **2001**, *57*, 7845.

[10] Iwai, T.; Tanaka, R.; Harada, T.; Sawamura, M. *Chem. Eur. J.* **2014**, *20*, 1057.

[11] Chung, K. H.; So, C. M.; Wong, S. M.; Luk, C. H.; Zhou, Z.; Lau, C. P.; Kwong, F. Y. *Chem. Commun.* **2012**, *48*, 1967.

[12] Mochida, K.; Kawasumi, K.; Segawa, Y.; Itami, K. *J. Am. Chem. Soc.* **2011**, *133*, 10716.

[13] Kawai, H.; Kobayashi, Y.; Oi, S. *Chem. Commun.* **2008**, 1464.

[14] Sugihara, Y.; Takeda, H.; Nakayama, J. *Eur. J. Org. Chem.* **1999**, 597.

[15] Kawasumi, K.; Mochida, K.; Kajino, T.; Segawa, Y.; Itami, K. *Org. Lett.* **2012**, *14*, 418.

[16] Morales-Serna, J. A.; García-Ríos, E.; Bernal, J.; Paleo, E.; Gaviño, R.; Cárdenas, J. Synthesis **2011**, *9*, 1375-1382.

[17] Tang, D.-T. D. Collins, K. D.; Ernst, J. B.; Glorius, F. *Angew. Chem. Int. Ed*. **2014**, *53*, 1809

[18] Otwinowski, Z.; Minor, W.; *Methods Enzymol.* **1997**, *276*, 307.

[19] Otwinowski, Z.; Borek, D.; Majewski, W.; Minor, W. *Acta Crystallogr. Sect. A* **2003**, *59*, 228.

[20] Sheldrick, G. M. *Acta Crystallogr. Sect. A* **1990**, *46*, 467.

[21] Sheldrick, G. M. *Acta Crystallogr. Sect. A* **2008**, *64*, 112.

11.0 X-ray data

X-Ray diffraction: Data sets were collected with a Nonius KappaCCD diffractometer. Programs used: data collection, COLLECT (Nonius B.V., 1998); data reduction Denzo-SMN[18]; absorption correction, Denzo[19]; structure solution SHELXS-97[20]; structure refinement SHELXL-97[21]. R-values are given for observed reflections, and wR2 values are given for all reflections.

11.1 1-Phenylanthracene (6a)

CCDC 1013416

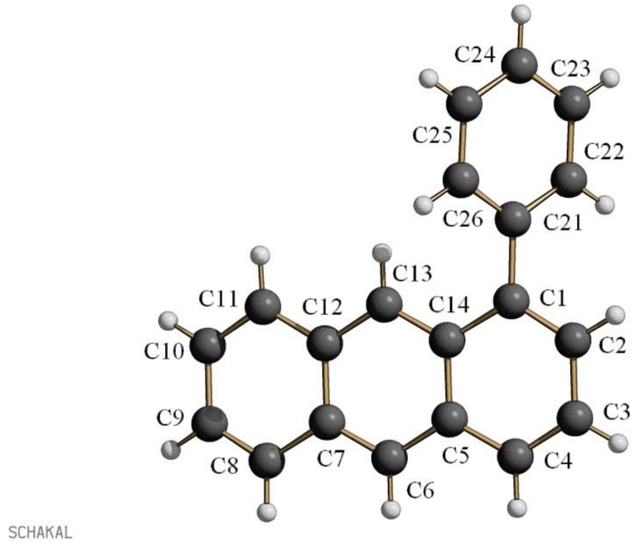

X-ray crystal structure analysis of 1-phenylanthracene (17): formula $C_{20}H_{14}$, M = 254.31, colourless crystal, 0.40 x 0.25 x 0.20 mm, a = 9.6321(1), b = 11.0943(1), c = 14.7022(1) Å, α = 67.862(1), β = 79.883(1), γ = 69.219(1)°, V = 1359.0(1) Å^3, ρ_{calc} = 1.243 gcm^{-3}, μ = 0.532 mm^{-1}, empirical absorption correction (0.815 ≤ T ≤ 0.901), Z = 4, triclinic, space group $P\bar{1}$ (No. 2), λ = 1.54178 Å, T = 223(2) K, ω and φ scans, 15068 reflections collected (±h, ±k, ±l), [(sinθ)/λ] = 0.60 Å^{-1}, 4558 independent (R_{int} = 0.029) and 4312 observed reflections [I>2σ(I)], 361 refined parameters, R = 0.038, wR^2 = 0.102, max. (min.) residual electron density 0.13 (-0.11) e.Å^{-3}, hydrogen atoms calculated and refined as riding atoms.

11.2 1-Phenyltriphenylene (5a)

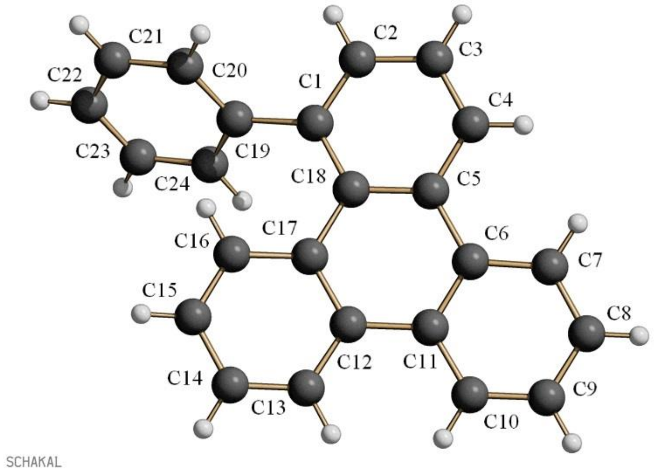

CCDC 1013417

X-ray crystal structure analysis of 1-phenyltriphenylene (20): formula $C_{24}H_{16}$, M = 304.37, colourless crystal, 0.17 x 0.07 x 0.02 mm, a = 19.1157(9), b = 5.7556(3), c = 29.6669(1) Å, β = 108.002(3)°, V = 3104.2(2) Å^3, ρ_{calc} = 1.303 gcm^{-3}, μ = 0.559 mm^{-1}, empirical absorption correction (0.911 ≤ T ≤ 0.988), Z = 8, monoclinic, space group $P2_1$/c (No. 14), λ = 1.54178 Å, T = 223(2) K, ω and φ scans, 38915 reflections collected (±h, ±k, ±l), [(sinθ)/λ] = 0.60 Å^{-1}, 5320 independent (R_{int} = 0.093) and 3678 observed reflections [I>2σ(I)], 434 refined parameters, R = 0.049, wR^2 = 0.144, max. (min.) residual electron density 0.17 (-0.17) e.Å^{-3}, hydrogen atoms calculated and refined as riding atoms.
